# Enhancement of CRISPR-Cas12a system through universal circular RNA design

**DOI:** 10.1016/j.crmeth.2025.101076

**Published:** 2025-06-16

**Authors:** Jiaqi Wang, Wei Zhang, Wentao Li, Qinyuan Xie, Ziyu Zang, Chaoxing Liu

**Affiliations:** 1 Guangdong Provincial Key Laboratory of Digestive Cancer Research, Digestive Diseases Center, Scientific Research Center, The Seventh Affiliated Hospital of Sun Yat-sen University, Shenzhen, Guangdong 518107, P.R. China; 2 Department of Clinical Laboratory, The Seventh Affiliated Hospital of Sun Yat-sen University, Shenzhen, Guangdong 518107, P.R. China; 3 School of Medicine, Sun Yat-sen University, Shenzhen, Guangdong 518107, P.R. China

**Keywords:** circular RNA, CRISPR-Cas12a, one-pot detection, DNA modifications, base excision repair

## Abstract

Precise control of Cas12a activity is crucial to address incompatibility in isothermal amplification-CRISPR-Cas12a one-pot nucleic acid detection. We developed a light-triggerable circular RNA system for dynamic LbCas12a regulation. By employing circular CRISPR guide RNA (crRNA) or a split circular universal direct repeat region with a replaceable spacer, we resolved the incompatibility between isothermal amplification and CRISPR detection. This system demonstrated robust performance in detecting trace nucleic acids in clinical samples. Furthermore, DNA modifications on circular crRNA enabled CRISPR-Cas12a regulation via base excision repair (BER) enzymes, offering potential for BER enzyme detection and modulation of LbCas12a cleavage activity by BER enzymes. This programmable strategy holds promise for selective gene editing in cells with elevated BER enzyme expression, such as uracil DNA glycosylase (UDG) in colon cancer cells. The circular RNA-assisted approach represents a resource-efficient method with significant potential for medical diagnostics and future clinical gene therapy applications.

## Highlights

• Developed a light-activated circular RNA system for dynamic LbCas12a regulation
• Enabled high-sensitivity, specific detection of trace nucleic acids in clinical samples
• Utilized DNA-modified circular RNA for precise Cas12a control via BER enzymes
• Proposed a strategy for selective DNA cleavage with high BER expression

## Motivation

The CRISPR-Cas12a system faces limitations in its use in clinical diagnostics, including incompatibility with isothermal amplification and lack of precise activity control. These challenges hinder its application in one-pot nucleic acid detection and non-nucleic acid target analysis. To address these issues, we developed a universal circular RNA system for dynamic LbCas12a regulation. This approach resolves amplification-detection incompatibility and introduces a mechanism for CRISPR-Cas12a control via light triggering or base excision repair enzymes. Our method bridges gaps by offering transformative potential for precise diagnostics and expanding CRISPR-based applications in clinical settings.

## Graphical abstract

**Figure:**
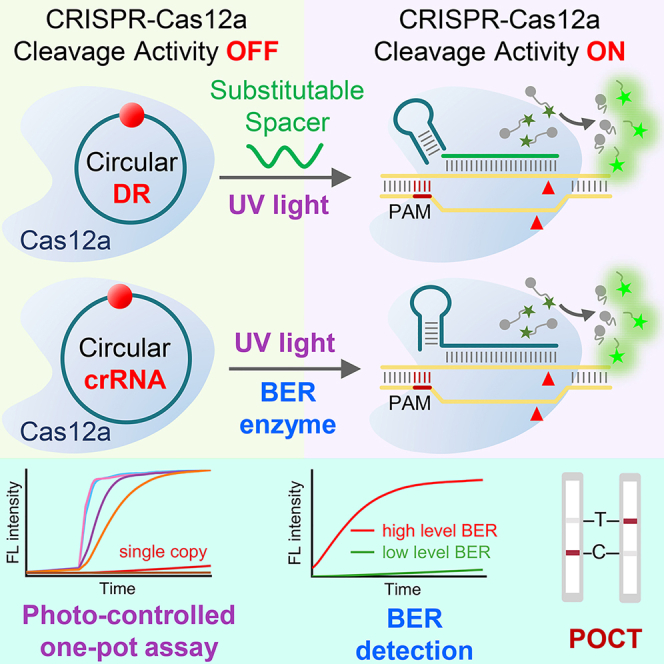

## Teaser

Wang et al. present a universal circular RNA design enabling precise control of Cas12a activity via UV light or base excision repair enzymes. This system achieves high-sensitivity detection of trace nucleic acids in clinical samples and offers potential for non-nucleic acid target analysis, advancing diagnostics and targeted therapeutic applications.

## Introduction

The CRISPR-Cas12a system is a natural immune system found in prokaryotes, such as the Lachnospiraceae bacterium ND2006 (Lb), Acidaminococcus sp. BV3L6 (As) and *Francisella novicida* (Fn).^1^^,^^2^^,^^3^ The Cas12a protein, also known as Cpf1 in this system is a nuclease that, with the assistance of CRISPR guide RNA (crRNA), can recognize and cleave foreign invasive DNA.^4^ One remarkable feature of this system is its *cis*-cleavage activity, enabling it to effectively cleave target double-stranded DNA (dsDNA).^5^^,^^6^ This makes it a potentially powerful tool in the field of gene editing.^7^^,^^8^^,^^9^^,^^10^ Additionally, activated CRISPR-Cas12a also possesses *trans*-cleavage activity,^11^^,^^12^ enabling the cleavage of non-specific single-stranded DNA (ssDNA),^13^^,^^14^ λDNA,^15^ and 3′ overhang dsDNA.^16^ A highly effective nucleic acid detection platform has been developed utilizing the *trans*-cleavage activity of CRISPR-Cas12a.^17^^,^^18^^,^^19^ This technology is regarded as a powerful and immensely promising tool for nucleic acid detection, potentially surpassing qPCR technology to become the new gold standard.^20^^,^^21^^,^^22^

DETECTR^14^ and HOLMES,^17^ methods developed in 2018 using CRISPR-Cas12a, marked the beginning of a period of rapid development for the CRISPR-Cas12a nucleic acid detection system. Typically, CRISPR-Cas12a-based nucleic acid detection systems require specific crRNA design, including a universal direct repeat (DR) region and a spacer region (18–23 nt).^3^ The Cas12a protein interacts with the crRNA to form a complex. Activation of this complex occurs when it binds to the target DNA, leading to non-specific cleavage of ssDNA through *tran*s-cleavage activity.^13^^,^^19^ This activity not only cleaves the target DNA but also ssDNA reporters, such as fluorophore and quencher (F-Q) (Table S1), which contains a fluorophore (FAM) at one end and a quencher (BHQ1) at the other (Figure S9H). This cleavage event leads to the release of a fluorescent signal, as demonstrated in previous studies.^14^^,^^17^ Monitoring the change in fluorescent intensity allows for the determination of the target DNA content. Combining this technology with isothermal amplification and lateral flow chromatography has proven effective for virus detection, such as COVID-19,^18^^,^^23^^,^^24^^,^^25^^,^^26^^,^^27^^,^^28^ providing excellent results and societal value. Continuous improvement has expanded the application of the CRISPR-Cas12a system to detect RNA (e.g., SAHARA,^29^ asymmetric CRISPR technology^30^) and other non-nucleic acid targets.^31^^,^^32^

The CRISPR-Cas12a system holds great potential in the field of clinical nucleic acid testing, offering improved detection sensitivity, reduced reliance on large instruments, lower testing costs, and faster detection speed.^20^^,^^33^^,^^34^^,^^35^ It allows for early screening of low copy-number viruses and tumor nucleic acid biomarkers.^30^^,^^36^^,^^37^ However, integrating the isothermal amplification system with the CRISPR reaction in a single tube can hinder nucleic acid amplification efficacy due to the *trans*-cleavage activity of the Cas12a protein, particularly with low copy-number inputs. This can impair amplification efficiency and decrease detection sensitivity.^25^^,^^38^ Initial versions of DETECTR^14^ and HOLMES^17^ required separate tubes for amplification and the CRISPR reaction, increasing the complexity of the diagnostic procedure and the potential for false positive results due to liquid transfer and aerosol contamination.^39^ Various optimization strategies have been explored to address these challenges, such as physical segregation of reagents,^40^^,^^41^ specialized buffers,^42^ selection of suboptimal protospacer adjacent motif (PAM) sites,^43^ and phase-separation solutions.^44^ However, conventional approaches have shown limitations or hindered the CRISPR reaction in specific clinical specimen tests, leading to unresolved compatibility issues.^25^^,^^38^ Through the implementation of temporary-deactivated light-start CRISPR strategies,^45^^,^^46^^,^^47^^,^^48^ researchers have achieved high-sensitivity detection without the need for separate reaction tubes or reagent transfer.^25^^,^^38^^,^^49^^,^^50^ Furthermore, the validation of the inhibitory effect of circular crRNA’s topological structure on Cas12a activity offers promising prospects for completely overcoming the compatibility issues between isothermal amplification and CRISPR-Cas12a.^31^^,^^51^ However, previous Cas12a crRNA circularization methods focused on canonical nucleic acids without chemical modifications, relying solely on nucleic acid enzyme for Cas protein reactivation, limiting their compatibility with clinical isothermal amplification and CRISPR integration.^31^ Existing photocontrolled methods, although promising for clinical nucleic acid detection, require target-specific design and synthesis, including uracil substitution^38^—a challenge for uracil-free sequences—or the addition of complementary sequences,^25^ increasing complexity and reducing versatility. A universal strategy that necessitates only a single modification of nucleic acids for the detection of arbitrary targets is urgently required.

**Figure 1:**
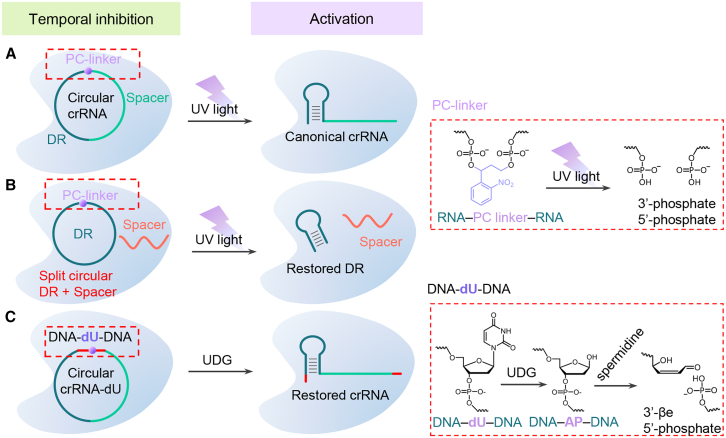
Development and optimization of circular RNA for enhanced CRISPR-Cas12a-based detection (A) Schematic of circular crRNA incorporating a commercially available PC linker. (B) Design of a universal circular DR region with a PC linker and a replaceable spacer region, enabling target-specific detection via a photocontrolled one-pot strategy to address compatibility challenges between isothermal amplification and CRISPR-Cas12a. (C) Introduction of DNA modifications into circular crRNA to regulate the CRISPR-Cas12a system using base excision repair (BER) enzymes.

Here, we present a method called CIRCLE (enhancement of CRISPR-Cas12a system through universal circular RNA design) (Figure 1). This method focuses on incorporating a photocleavable (PC) linker into either the circular crRNA or universal circular DR. We hypothesize that these circular RNA designs effectively suppress LbCas12a activity, while ultraviolet (UV) light-mediated cleavage of the PC linker restores the native topology and reinstates the CRISPR-Cas12a cleavage function. This design promises to address the critical challenge of incompatibility between CRISPR systems and isothermal amplification of clinical trace nucleic acids, offering a robust platform for precise molecular control. Additionally, we propose introducing DNA modifications within the circular crRNA, such as deoxyuridine (dU). We hypothesize that circular crRNA with dU similarly inhibits LbCas12a activity, which can be restored by the corresponding base excision repair (BER) enzymes, such as uracil DNA glycosylase (UDG). Building upon this principle, we propose developing a series of BER enzyme detection methods by incorporating specific modified bases into the circular crRNA that correspond to target BER enzymes. This proposed circular system demonstrates several distinct advantages. The circular DR with a PC linker represents a universal platform requiring only a single design and synthesis, yet maintains versatility through its ability to pair with any spacer for target-specific detection. Furthermore, the inherent stability of circular RNA architecture surpasses that of linear RNA counterparts, significantly enhancing its suitability for long-term preservation and clinical implementation. Moreover, our system has the potential to expand the application of CRISPR-based detection beyond nucleic acids and offers a promising approach for precision gene editing by regulating the *cis*-cleavage activity of LbCas12a through the modulation of specific BER enzyme levels.

## Results

### Circular crRNA with PC linker for UV-controlled regulation of LbCas12a cleavage activity

**Figure 2:**
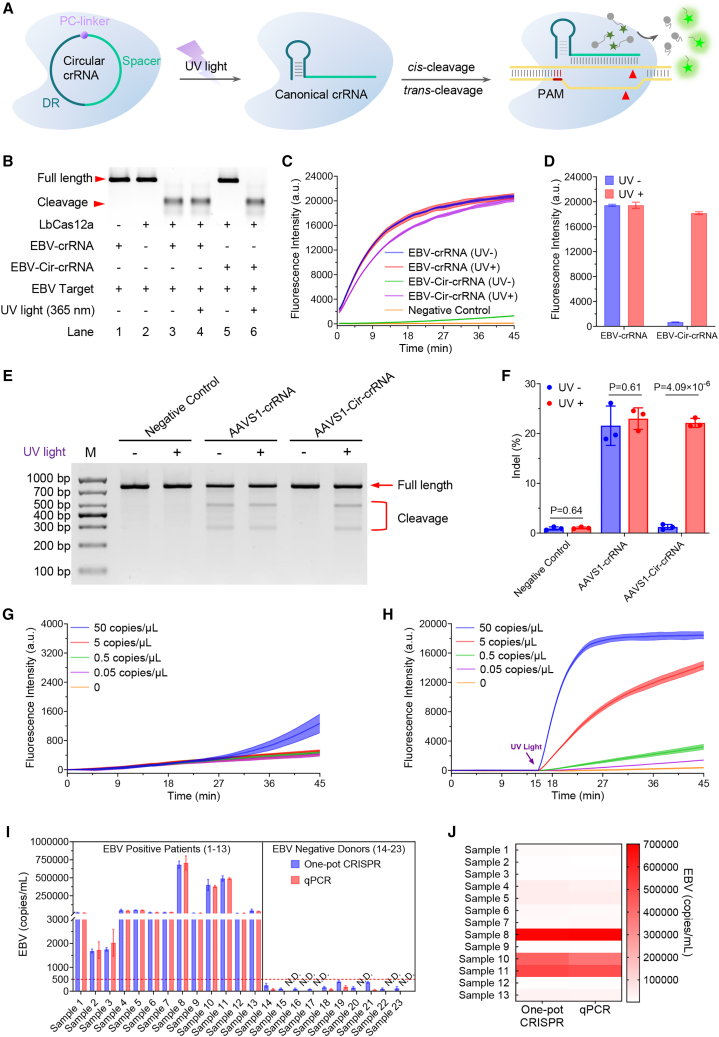
Verification of photoinducible regulation of CRISPR-Cas12a *trans*- and *cis*-cleavage activities by circular crRNA with a commercially available PC linker under 30-s UV light exposure (A) Schematic depiction reveals that circular crRNA with a PC linker can be disintegrated under UV light, reverting to conventional crRNA, further restoring the *trans*- and *cis*-cleavage activities of LbCas12a. (B) A 2% agarose gel analysis verifies that circular crRNA with a PC linker can suppress LbCas12a *cis*-cleavage activity. Notably, 30 s under UV light can restore this activity. (C) Fluorescence data showcase that circular crRNA can inhibit LbCas12a *trans*-cleavage activity. Again, exposure to UV light for 30 s is capable of bringing back this activity. (D) The bar graph quantitatively compares the fluorescence values of classic and circular crRNAs at the 45-min time point under both UV irradiation and non-irradiation conditions, as shown in (C). (E) T7E1 nuclease assays illustrate that the circular crRNA structure suppresses CRISPR-Cas12a gene editing at the AAVS1 locus in HEK293T cells in the absence of UV light. However, exposure to UV light induces the breakage of the circular crRNA, thereby restoring the functionality of CRISPR-Cas12a. (F) A bar chart presents the percentage of indels (insertion or deletion mutations) observed under different conditions: negative control, canonical crRNA, and circular crRNA with and without UV exposure. (G and H) Comparative analysis of detection limit between the conventional RPA-CRISPR-Cas12a one-pot detection method (G) and the circular crRNA-assisted RPA-CRISPR-Cas12a photocontrollable one-pot nucleic acid detection system we adopted for our current work (H) is depicted. (I and J) The bar chart (I) and heatmap (J) illustrate the comparison between the traditional qPCR method and our current circular crRNA-assisted photocontrollable one-pot nucleic acid detection method in detecting EBV-infected blood samples in clinical settings. N.D., not detected. The red dashed line in (I) denotes the clinically established threshold of 500 copies/mL for EBV-DNA viral load. Values exceeding this threshold are indicative of EBV positivity in clinical diagnostics, as supported by the current literature and diagnostic standards.^52^^,^^53^ This demarcation serves as a critical reference for interpreting EBV infection status in both experimental and clinical contexts. In all panels, error bars represent the mean ± standard deviation (SD) (*n* = 3). *p* > 0.05 indicates no significant difference between groups.

To investigate the inhibitory effects of circular crRNA incorporating a PC linker on both *cis*- and *trans*-cleavage activities of LbCas12a, along with its potential reactivation in photocontrolled isothermal amplification-CRISPR-Cas12a nucleic acid detection systems, we designed and synthesized an RNA sequence (EBV-Cir-crRNA precursor, Table S1) targeting Epstein-Barr virus (EBV) containing a commercially available PC linker (Figure 2A). The circular crRNA precursor was designed with a 5′-phosphate and the PC linker inserted in the middle (Figure S1A). Circularization of the crRNA precursor was achieved using T4 RNA ligase 1, followed by elimination of linear molecules through RNase R treatment. Successful circularization was confirmed using 18% denaturing polyacrylamide gel electrophoresis (PAGE) (Figure S1B). Furthermore, exposure of the PC linker-modified circular crRNA (EBV-Cir-crRNA) to UV light (λ = 365 nm, 35 W) resulted in rapid and efficient breakage of the PC linker. Complete breakage was achieved within 30 s and remained stable for 20 min (Figure S1C). These findings demonstrate the feasibility of adjusting the conformation of commercially available PC linker-modified circular crRNA through brief UV light exposure.

Subsequently, we investigated the inhibitory effect of circular crRNA with a PC linker on CRISPR-Cas12a *cis*-cleavage activity and explored the possibility of restoring this activity through UV irradiation-induced breakage of the PC linker. We synthesized a 357-bp target DNA sequence specific to the EBV (EBV target) for experimental purposes.^38^ We compared the *cis*-cleavage activity of EBV-Cir-crRNA and conventional EBV crRNA (Table S1) using 2% agarose gel analysis under varying conditions. Our results showed that EBV-Cir-crRNA effectively inhibited CRISPR-Cas12a *cis*-cleavage activity due to its altered configuration (Figure 2B, lanes 1, 2, 3, and 5). This inhibition was completely reversed when the circular crRNA was subjected to short-duration UV irradiation, breaking the PC linker and restoring its canonical form (Figure 2B, lane 6). Notably, a 30-s UV irradiation had no impact on the system (Figure 2B, lanes 3, 4, and 6). These findings highlight the potential of circular crRNA with a PC linker as a versatile tool in controlling CRISPR-Cas12a cleavage activity.

Furthermore, we investigated the inhibitory effect of circular crRNA with a PC linker on the *trans*-cleavage activity of CRISPR-Cas12a and explored the potential for restoring this activity by UV irradiation-induced cleavage of the PC linker. Comparative analysis of canonical EBV crRNA and EBV-Cir-crRNA with the addition of F-Q (Table S1, λ_ex_: 494 nm, λ_em_: 518 nm).^14^^,^^17^ Our results (Figures 2C and 2D) showed that EBV-Cir-crRNA did not induce LbCas12a *trans*-cleavage activity without UV irradiation. However, under UV irradiation, breakage of the PC linker successfully restored CRISPR-Cas12a *trans*-cleavage activity. Short-term UV irradiation for 30 s had no significant effect on the system. These results provide strong evidence for the effective inhibition of LbCas12a cleavage activities by commercially available PC linker-modified circular crRNA. We demonstrated that a brief 30-s exposure to UV irradiation can successfully restore LbCas12a cleavage function.

To evaluate the photostability of circular crRNA containing the PC linker under experimental conditions, we performed stability assessments without specialized light protection, replicating standard laboratory illumination. Specifically, 100 ng EBV-Cir-crRNA was incubated in transparent 0.2-mL microcentrifuge tubes on ice for 0, 1, 2, 4, 8, 12, and 24 h. For comparative analysis, 100 ng UV-induced EBV-Cir-crRNA cleavage product served as a positive control for PC linker disruption. All samples were evaluated using 12% denaturing PAGE (150 V, 30 min) to assess PC linker integrity. The results confirmed the structural stability of circular RNA under normal laboratory lighting (Figure S1D). Furthermore, *trans*-cleavage activity assays comparing standard laboratory light-exposed and unexposed samples over 24 h showed no significant variation in LbCas12a activation, thereby validating the photostability of our circular RNA design (Figures S1E and S1F). To further investigate the enhanced environmental stability of our circular RNA (EBV-Cir-crRNA) relative to linear EBV crRNA, 100 ng of each RNA was incubated with 2 μL Dulbecco’s Modified Eagle Medium supplemented with 10% fetal bovine serum (FBS) at 37°C for 0, 5, 10, 20, 40, and 60 min. Analysis by 12% denaturing PAGE (150 V, 30 min) revealed that linear crRNA underwent significant degradation within 10 min and was completely degraded by 40 min, whereas circular RNA retained its structural integrity throughout the 60-min incubation (Figure S1G). These findings highlight the superior stability of our circular RNA design, underscoring its potential advantages for commercial production, transportation, and practical applications compared to conventional linear RNA constructs.

CRISPR-Cas12a gene editing holds significant promise for the treatment of numerous human genetic diseases.^10^^,^^51^^,^^54^ The adeno-associated virus integration site 1 (AAVS1), located on chromosome 19 of the human genome, is recognized as a “safe harbor” site.^55^ This site is well validated for ensuring the intended functionality of integrated DNA fragments due to its open chromatin structure, which facilitates normal transcription of introduced genes. Importantly, the insertion of exogenous target sequences at the AAVS1 site has no known adverse effects on cellular function, making it a critical locus for *in vivo* gene editing studies.^56^^,^^57^^,^^58^ In our study, we targeted the AAVS1 site with both a classical crRNA (AAVS1 crRNA, Table S1) and a circular crRNA (AAVS1-Cir-crRNA, Table S1). We systematically investigated the editing efficiency of these crRNAs in HEK293T cells under conditions with and without UV light (λ = 365 nm) exposure. First, we validated *in vitro* that AAVS1-Cir-crRNA-assisted CRISPR-Cas12a could not activate LbCas12a function in the absence of UV light. Conversely, after 30 s of UV exposure, the circular structure of AAVS1-Cir-crRNA reverted to its native state and reactivated LbCas12a function to a level comparable to that of classical crRNA (Figures S2A–S2C). Second, to evaluate the effects of UV light on cellular viability, HEK293T cells were exposed to UV irradiation for 0, 1, 2, 3, 5, and 10 min. Cell viability remained unchanged after 0–3 min of exposure, while a marginal decline was observed after 5 min (Figure S2E). To assess the genome-wide impact of UV irradiation, particularly its potential to induce DNA damage, a comet assay was performed. HEK293T cells were cultured under standard conditions and exposed to UV light (λ = 365 nm) for 1, 2, 3, 5, and 10 min, with the UV lamp positioned 15 cm above culture dishes (with lids) placed on ice. Untreated cells served as the negative control, and cells treated with 50 μM H_2_O_2_ for 1 h were used as the positive control. Post-exposure, cells were trypsinized, washed twice with PBS, and resuspended in PBS at a concentration of 1 × 10^6^ cells/mL. The comet assay was conducted following the manufacturer’s protocol (DNA Damage Comet Assay Kit, GBCBIO Technologies, catalog no. G7668). Fluorescence microscopy was used for image acquisition, and automated analysis was performed using the OpenComet plugin in ImageJ. The results revealed no significant DNA damage after 0–5 min of UV exposure; however, genome-wide damage was detected after 10 min (Figure S2D). Based on these findings, a mild condition of 365 nm UV irradiation for 3 min was selected for subsequent gene editing experiments, as it does not significantly compromise cell viability or induce detectable DNA damage. Then, we delivered AAVS1-Cir-crRNA and LbCas12a into HEK293T cells using the TransIT-X2 Dynamic Delivery System (Mirus Bio, catalog no. MIR 6000). The cells were then exposed to UV light (λ = 365 nm) for either 3 min or not, followed by another 42 h of culture. The T7 endonuclease I (T7E1) assay^59^ was then used to measure the percentage of insertions or deletions (indels), which indicate non-homologous end joining events. Our results demonstrated that the classical crRNA achieved approximately 20% indels, independent of UV light exposure. In contrast, AAVS1-Cir-crRNA exhibited negligible editing activity without UV exposure, comparable to unedited samples. Upon UV light exposure, the editing efficiency of AAVS1-Cir-crRNA was comparable to that of classical crRNA (Figures 2E, 2F, S2F, and S2G). These findings confirm that UV light exposure can selectively regulate the CRISPR-Cas12a editing activity facilitated by circular RNA in living cells. This controlled gene editing strategy adds a valuable layer of precision, making it a promising tool for future *in vivo* genetic modification applications.

### Development of a one-pot CRISPR-Cas12a nucleic acid detection system with PC linker-containing circular crRNA

Based on the exciting findings mentioned above, we developed a one-pot CRISPR-Cas12a nucleic acid detection system based on circular crRNA combined with recombinase polymerase amplification (RPA) technology.^60^ After a 15-min isothermal amplification reaction at 37°C, 30 s of UV irradiation was used to initiate the CRISPR-Cas12a reaction, and fluorescence signals were recorded. Compared to canonical EBV crRNA (Figure 2G), our approach utilizing EBV-Cir-crRNA (Figure 2H) exhibited superior performance in detecting lower concentrations of the target DNA. The cleavage activity of LbCas12a degraded the EBV target during isothermal amplification, leading to the absence of detectable signals for low-concentration targets (Figure 2G; 0.05–5 copies/μL). Our system (Figure 2H) demonstrated a 100-fold improvement in detection limit compared to the conventional RPA-CRISPR-Cas12a system (Figure 2G).

Subsequently, we collected 13 blood samples from patients infected with EBV, as well as 10 samples from EBV^−^ donors, for further validation (Figures 2I and 2J). Worldwide, most individuals contract EBV in childhood, becoming lifelong asymptomatic carriers with low viral loads unless immunocompromised.^61^ Clinically, EBV-DNA levels above 500 copies/mL define positive cases.^52^^,^^53^ Initially, we extracted DNA from the blood samples and performed detection using the developed detection system with circular crRNA mentioned above. Utilizing the established standard curve by this system (Figures S3A and S3B), we calculated the copy number of the EBV in the clinic samples (Figures 2I and S3C). To evaluate performance, we compared our method with the gold standard qPCR (Figures S3D–S3F). In samples with high viral loads, our method exhibited detection consistency comparable to qPCR (Figures 2I and 2J). Notably, in samples with EBV levels below the clinical cutoff of 500 copies/mL, where qPCR often fails to provide precise quantification due to its higher detection limit, our method demonstrated superior sensitivity and reliable detection capabilities. This highlights its potential for enhanced accuracy in low-abundance viral load scenarios (Figures 2I, 2J, and S3). The PC linker-based crRNA circularization, while enabling trace nucleic acid detection, necessitates individual crRNA precursor re-circularization per target, substantially increasing costs. This highlights an urgent need for a universal circularization strategy applicable to diverse targets.

### Development of a one-pot CRISPR-Cas12a nucleic acid detection system through a universal photocontrolled circular DR design

**Figure 3:**
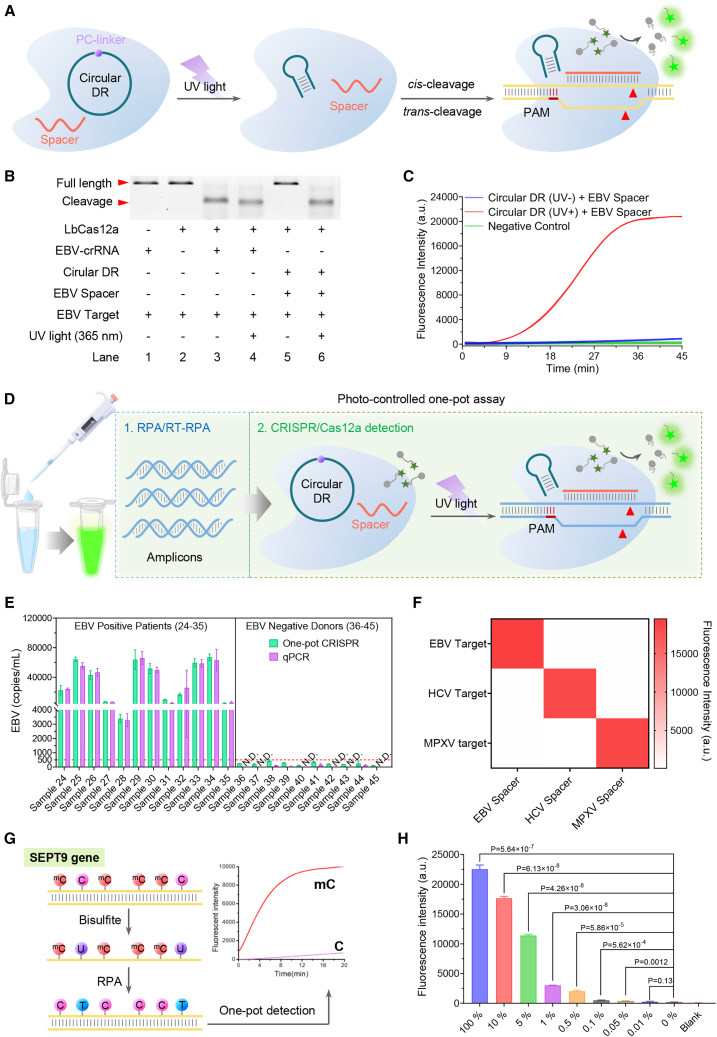
Validation of the effective regulation of the LbCas12a *cis*- and *trans*-cleavage activities by circular DR with a PC linker under UV light exposure (A) The schematic image reveals that UV light can break the circular DR containing a PC linker, which then reverts to its conventional form, subsequently restoring the *cis*- and *trans*-cleavage activities of LbCas12a with the help of different spacer. (B) A 2% agarose gel analysis verifies the ability of the circular DR and spacer to inhibit LbCas12a *cis*-cleavage activity effectively. This activity can be restored following 30 s of UV light exposure. (C) Fluorescence data demonstrate the inhibition of the *trans*-cleavage activity of LbCas12a by the circular DR with a PC linker. Again, a 30-s UV light exposure can restore this activity. (D) The schematic image portrays the RPA/RT-RPA-CRISPR LbCas12a photocleavable one-pot nucleic acid detection system assisted by circular DR. Changing the replaceable spacer can enable its application for different nucleic acid target detection. (E) Bar chart demonstrates a comparison between the traditional qPCR method and our current development, the circular DR-assisted RPA/RT-RPA-CRISPR-Cas12a photocleavable one-pot nucleic acid detection method for the detection of clinic EBV-infected blood samples. (F) Heatmap analysis indicates that universal circular DR, when paired with different spacer, can be utilized for highly selective detection of various targets in a single separate way. (G) Schematic representation of the current CRISPR-Cas12a-based assay for determining the methylation status of the SEPT9 gene, which is a nucleic acid biomarker in colorectal cancer. The feasibility of this method has been validated using synthetic hypermethylated (four sites) and non-methylated SEPT9 (Table S1). (H) The detection results of different methylation proportions of the SEPT9 gene. All experiments were performed in triplicate. Data are presented as mean ± SD (*n* = 3). *p* > 0.05 indicates no significant difference between groups.

Building upon experimental evidence demonstrating that split crRNA retains full functionality in Cas12a systems,^30^^,^^62^^,^^63^^,^^64^ we hypothesize that a circular DR with a PC linker could effectively inhibit both the *trans-* and *cis*-cleavage activities of Cas12a, with the potential for reactivation upon UV irradiation. That is, this circular DR region with a PC linker design could be universally employed, only modifying the spacer to detect different nucleic acid substrates (Figure 3A). To verify this hypothesis, we cyclized the DR sequence with a commercially available PC linker using the method previously described. The cyclization was confirmed by denaturing PAGE (Figure S4A). Experiments showed that 30-s UV light exposure could successfully restore the circular DR sequence to its canonical state (Figure S4B). Similarly, we have also demonstrated that the circular DR exhibits excellent stability and robust resistance to degradation (Figures S4C–S4F). We then put this discovery to test using a spacer targeting the EBV (EBV spacer, Table S1). The results indicate that when the circular DR is combined with the spacer, the enzymatic activity of CRISPR-Cas12a is completely abolished. However, upon exposure to UV light for 30 s, the circular DR returns to its usual form, restoring the activity of the LbCas12a (Figures 3B and 3C). We further augmented our system with RPA techniques, creating a one-pot testing system for EBV (Figure 3D). Our method was corroborated using 12 blood samples from EBV-infected and 10 blood samples from healthy individuals and the standard qPCR method (Figures 3E and S5). Results indicate our method can effectively be used for clinical detection of EBV.

To demonstrate the versatility of our design, we selected different target spacers like mpox (monkeypox) virus, hepatitis C virus, and EBV (Figures 3F and S4G–S4M). The results reveal that by using a universal circular DR with a PC linker and matching spacers, it is feasible to conduct one-pot isothermal amplification detection for different virus samples. Additionally, our method shows promise in early colon cancer detection. Previous research suggests that high methylation of the SEPT9 gene is a strong predictor of colon cancer.^65^ We synthesized positive control samples with high methylation SEPT9 sequences and negative control samples with non-methylation sequences (Table S1).^37^ After sodium bisulfite treatment, we used our established RPA-CRISPR-Cas12a system for detection. Figures 3G and 3H indicate that we have successfully detected high methylation of the SEPT9 gene, demonstrating that by changing the spacer, our method can detect not just multiple viral nucleic acid sequences, but also DNA modifications, such as 5-methylcytosine, that serve as colon cancer early detection biomarkers. Future endeavors will aim to expand the application of this system to detect a broader range of clinical samples.

### Development of a BER enzyme detection platform based on DNA modification-containing circular crRNA-assisted CRISPR-Cas12a system

BER enzymes are involved in various biological processes, including DNA repair, gene expression regulation, and genetic recombination.^66^ Additionally, DNA modifications are associated with specific BER enzymes, which interact with them.^66^ DNA, as the primary genetic information repository, is susceptible to alterations caused by epigenetic processes and external factors like chemicals, radiation, and metabolic byproducts.^67^^,^^68^ These alterations, such as base modifications, can spontaneously occur in DNA and affect cellular activities.^69^ Recently, BER enzymes that interact with these DNA modifications have gained significant attention.^70^ For example, UDG is a BER enzyme that specifically identifies and repairs dU, which is naturally present in genomic DNA.^71^ The apurinic/apyrimidinic (AP) site generated by UDG from dU can be eliminated through alpha or beta elimination,^72^ leading to a DNA strand break.^73^ This BER process maintains DNA integrity and stability by preventing incorrect base pair integration.^66^ Moreover, UDG plays a crucial role in detecting viral infections^74^^,^^75^ and can be used as a biomarker for colon cancer.^76^ Therefore, the development of an efficient and user-friendly method for UDG detection is essential.

Based on the findings presented above, it is proposed that the incorporation of specific DNA modifications into circular crRNA could inhibit the activity of CRISPR-Cas12a. This could be achieved by using a specific BER enzyme to selectively cleave the DNA modification-containing crRNA, restore its canonical conformation, and thereby reactivate CRISPR-Cas12a function. This would result in the digestion of the ssDNA fluorescent reporter (e.g., F-Q), allowing us to quantify BER enzyme levels based on the increase in fluorescence.

**Figure 4:**
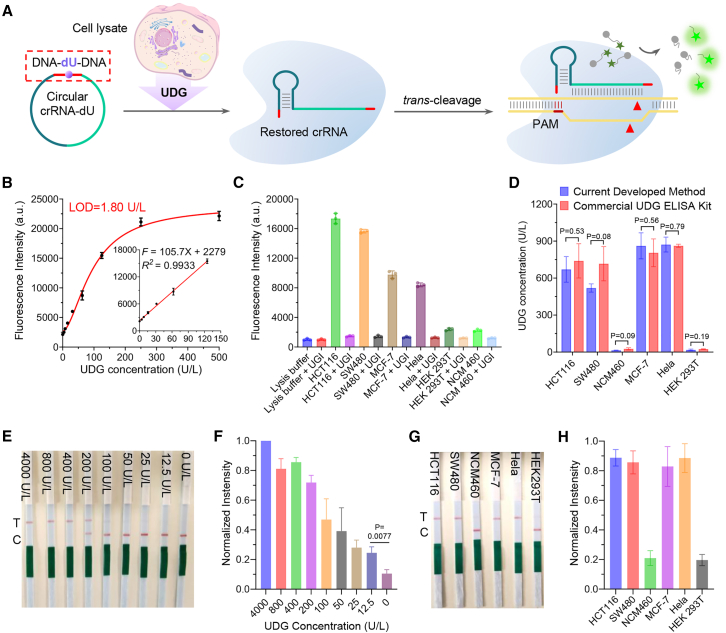
Development of a DNA modification-containing circular crRNA-assisted CRISPR-Cas12a system for BER enzyme detection and regulation of CRISPR-Cas12a gene editing by using BER enzymes (A) A conceptual diagram of the DNA modification-containing circular crRNA-assisted CRISPR-Cas12a system utilized for BER enzyme detection. BER enzymes can selectively act on specific DNA modifications, and circular crRNA can be opened to revert to its conventional form, thereby igniting LbCas12a *trans*-cleavage activity and releasing a fluorescent signal. This procedure is exemplified by UDG enzymes and dU. (B) Fluorescence data of detection of various UDG enzyme concentrations by this strategy, depicting the detection limit and quantitative relationship. (C) Comparison with and without UGI in different cell line protein extracts validates the uniqueness and effectiveness of UDG detection in the system. (D) Comparative bar graph for levels of UDG in different cell lines detected by current developed method and commercial UDG ELISA kit. (E) The paper strip assay was conducted to quantify the concentrations of UDG, and the results were documented using a mobile phone camera. (F) ImageJ software was used to analyze the grayscale bands in the images obtained from the paper strip assay for various concentrations of UDG. (G) The paper strip assay was employed for visual UDG detection of cell protein extracts from HCT116, SW480, NCM460, HeLa, and HEK293T cell lines. (H) ImageJ analysis was carried out on the grayscale bands from the visual detection results of the paper strip assay for the aforementioned cell lines. Error bars are mean ± SD (*n* = 3). Statistical significance was determined using a threshold of *p* < 0.05, where *p* > 0.05 indicates no significant difference and *p* < 0.05 denotes a statistically significant difference.

In this study, we selected dU as the model system for testing (Figure 4A). We designed a circular crRNA with a universal 19-nt DR region, an 18-nt RNA spacer targeting EBV, and a 7-nt DNA segment with dU strategically positioned in the center. Previous studies have shown that an 18-nt RNA spacer can greatly preserve the activity of CRISPR-Cas12a,^54^ and UDG has been found to efficiently recognize dU within a DNA chain of at least 7 bases.^77^ We performed cyclization of the precursor crRNA containing dU using T4 RNA ligase 1. Residual starting molecules and linear ligation by-products were removed using RNase R, generating a circular crRNA containing dU (Figures S6A and S6B). This dU-containing crRNA was then treated with UDG in a CRISPR-Cas12a working buffer containing spermidine. Our observations showed that UDG efficiently removes the uracil base, creating an AP site. With the assistance of spermidine, an addition reaction occurs at the AP site, followed by beta elimination, resulting in the generation of a beta-elimination product^72^ that reverts the circular crRNA back to its canonical form (Figures S6D and S6E).

Next, we developed a UDG detection platform using CRISPR-Cas12a, circular crRNA with dU targeting EBV, EBV target, and F-Q. Validation experiments confirmed that the dU-containing circular crRNA could suppress CRISPR-Cas12a activity, and the dU-specific BER enzyme UDG could selectively restore CRISPR-Cas12a activity by cleaving the dU-containing circular crRNA (Figures S6F–S6H). To further optimize the system, we investigated the influence of the initial concentration of the reaction substrate (EBV target) on the detection performance. Under consistent experimental conditions, we tested UDG detection limits at EBV target concentrations ranging from 40 nM, 20 nM, 10 nM, 5 nM, and 2.5 nM to 1.25 nM. Our results revealed that the detection limit for UDG remained stable at approximately 1.72–1.92 U/L, when the EBV target concentration was between 40 and 5 nM (Figures 3B and S7). However, when the EBV target concentration decreased to 2.5 nM, the detection limit deteriorated to 4.82 U/L (Figure S7D). Based on these findings, we selected 5 nM as the optimal initial concentration of the reaction substrate (EBV target) for our system. We then conducted UDG fluorescence detection experiments by systematically diluting UDG in the range of 0–500 U/L (Figures 4B and S8A). A strong linear relationship was observed between fluorescence and UDG concentration within the 0–125 U/L range (Figure 4B). Control experiments with bovine serum albumin, AP endonuclease 1 (APE1), human 8-oxoguanine DNA N-glycosylase 1 (hOGG1), UDG, and their mixtures (Figure S8B) demonstrated the specificity of the UDG detection method, with only UDG or the UDG-containing mixture opening the circular crRNA and restoring CRISPR-Cas12a activity, leading to increased fluorescence.

We also cultured various cancer cells (HCT116, SW480, MCF-7, HeLa), normal cells (human colon mucosal epithelial cells NCM460), and HEK293T to examine UDG concentrations. The UDG concentrations in MCF-7 and HeLa cells were found to be 850–880 U/L (Figure S8E), consistent with a previous report.^78^ The UDG concentrations in colorectal cancer cells (HCT116: 670.5 U/L, SW480: 519.6 U/L) were significantly higher than those in the non-cancer cell line HEK293T (17.9 U/L) and normal colon mucosal epithelial cells NCM460 (14.4 U/L) (Figure S8E). To validate our experiments, a commercial UDG ELISA kit was employed to evaluate the UDG content^78^ in the same cultured cells. The high level of consistency and comparability observed in the results serves as strong validation for the reliability of our methodology (Figures 4D and S8D–S8F). Additionally, uracil glycosylase inhibitor (UGI) was used as an inhibitor of UDG, and fluorescence detection in cell protein extracts was conducted with and without UGI. The addition of UGI resulted in a noticeable decrease in the fluorescence detection signal of UDG in cancer cells, bringing the fluorescence intensity of cancer cells with UGI to a level similar to that of HEK293T and NCM460 cells (Figure 4C). This secondary validation confirmed the high sensitivity of our UDG detection method. The half-maximal inhibitory concentration value for UGI in our method was calculated to be 0.087 U/mL (Figure S8C), consistent with the literature record of 0.07 U/mL.^78^ In addition to nuclear protein extracts, we also quantified UDG levels in lysates from diverse cell lines. Our findings revealed that the fluorescence intensity of UDG detection was significantly higher in HCT116 and SW480 cells compared to the non-cancerous cell line NCM460 (Figure S8G). These results provide strong validation of the effectiveness and success of our UDG detection method based on circular crRNA containing dU modifications.

To demonstrate the broad applicability and developmental value of this approach, we have enhanced the method and integrated it with a commercial test strip to develop a point-of-care testing (POCT) system for detecting BER enzymes (Figure S8H). This test strip is based on an immunochromatographic assay system, where the original F-Q has been replaced with an FAM-biotin DNA reporter (Figure S9H; Table S1), a DNA probe labeled with biotin at one end and FAM at the other end. In the absence of UDG, LbCas12a lacks *trans*-cleavage activity, leaving the FAM-biotin DNA reporter intact. When the strip is immersed in this solution, the intact reporter binds to colloidal gold conjugated with anti-FAM antibodies, migrating to the control (C) line and becoming fully captured by avidin, resulting in a visible band. In the presence of UDG, the circular RNA is opened, activating the *trans*-cleavage activity of LbCas12a, which cleaves the FAM-biotin DNA reporter. The cleaved DNA fragment with FAM but without biotin binds to the colloidal gold conjugated with anti-FAM antibodies, migrates to the C line, but is not captured by the avidin due to the absence of biotin, ultimately reaching the test (T) line where it is captured by immobilized anti-*anti*-FAM antibodies, resulting in a visible band at the T line. We tested this system using UDG solutions with known concentrations ranging from 0 to 4,000 U/L (Figure 4E). The test strip results enabled us to detect UDG concentrations as low as 12.5 U/L (Figure 4F). As the UDG concentration increased, the signal intensity at the T line strengthened, while that at the C line weakened. By analyzing the grayscale values of the test strip images via ImageJ, we were able to achieve a rough quantification of UDG levels (Figure 4F). To evaluate the reliability of our method, we tested protein extracts from different cell lines. Notably, cell lines with high UDG expression such as HCT116, SW480, MCF-7, and HeLa effectively activated the LbCas12a system, displaying stronger visible bands on the T line. In contrast, cell lines with low UDG expression like NCM460 and HEK293T exhibited weaker bands on the T line (Figures 4G and 4H). This innovative and visual detection method is quickly responsive and reliable in various environments, successfully eliminating the need for complex instrumentation and simplifying the existing detection process, thus providing an efficient pathway for high-performance POCT.

To demonstrate the programmability and versatility of our design, we developed a circular crRNA containing tetrahydrofuran (THF; Figure S9H), a modification, for the detection of APE1. APE1 is known to be highly selective for breaking THF.^79^ We designed a circular crRNA with a universal 19-nt DR region, an 18-nt RNA spacer, and 5-nt DNA bases with THF incorporated (Figure S8I). The circular crRNA containing THF was synthesized and confirmed (Figure S6C). We also demonstrated that APE1 could efficiently open the circular crRNA containing THF, and the crRNA with THF could be used to detect APE1 levels (Figures S8J–S8L). This highlights the potential of our design paradigm to be extended to other modified bases and enzymes, such as 8-oxo-guanine and hOGG1.^67^^,^^68^

### Regulation of CRISPR-Cas12a cleavage activity through the use of BER enzyme corresponding to DNA modification

**Figure 5:**
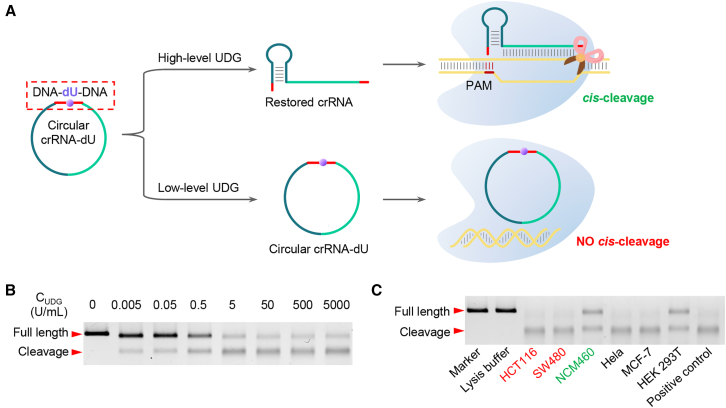
Precise regulation of CRISPR-Cas12a *cis*-cleavage through the use of BER enzymes corresponding to DNA modifications on circular crRNA (A) Schematic representation of the regulation of CRISPR-Cas12a *cis*-cleavage through different concentrations of UDG. (B) A 2% agarose gel showing variable UDG concentrations (from 0 to 5,000 U/mL) markedly affects the gene editing capabilities of this dU-containing circular crRNA-assisted CRISPR-Cas12a system. (C) *In vitro* regulation of this system’s *cis*-cleavage abilities by protein extracts from different cell lines. Relative to normal human colonic epithelial cells, highlighted in green, this system possesses stronger *cis*-cleavage abilities in high UDG expression colon cancer cell line protein extracts, highlighted in red.

Based on the recognition ability of BER enzymes for DNA modifications and the abundance of certain BER enzymes in cancer cells, we proposed the development of a regulatory system for CRISPR-Cas12a *cis*-cleavage activity using BER enzymes. Initial testing was performed using crRNA containing dU, as shown in Figure 5A. In systems with high UDG expression, the circular crRNA containing dU was broken down due to UDG treatment, resulting in a reduction of the inhibitory effect on LbCas12a and an enhancement of its *cis*-cleavage capability. Conversely, in systems with low or no UDG expression, the circular crRNA containing dU remained intact, leading to the inhibition of the *cis*-cleavage capacity of LbCas12a.

To examine UDG’s effect on the *cis*-cleavage of CRISPR-Cas12a, we tested its EBV-targeting activity at varying concentrations. As shown in Figure 5B, CRISPR-Cas12a showed no *cis*-cleavage without UDG, but activity increased progressively with higher UDG levels. Subsequently, we extracted proteins from both colorectal cancer cell lines HCT116 and SW480, known for high UDG expression, and from HEK293T and human colon mucosal epithelial cells NCM460 known for low UDG expression (Figure 4D). Through *in vitro* verification, we affirmed that extracted protein from HCT116 and SW480 (colorectal cancer cells) gave the developed CRISPR-Cas12a system a notably stronger *cis*-cleavage ability (Figure 5C). On the contrary, protein extracts from NCM460 (a normal human colon mucosal epithelial cell line) resulted in a comparatively low *cis*-cleavage performance (Figure 5C). This assay verified the feasibility of our approach to regulate LbCas12a *cis*-cleavage through the use of BER enzymes acting on DNA modification-containing circular crRNA. This study contributes to the development of more precise and controlled *cis*-cleavage techniques. By utilizing BER enzymes in the regulation of LbCas12a *cis*-cleavage, we created a potential strategy for enhancing editing precision and specificity. Future endeavors will focus on more in-depth studies in cellular and animal models. Our design, based on the DNA modification-containing circular crRNA-assisted CRISPR-Cas12a system, holds great promise for future gene therapy applications, particularly in cell types exhibiting differential BER enzyme levels.

## Discussion

The CRISPR-Cas12a system has emerged as a transformative technology in medical nucleic acid detection, offering remarkable clinical utility in emergency scenarios and disease outbreaks.^20^^,^^39^ Its advantages include minimal sample requirements, isothermal operation at 37°C within 30–45 min, and independence from specialized equipment or personnel.^21^^,^^34^ However, challenges persist in integrating isothermal amplification with CRISPR-Cas12a for trace nucleic acid detection, particularly in one-pot assays, alongside difficulties in precise Cas12a regulation and the system’s current limitation to nucleic acid targets.^38^

In this study, we systematically evaluated various engineered circular RNA, including full-length and split systems, for their ability to modulate LbCas12a activity. Our approach introduces a universal circular DR with a PC linker, compatible with diverse spacers for different target detection, significantly reducing cost and complexity while enhancing versatility. Compared to linear RNAs, our circular RNAs demonstrated superior stability, maintaining integrity after 60-min incubation in 10% FBS or 24-h exposure to lab routine light, underscoring their robust practical utility (Figures S1D–S1G and S4C–S4F). Furthermore, our circularization strategy extends beyond nucleic acid detection, enabling non-nucleic acid target detection through introducing DNA modifications on circular RNA, thereby expanding potential applications in gene therapy and enzyme detection.

A key innovation involves integrating circular RNA design with split crRNA systems. While split crRNAs showed slightly higher detection limits (1.17 pM; Figures S9C and S9D) compared to full-length crRNA (0.10 pM; Figures S9A and S9B) in stand-alone CRISPR assays, performance converged in one-pot RPA-CRISPR assays (12.72 vs. 14.04 copies/mL; Figures 5B and S3B). This performance parity can be attributed to the temporal inhibition of Cas protein activity by circular crRNA or DR during isothermal amplification, which facilitates effective target amplification even at low initial concentrations. Consequently, in the RPA-CRISPR one-pot system, the detection limit becomes less dependent on the efficiency of the CRISPR system, whether using full-length or split constructs. These findings align with recent work by Cheng et al., which demonstrated that heparin sodium reduces Cas12a efficiency while enhancing low-concentration target detection in one-pot assays, published during our manuscript review period.^80^ Our universal circular DR design, requiring only a single synthesis, holds promise for application to diverse targets.

Notably, our optimized system demonstrated minimal *trans*-cleavage activity with spacer alone (100 nM; Figure S9E), remaining negligible compared to DR + spacer complexes. Even at 5 μM, no significant spacer-induced *cis*-cleavage was observed (Figure S9F), aligning with observations by Shebanova et al.,^62^ reinforcing the crucial role of DR + spacer complexes in driving LbCas12a activity. While our current focus is on developing universal circular DR, we acknowledge the potential of spacer circularization for precise Cas protein activity control, with circular DR + circular spacer combinations showing particularly low background signals (Figure S9G).

We further introduced DNA modifications in circular crRNAs, effectively inhibiting LbCas12a activity while enabling non-nucleic acid target detection. In UDG detection, our method outperformed commercial ELISA kits, offering superior specificity by eliminating antibody dependence and associated cross-reactivity risks. The streamlined protocol, leveraging UDG’s inherent specificity for dU in circular crRNA, enables rapid detection with minimal sample preparation, significantly reducing time and cost. This modular platform technology allows for the detection of various DNA modification-related enzymes through simple target-specific DNA modification substitutions, contrasting with ELISA’s requirement for target-specific antibody development.

Additionally, we propose a potential precise regulation of the CRISPR-Cas12a *cis*-cleavage strategy based on the expression of BER enzymes. Our *in vitro* experiments have revealed that higher expression of BER enzymes, such as UDG, in colorectal cancer cell lines leads to enhanced *cis*-cleavage activity compared to normal colon mucosal epithelial cell line with lower UDG expression. Given the varying expression levels of enzymes in specific cells, this design, which is matched one-to-one with DNA modifications and BER enzymes, holds potential for future clinical applications in gene therapy. These findings provide perspectives and opportunities for clinical nucleic acid detection and future gene editing.

In summary, this study demonstrates improved regulation of the CRISPR-Cas12a system through circular RNA designs and establishes a platform for BER enzyme detection. Using universal circular DR with a PC linker, we developed a versatile, simple, and cost-effective trace nucleic acid detection system compatible with isothermal amplification. The introduction of DNA modification on circular crRNAs enables BER enzyme detection and shows potential for future gene therapy applications. These findings contribute to clinical diagnostics and gene editing technologies.

### Limitations of the study

The light-triggerable circular RNA system is dependent on external UV light activation, which may restrict its applicability in resource-limited settings and necessitates additional UV light-protection measures and operator safety protocols. While DNA modifications on circular crRNA facilitate CRISPR-Cas12a regulation through BER enzymes, and this mechanism has been validated *in vitro*, the potential impact of cell-to-cell variability in BER enzyme expression on *in vivo* gene editing efficacy remains unexplored. Commercially available UDG exhibited greater DNA cleavage variability in *in vitro* assays compared to endogenously expressed UDG in cellular extracts, highlighting the significant challenges this strategy may encounter within the complex intracellular environment. Insufficiently distinct BER enzyme expression levels may compromise the system’s regulatory efficacy. Although RNA circularization enhances the regulation of Cas12a activity and expands its utility for non-nucleic acid targets, the process of RNA circularization itself presents technical and cost-related challenges. Advances in RNA circularization technology are anticipated to address these limitations in the future.

## Resource availability

### Lead contact

Further information and requests for resources and reagents should be directed to and will be fulfilled by the lead contact, Chaoxing Liu (liuchx69@mail.sysu.edu.cn).

### Materials availability

This study did not generate new unique reagents.

### Data and code availability

• The uncropped raw gel images associated with this study have been deposited in Figshare and are publicly accessible under the following weblink: https://doi.org/10.6084/m9.figshare.28887740.
• This paper does not report original code.
• Any additional information required to reanalyze the data reported in this paper is available from the lead contact upon request.

## Acknowledgments

This work was supported by the 10.13039/501100001809National Natural Science Foundation of China (grant no. 22307150), the Shenzhen Medical Research Fund (grant nos. A2303012 and A2401010), the 10.13039/501100021171Guangdong Basic and Applied Basic Research Foundation (grant no. 2024A1515012319), and the Shenzhen Science and Technology Program (grant nos. JCYJ20230807110315032 and JCYJ20240813150427036). Human blood samples were collected and provided by the Seventh Affiliated Hospital, Sun Yat-sen University, with protocols approved by the ethics committee at the Seventh Affiliated Hospital, Sun Yat-sen University (KY-2024-009-02 and KY-2023-117-01).

## Author contributions

C.L., J.W., and W.Z. contributed to conceptualization, supervision, and study design. J.W., W.Z., W.L., Q.X., and Z.Z. performed the experiments and data analysis. C.L., J.W., and W.Z. verified the underlying data and wrote the manuscript. All authors reviewed, revised, and approved the final version of the manuscript.

## Declaration of interests

The authors declare no competing interests.

## STAR★Methods

### Key resources table

**Table:** 

REAGENT or RESOURCE	SOURCE	IDENTIFIER
**Biological samples**		

Clinical human blood samples	The Seventh Affiliated Hospital, Sun Yat-sen University	N/A

**Chemicals, peptides, and recombinant proteins**		

LbCas12a protein	Hylegen Bioscience	Cat#CAS12-010B
EnGen Lba Cas12a (Cpf1)	New England Biolabs	Cat#M0653T
T4 RNA Ligase 1	New England Biolabs	Cat#M0204L
Uracil-DNA Glycosylase (UDG)	New England Biolabs	Cat#M0280L
APE1	New England Biolabs	Cat#M0282S
TranslTX2 Dynamic Delivery System MlR 6000	Mirus bio	Cat#MIR 6000
RNase R	Yeasen Biotechnology	Cat#14606ES72
Dulbecco's Modified Eagle Medium	Gibco	Cat#11995065
Fetal Bovine Serum	Yeasen Biotechnology	Cat#40130ES76
1% penicillin-streptomycin	Bioleaper	Cat#BR3001460
Roswell Park Memorial Institute 1640 medium	Gibco	Cat#11875119
PBS	Biosharp	Cat#BL302A
T7 Endonuclease I	Vazyme Biotech	Cat#EN303-02
VAHTS DNA Clean Beads	Vazyme Biotech	Cat#N411-02
VAHTS RNA Clean Beads	Vazyme Biotech	Cat#N412-02
DNA Dye SuperRed	Biosharp	Cat#BS354B
Opti-MEM I	Thermo Fisher Scientific	Cat#31985070
DNA Dye SuperRed	Biosharp	Cat#BS354B
Stabilized dNTP Mix	GenScript Biotech Corporation	Cat#C01689
dUTP (100 mM)	GenScript Biotech Corporation	Cat#C01717
T7 RNA Polymerase	GenScript Biotech Corporation	Cat#E00066
RIPA Lysis Buffer II	Sangon Biotech	Cat#C510006
ApexHF HS DNA Polymerase FS	Accurate Biotechnology	Cat#AG12201
Hieff Canace Plus High-Fidelity DNA Polymerase	Yeasen Biotechnology	Cat#10153ES76

**Critical commercial assays**		

RT-RPA 2.0	Keer Life	Cat#901221021
EpiTect Bisulfite Kit	Qiagen	Cat#59104
MolPure Cell/Tissue DNA Kit	Yeasen Biotechnology	Cat#18700ES70
QIAamp DNA Blood Mini Kit	Qiagen	Cat#51183
MolPure PCR Purification Kit	Yeasen Biotechnology	Cat#19106ES50
Enhanced Cell Counting Kit 8 (WST-8/CCK8)	Elabscience Biotechnology	Cat#E-CK-A362
Lateral flow detection strips	Keer Life	Cat#CAS-cmCSA01
Ready to use 2XTaq Master Mix	Generary Biotechnology	Cat#GK8005
DNA Damage Comet Assay Kit	GBCBIO Technologies	Cat#G7668
Human Uracil-DNA Glycosylase (UNG) ELISA Kit	Ziker	Cat#ZK-H2551

**Deposited data**		

The uncropped raw gel images.xlsx	Figshare	https://doi.org/10.6084/m9.figshare.28887740

**Experimental models: Cell lines**		

Human cell line HCT116	China Center for Type Culture Collection	Cat#GDC0625
Human cell line SW480	China Center for Type Culture Collection	Cat#GDC0306
Human cell line NCM460	Wuhan Sunncell Biotechnology Co., Ltd	Cat#SNL-519
Human cell line Hela	China Center for Type Culture Collection	Cat#GDC0009
Human cell line MCF-7	China Center for Type Culture Collection	Cat#GDC0055
Human cell line HEK 293T	Wuhan Sunncell Biotechnology Co., Ltd	Cat#SNL-015

**Oligonucleotides**		

Oligonucleotides used in this work	This paper	See Table S1

**Software and algorithms**		

Prism v9.5.1	GraphPad Software LLC.	https://www.graphpad.com/updates/prism-951-release-notes
Image Lab v5.1	Bio-Rad Laboratories, Inc.	https://www.bio-rad.com/en-cn/product/image-lab-software?ID=KRE6P5E8Z
ImageJ v1.46r	National Institutes of Health (NIH)	https://imagej.net/ij/docs/guide/146.html

**Other**		

Celemetor Real-Time Fluorescent Quantitative PCR Analysis System	Yeasen Biotechnology Co., Ltd.	Celemetor 96
CFX96 Touch Real-Time PCR Detection System	Bio-Rad Laboratories, Inc.	CFX96 Touch
ChemiDoc MP Imaging System	Bio-Rad Laboratories, Inc.	ChemiDoc MP
Leica DM6B Upright Microscopes	Leica	DM6B

### Experimental model and study participant details

#### Cell culture

The HCT116 and SW480 cell lines were obtained from the China Center for Type Culture Collection (CCTCC, Wuhan, China). The NCM460 cell line was purchased from Wuhan Sunncell Biotechnology Co., Ltd (Wuhan, China). All cell lines were authenticated using STR identification and confirmed to be free of contamination. All cell lines were confirmed to be free of Mycoplasma contamination. Culturing of HCT116, SW480, HeLa, MCF-7, and HEK293T cells was performed in Dulbecco's Modified Eagle Medium (DMEM) (Gibco), supplemented with 1% penicillin-streptomycin (Bioleaper) and 10% fetal bovine serum (FBS) (YEASEN), at 37°C, with 5% CO_2_. For NCM460 cell culture, Roswell Park Memorial Institute 1640 medium (RPMI-1640) (Gibco) complemented with 10% FBS was used under similar conditions as listed above.

#### Patient material & consent

Human blood samples were collected and provided by the Seventh Affiliated Hospital, Sun Yat-sen University with protocols approved by the ethics committee at the Seventh Affiliated Hospital, Sun Yat-sen University (KY-2024-009-02 and KY-2023-117-01). All patients in this study signed an informed consent form. 25 blood samples from EBV-infected patients and 20 samples from healthy donors were collected. Using the QIAamp DNA Blood Mini Kit (Qiagen) extracted all clinical human blood samples DNA following the manufacturer’s protocol. And the final extracted DNA products were stored at −80°C for further use.

### Method details

#### Preparation and validation of circular RNA

To obtain circular RNA, we mixed a final concentration of 1X Reaction Buffer (50 mM Tris-HCl, pH 7.5, 10 mM MgCl_2_, 1 mM DTT), 0.5 μM Circular RNA precursor with 5’-phosphoric acid and 3’-hydroxy termini (EBV-Cir-crRNA precursor, Circular DR precursor, Circular-crRNA-dU precursor or Circular-crRNA-THF precursor, Table S1), 1 unit of T4 RNA Ligase 1, 10% (v/v) PEG 8000, 50 μM ATP, and nuclease-free water to a final volume of 20 μL. The reaction mixture was then incubated at 37°C for 1 hour, followed by inactivation of the ligase at 95°C for 2 minutes. Subsequently, 4 units of RNase R were added, and the mixture was further incubated at 37°C for 30 minutes to digest non-circular RNA. The remaining product was heated at 70°C for 10 minutes to inactivate RNase R, followed by PAGE purification for storage at -20°C. The circularization efficiency was validated by performing denaturing 18% PAGE with 7 M urea.

#### Determining the cleavage time of the PC linker in circular RNA

To determine the time required for the breakage of the PC linker in Circular EBV-Cir-crRNA or Circular DR under UV light exposure (λ= 365 nm, 35 W), we prepared a mixture containing a final concentration of 1X LbCas12a Reaction Buffer (10 mM Tris-HCl, pH 8.5, 10 mM MgCl_2_, 20 mM KCl, 1 mM DTT, 40 mM glycine, 0.01% (v/v) Tween 20), 1 μM Circular EBV-Cir-crRNA or 2 μM Circular DR and nuclease-free water to achieve a final volume of 10 μL. Subsequently, the mixture was subjected to UV light exposure for 0 s, 10 s, 30 s, 60 s, 600 s and 1200 s. To evaluate the breakage of the PC linker after UV light exposure, the Circular EBV-Cir-crRNA or Circular DR samples were analyzed using denaturing 18% PAGE with 7 M urea after GelstainRed Nucleic Acid Dye staining.

#### Photocontrolled AAVS1-Cir-crRNA assisted CRISPR-Cas12a gene editing experiment

HEK 293T cells were seeded at a density of 1x10^5^ cells per well in a 24-well plate. The cells were cultured in Dulbecco's Modified Eagle Medium (DMEM) supplemented with 10% Fetal Bovine Serum and 1% penicillin/streptomycin. The culture conditions were maintained at 37°C in a 5% CO_2_ atmosphere for 20 hours until the cells reached approximately 80% confluence. After that, 250 pmol of either AAVS1 crRNA or AAVS1-Cir-crRNA was mixed with 50 μL of Opti-MEM I and gently mixed. Subsequently, 100 pmol of EnGen Lba LbCas12a (Cpf1) was added to the mixture, followed by gentle mixing and incubation at room temperature for 15 minutes. Next, 2 μL of the TransIT-X2 Dynamic Delivery System was added to the transfection mixture, gently mixed again, and incubated at room temperature for an additional 15 minutes. The entire mixture was then added to the cells in the 24-well plate and gently shaken to ensure even distribution. After 6 hours of incubation, the cells were exposed to ultraviolet light (λ = 365 nm, 35 W) for either 3 minutes or left unexposed. The cells were then incubated for an additional 42 hours at 37°C in a 5% CO_2_ atmosphere.

#### T7 endonuclease I (T7EI) assay for evaluating gene editing efficiency

Genomic DNA was extracted from HEK 293T cells 48 hours post-transfection using the MolPure Cell/Tissue DNA Kit. After that, a PCR mixture containing 5 pmol of AAVS1-PCR-F and AAVS1-PCR-R primers, 100 ng of extracted HEK 293T genomic DNA, 1×Canace Plus PCR buffer (with Mg^2+^ and dNTPs), and 1 unit of Hieff Canace Plus High-Fidelity DNA Polymerase was prepared and brought to a final volume of 50 μL with nuclease-free water. The PCR conditions were as follows: initial denaturation at 98°C for 8 minutes, followed by 40 cycles of 98°C for 10 seconds, 60°C for 15 seconds, and 72°C for 30 seconds. A final extension was performed at 72°C for 5 minutes. The PCR products were then purified using the MolPure PCR Purification Kit. Next, A 200 ng aliquot of the purified PCR product was mixed with 1×T7EI Reaction Buffer, and nuclease-free water was added to a final volume of 19 μL. The mixture was subjected to the following annealing protocol: 95°C for 5 minutes, followed by a gradual cooling to 85°C at ∼2°C/second and further cooling to 25°C at ∼0.1°C/second. After annealing, 1 μL of T7EI was added, and the reaction was incubated at 37°C for 20 minutes. The digestion products were immediately analyzed by 2% agarose gel electrophoresis. T7EI specifically cleaves DNA duplexes at mismatched base pairs, generating distinct fragment patterns. In this assay, T7EI targets mutation sites introduced by gene editing, producing fragments of varying lengths compared to the wild-type. The cleavage efficiency, which reflects the percentage of insertions and deletions (Indels), is quantified by analyzing gel electrophoresis images using ImageJ software. This is achieved by calculating the ratio of the intensities of cleaved bands to the total band intensities.

#### Traditional RPA-Cas12a one-pot nucleic acid detection for EBV

The reaction mixture was prepared in a final volume of 20 μL, containing the following components: 1× LbCas12a Reaction Buffer, 100 nM LbCas12a, 1 nM EBV-specific crRNA, 250 nM F-Q, and 9 μL of RT-RPA 2.0 master mix (each lyophilized pellet dissolved in a total of 25 μL ddH2O). Additionally, 0.1 μM of each primer (EBV-RPA-F and EBV-RPA-R; Table S1) was included, along with varying concentrations of EBV target DNA. Nuclease-free water was used to adjust the total reaction volume to 20 μL. All reagents were thoroughly mixed in qPCR-compatible tubes and incubated at 37°C for 45 minutes in a qPCR instrument. Fluorescence signals were monitored in real-time, and the EBV content in clinical samples was quantified by extrapolating the fluorescence intensity against a pre-established standard curve.

#### Photocontrolled one-pot method for Clinical EBV sample analysis using EBV-Cir-crRNA or circular DR

For EBV-Cir-crRNA assisted Photocontrolled one-pot Method, we prepared a final concentration of 1X LbCas12a RPA Reaction Buffer (2 mM Tris-HCl, pH 8.5, 28 mM MgCl_2_, 4 mM KCl, 0.2 mM DTT, 8 mM glycine, 0.002 % (v/v) Tween 20), 100 nM LbCas12a, 1 nM EBV-Cir-crRNA, 250 nM F-Q, 9 μL RT-RPA 2.0 Mix (each lyophilized pellet dissolved in a total of 25 μL ddH_2_O), and 0.1 μM of each primer (EBV-RPA-F and EBV-RPA-R, Table S1). We also added 2 μL of either a template made with varying copy numbers of EBV Target described above or a clinical sample to adjust the final volume to 20 μL.

For Circular DR assisted one, the similar conditions are used, we prepared a final concentration of 1X Circular DR-LbCas12a RPA Reaction Buffer (2 mM Tris-HCl, pH 8.5, 28 mM MgCl_2_, 4 mM KCl, 0.2 mM DTT, 8 mM glycine, 0.002% (v/v) Tween 20, 1 mM ADP, 10 units of T4 Polynucleotide kinase), 100 nM LbCas12a, 100 nM Circular DR, 100 nM EBV Spacer, and 250 nM F-Q, 9 μL RT-RPA 2.0 mix, 0.1 μM of each primer (EBV-RPA-F and EBV-RPA-R). All reagents are mixed in qPCR tubes and placed into the qPCR instrument at 37°C for an RPA amplification of 15 minutes (monitoring fluorescence every 5 minutes). After amplification, the tubes are placed under UV light (λ= 365 nm, 35 W) for 30 seconds before returning to the qPCR instrument for continued fluorescence collection at 37°C for 60 cycles, lasting for 30 minutes. The EBV content in the clinical samples is calculated based on standard curves.

#### Detection of different UDG enzyme levels in various cell lines using circular-crRNA-dU assisted CRISPR-Cas12 system

Firstly, we prepared a reaction mixture with a final concentration of 0.5 nM Circular-crRNA-dU, 1X LbCas12a-UDG Reaction Buffer (10 mM Tris-HCl, pH 8.5, 10 mM MgCl_2_, 20 mM KCl, 1 mM DTT, 40 mM glycine, 1 mM spermidine, 0.01% (v/v) Tween 20). Next, we added 1 μL of UDG diluted with LbCas12a-UDG Reaction Buffer to different final concentrations (500, 250, 125, 62.5, 31.25, 15.63, 7.81, 3.91 U/L), or 1 μL of cell nuclear protein extract diluted with LbCas12a-UDG Reaction Buffer (HCT116, SW480, HeLa, MCF-7 extract diluted 10 times, NCM460, HEK293T extract diluted 2 times). The reaction mixture was brought to a total volume of 10 μL with nuclease-free water, followed by incubation at 37°C for 20 minutes. Finally, we added a final concentration of 100 nM LbCas12a, 250 nM F-Q, and 5 nM EBV Target, and brought the volume to 20 μL with nuclease-free water. The reaction mixture was promptly placed in a qPCR instrument, and fluorescence was collected every 30 seconds at 37°C for a total of 60 cycles, lasting 30 minutes. The UDG enzyme levels in the cell nuclear protein extracts were determined using the standard curve generated with different concentrations of commercial UDG standard (NEB, Cat. No. M0280L).

#### Detection of nuclear UDG enzyme activity in various cell lines using lateral flow assay strips

A reaction mixture was prepared containing 0.5 nM Circular-crRNA-dU in LbCas12a-UDG Reaction Buffer (10 mM Tris-HCl, pH 8.5, 10 mM MgCl2, 20 mM KCl, 1 mM DTT, 40 mM glycine, 1 mM spermidine, 0.01% (v/v) Tween 20). For the standard curve, 3 μL of UDG at various concentrations (4000, 800, 400, 200, 100, 50, 25, 12.5, 0 U/L) was added. For sample analysis, 3 μL of nuclear protein extract from HCT116, SW480, NCM460, MCF-7, HeLa, and HEK 293T cells was added. The total volume was adjusted to 60 μL with nuclease-free water, and the mixture was incubated at 37°C for 20 minutes. Subsequently, 100 nM LbCas12a, 250 nM F-Q, and 5 nM EBV Target were added to the reaction, and the volume was adjusted to 20 μL with nuclease-free water. After incubation at 37°C for 20 minutes, a lateral flow assay strip was inserted into the reaction tube and allowed to react for 8 minutes. The results were observed and photographed, followed by analysis using ImageJ software.

### Quantification and statistical analysis

#### Statistical analysis and data interpretation

All experimental data were analyzed using GraphPad Prism software (version 9.5.1). Results are presented as the mean ± standard deviation (SD) from three independent replicates, unless otherwise specified. Nonlinear regression was performed using the Sigmoidal, 4PL (four-parameter logistic) model with concentration (X) as the independent variable, while linear regression was conducted using the Simple Linear Regression tool. Statistical comparisons were performed using unpaired two-tailed t-tests, with p > 0.05 considered not significant and p < 0.05 deemed statistically significant. For real-time fluorescence curve analysis, the endpoint fluorescence intensity was used for bar graph representations unless otherwise noted. The time points for establishing the linear relationship between target concentration and fluorescence intensity were selected based on the following criteria: the fluorescence curve must remain in the linear phase without reaching the plateau, the fluorescence intensity at the chosen time point should exhibit significant differences across the target concentration gradient, and the intensity must demonstrate a strong linear correlation with target concentration, as evidenced by an R^2^ value greater than 0.9. This approach ensures robust and reproducible quantification of target analytes.

## Supplemental information

Document S1. Figures S1–S9 and Table S1

Document S2. Article plus supplemental information
