# Supplementary material for: Enhancement of CRISPR-Cas12a system through universal circular RNA design
Source: Cell Rep Methods. 2025 Jun 16;5(6):101076. doi: 10.1016/j.crmeth.2025.101076 (PMC12272218; doi:10.1016/j.crmeth.2025.101076)
Supplement: Document S1. Figures S1–S9 and Table S1 [file mmc1.pdf]

Cell Reports Methods, Volume *5*

Supplemental information

Enhancement of CRISPR-Cas12a system through universal circular RNA design

Jiaqi Wang, Wei Zhang, Wentao Li, Qinyuan Xie, Ziyu Zang, and Chaoxing Liu

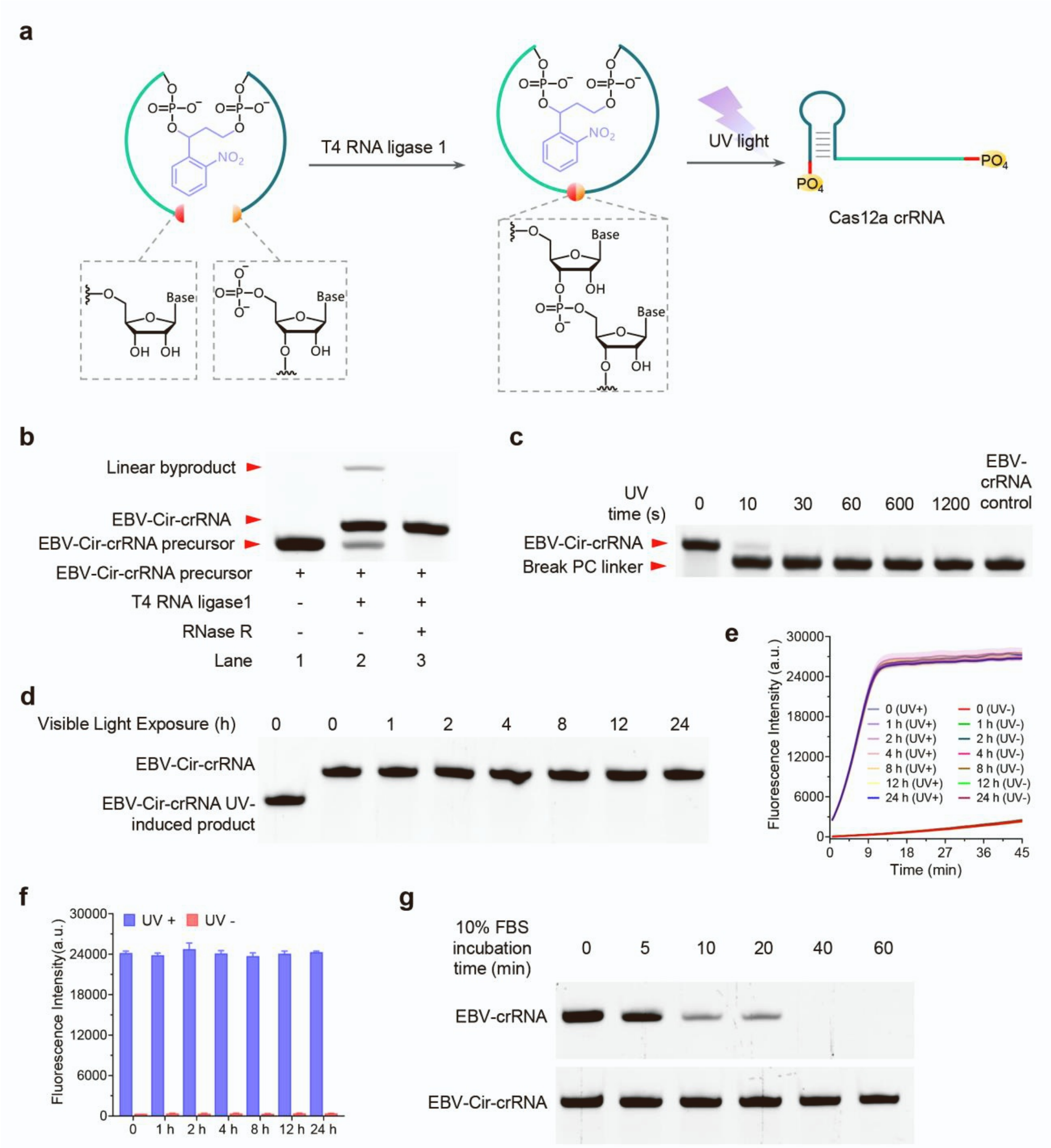

Figure S1: Preparation and verification of circular RNA, related to Figures 1, 2. (a) A schematic illustration of the creation of circular crRNA and the subsequent disruption at the PC linker of the circular crRNA under UV light, reverting to its classic configuration. (b) PAGE analysis of EBV-Cir-crRNA precursor producing a circular product under the action of T4 RNA Ligase1. Subsequently, RNase R is used to remove all linear molecules, including intermolecular ligated products and unreacted starting materials. (c) PAGE analysis of the condition of the PC linker of EBV-Cir-crRNA after UV radiation

(365 nm, 35w) for 0-1200s. From the results, it can be seen that upon 30s of UV exposure, EBV-Cir-crRNA completely breaks and reverts to the non-circular state and no significant changes occur even up to 20 minutes. (d) Structural stability assessment of EBV-Cir-crRNA under normal laboratory lighting. 100 ng of EBV-Cir-crRNA was incubated in transparent 0.2 mL microcentrifuge tubes on ice for 0, 1, 2, 4, 8, 12, and 24 hours, separately. For comparison, 100 ng of UV-induced EBV-Cir-crRNA cleavage product was used as a positive control for PC linker disruption. All samples were analyzed by 12% denaturing PAGE (150 V, 30 min) to evaluate PC linker integrity. The results demonstrated the robust structural stability of circular RNA under standard laboratory conditions. (e) Real-time fluorescence monitoring of LbCas12a *trans*-cleavage activity assays comparing EBV-Cir-crRNA exposed to standard laboratory lighting versus unexposed controls over a 24-hour period. No significant variation in LbCas12a activation was observed, confirming the photostability of the circular RNA design. (f) Quantitative analysis of fluorescence intensity at the 45-minute time point from panel (e), presented as a bar graph for direct comparison. (g) Enhanced environmental stability of EBV-Cir-crRNA compared to linear EBV crRNA. 100 ng of each RNA was incubated with 2 μL of Dulbecco's Modified Eagle Medium (DMEM) supplemented with 10% fetal bovine serum (FBS) at 37 °C for 0, 5, 10, 20, 40, and 60 minutes, separately. Analysis by 12% denaturing PAGE (150 V, 30 min) revealed that linear crRNA exhibited significant degradation within 10 minutes and was completely degraded by 40 minutes, whereas EBV-Cir-crRNA maintained its structural integrity throughout the 60-minute incubation period. Error bars represent the mean ± standard deviation (S.D.) of triplicate measurements, ensuring statistical reliability.

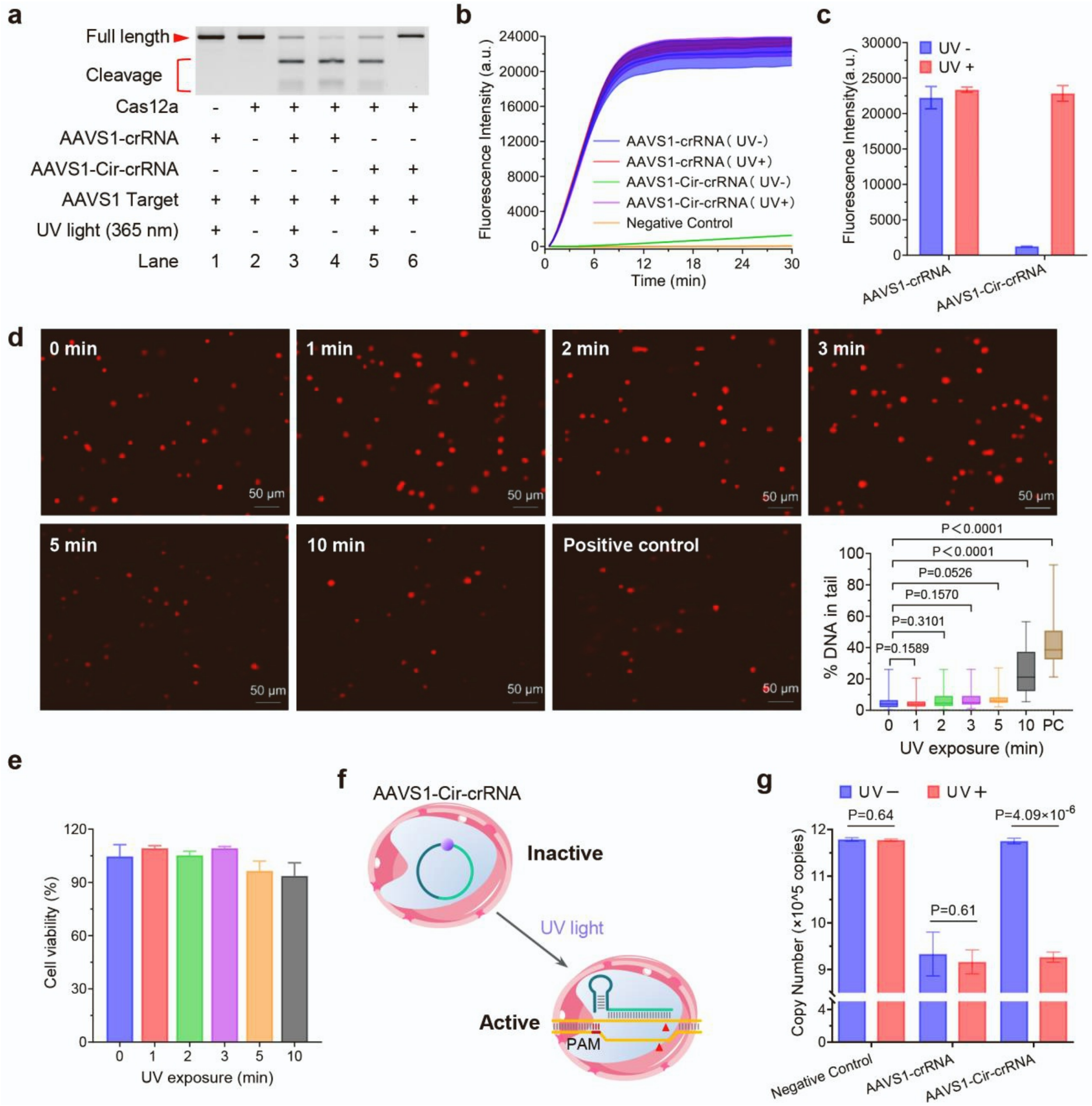

Figure S2: Functional characterization of circular AAVS1-Cir-crRNA with a PC linker in CRISPR-Cas12a activity and UV-induced effects, related to Figure 2. (a) Analysis on a 2% agarose gel confirms that circular AAVS1-Cir-crRNA with a PC linker effectively inhibits Cas12a *cis*-cleavage activity towards the AAVS1 Target. Notably, half a minute under UV light can restore this activity. (b) Investigation of circularized crRNA targeting the AAVS1 locus with a PC linker. Fluorescence release from the corresponding DNA target was measured in vitro, induced by LbCas12a *trans*-cleavage activity under two conditions: with and without 30-second UV irradiation. The results demonstrate that circularized crRNA effectively inhibits LbCas12a activity, while UV irradiation for 30 seconds restores

its function. (c) Fluorescence intensity data of (b) at the 30-minute time point are compared under two conditions: with and without UV light exposure. (d) Genome-wide assessment of UV-induced DNA damage. HEK293T cells were exposed to UV light (λ = 365 nm) for 1, 2, 3, 5, and 10 minutes with the lamp 15 cm above culture dishes on ice. Untreated cells (negative control) and cells treated with 50 μM H_2O_2 for 1 hour (positive control) were used. Post-exposure, cells were trypsinized, washed twice with PBS, and resuspended at 1×10^6 cells/mL. The comet assay was performed using a DNA Damage Comet Assay Kit (GBCBIO Technologies, Cat. No. G7668) following the manufacturer's protocol. Images were acquired via fluorescence microscopy and analyzed using the OpenComet plugin in ImageJ. (e) UV irradiation effects on cell viability. HEK293T cells were exposed to UV light (λ = 365 nm) for 0, 1, 2, 3, 5, and 10 minutes. Viability remained stable for 0-3 minutes but showed a slight decline after 5 minutes. (f) Schematic showing the influence of circular AAVS1-Cir-crRNA with a PC linker assisted CRISPR-Cas12a system's gene editing at the cellular level with or without UV light exposure. (g) A bar chart depicts the unedited AAV1 gene copy number in AAVS1 crRNA, AAVS1-Cir-crRNA assisted CRISPR-Cas12a system's gene editing at the cellular level with and without UV light exposure. All experiments were performed in triplicate; error bars represent mean ± S.D., $P > 0.05$ indicates no statistically significant difference.

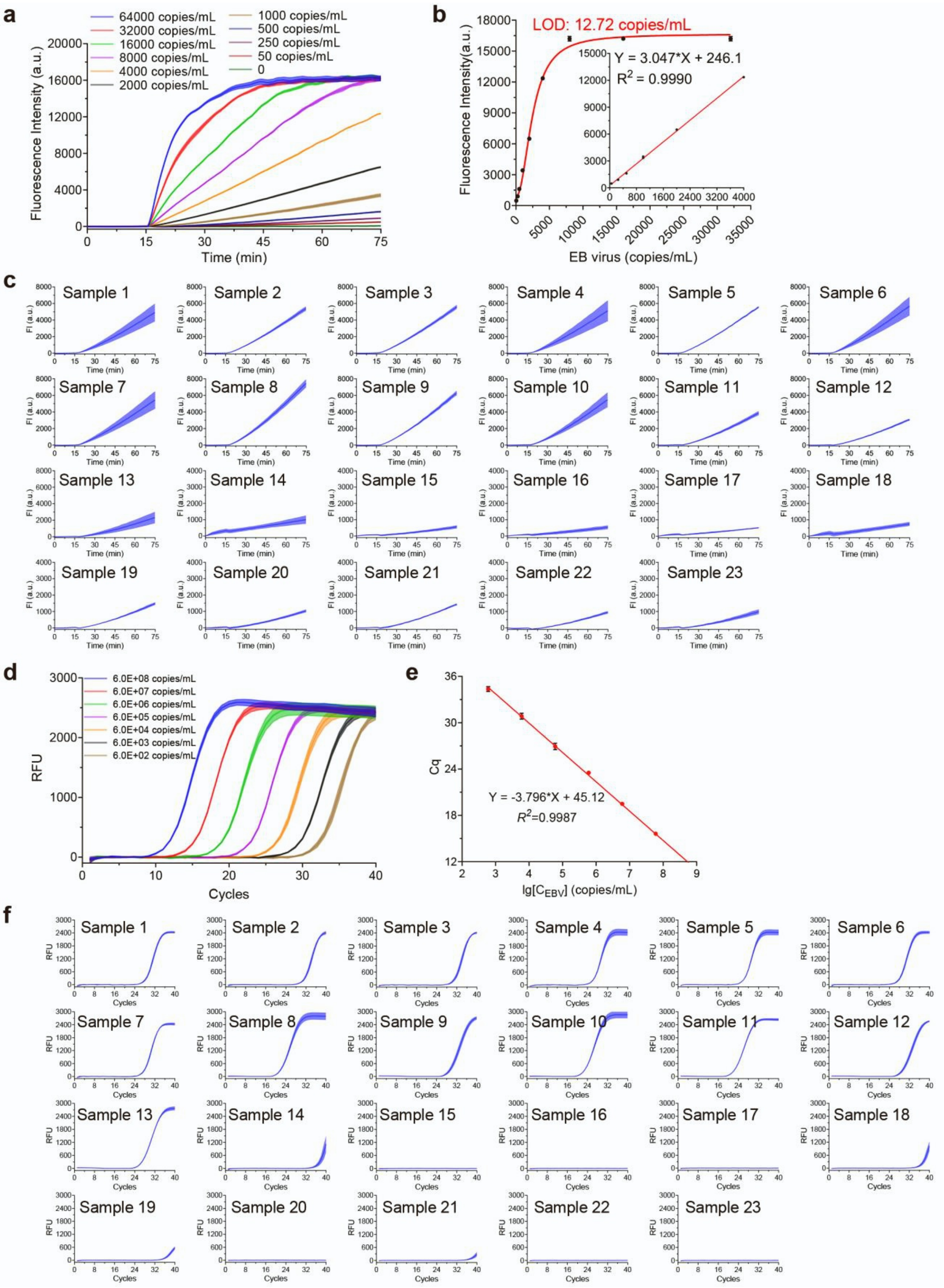
a
64000 copies/mL
32000 copies/mL
16000 copies/mL
8000 copies/mL
4000 copies/mL
2000 copies/mL
1000 copies/mL
500 copies/mL
250 copies/mL
50 copies/mL
0
Fluorescence Intensity (a.u.)
Time (min)
b
LOD: 12.72 copies/mL
Y = 3.047*X + 246.1
R2 = 0.9990
Fluorescence Intensity(a.u.)
EB virus (copies/mL)
c
Sample 1
Sample 2
Sample 3
Sample 4
Sample 5
Sample 6
Sample 7
Sample 8
Sample 9
Sample 10
Sample 11
Sample 12
Sample 13
Sample 14
Sample 15
Sample 16
Sample 17
Sample 18
Sample 19
Sample 20
Sample 21
Sample 22
Sample 23
FI (a.u.)
Time (min)
d
6.0E+08 copies/mL
6.0E+07 copies/mL
6.0E+06 copies/mL
6.0E+05 copies/mL
6.0E+04 copies/mL
6.0E+03 copies/mL
6.0E+02 copies/mL
RFU
Cycles
e
Y = -3.796*X + 45.12
R2=0.9987
Cq
lg[CEBV] (copies/mL)
f
RFU
Cycles

Figure S3: Development and verification of the RPA-CRISPR Cas12a one-pot method assisted by circular crRNA with a PC linker, related to Figure 2. (a) Fluorescent graphs of the detection of different concentrations of EB virus using this method. Error bars represent mean ± S.D. (n = 3). (b) Linear fit graphs of EB virus detection. (c) Raw fluorescence data recording for detection of clinical blood samples by current developed method, infected or not with the EB virus. First, nucleic acids were extracted from 200 μL of whole blood samples, eluted to 20 μL nuclease-free ddH_2O, then 2 μL of each sample was added to the current one-pot detection system, and the volume was filled up to 20 μL with nuclease-free ddH_2O. After 15 minutes of RPA at 37 °C, the samples were exposed to UV light for 30s, and fluorescence variations are then monitored and recorded at 37 °C. Detection of EB virus using the traditional qPCR method (d) Fluorescent graphs for detecting different concentrations of the EB virus using the traditional qPCR method, and (e) the corresponding linear fit graphs. (f) Raw fluorescence data recordings for the detection of clinical blood samples using the traditional qPCR method, in order to make a comparison with Figure S3c.

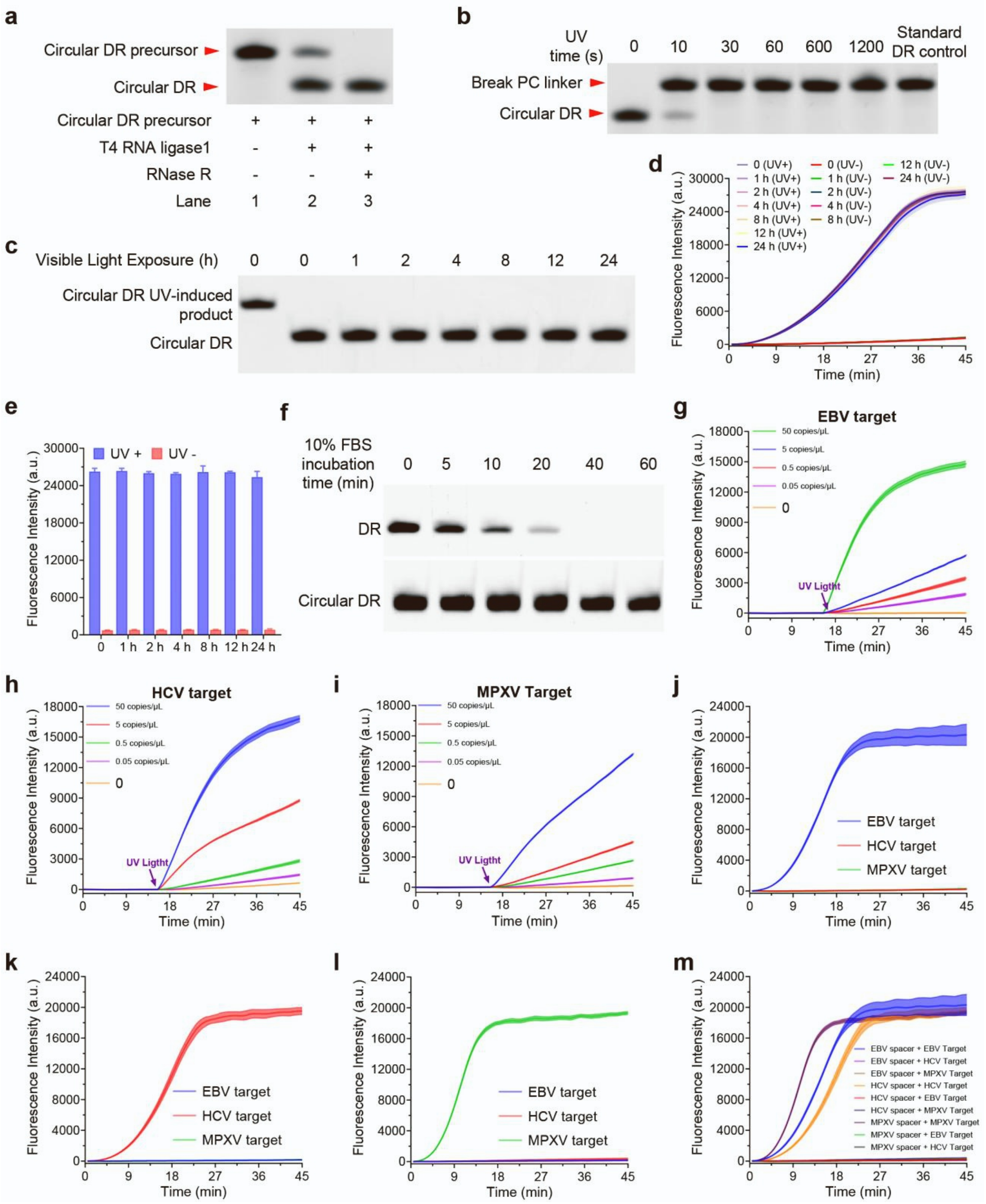

Figure S4: Comprehensive characterization and functional validation of circular DR with PC linker stability and development of RPA-CRISPR/Cas12a one-pot method for different target detection, related to Figure 3. (a) PAGE analysis of Circular DR with a PC linker precursor and its

circularization product. Circularization of the precursor was achieved using T4 RNA Ligase 1, followed by RNase R treatment to eliminate linear molecules, including intermolecular ligation products and unreacted starting materials. (b) UV irradiation effects on the PC linker of Circular DR. Circular DR with a PC linker was exposed to UV light (365 nm, 35 W) for 0-1200 s, and its integrity was assessed by 12% denaturing PAGE (150 V, 30 min). (c) Structural stability of Circular DR with a PC linker under laboratory lighting. Circular DR with a PC linker (100 ng) was incubated in transparent 0.2 mL microcentrifuge tubes on ice for 0, 1, 2, 4, 8, 12, and 24 hours. UV-induced cleavage products served as positive controls. PAGE analysis confirmed the robust stability of the PC linker under standard laboratory conditions. (d) Real-time fluorescence monitoring of LbCas12a trans-cleavage activity. Circular DR with a PC linker + EBV Spacer was exposed to standard laboratory lighting versus unexposed controls over 24 hours. No significant variation in LbCas12a activation was observed, demonstrating the photostability of the circular RNA design. (e) Quantitative fluorescence intensity analysis. Fluorescence intensity at the 45-minute time point from panel (d) was quantified and presented as a bar graph for direct comparison. (f) Environmental stability of Circular DR with a PC linker. Circular DR with a PC linker and canonical DR (100 ng each) were incubated with DMEM + 10% FBS at 37 °C for 0, 5, 10, 20, 40, and 60 minutes. PAGE analysis revealed that canonical DR degraded significantly within 10 minutes and completely by 40 minutes, while Circular DR with a PC linker maintained full integrity throughout the 60-minute incubation. (g) Development and verification of the RPA-CRISPR Cas12a one-pot method assisted by circular DR, to confirm its versatility for detecting various targets when combined with different Spacer regions. Fluorescent graphs of the detection of different concentrations of the EB virus. (h) Fluorescent graphs of the detection of different concentrations of the Hepatitis C virus (HCV). (i) Fluorescent graphs of the detection of different concentrations of Monkeypox virus (MPXV). Cross-verification of the detection of different viruses (EBV, HCV, MPXV) using the universal circular DR combined with different Spacers. (j) Fluorescent graphs of the detection of different viruses (EBV, HCV, MPXV) using the universal circular DR combined with EBV Spacer. Only EBV Target can be detected. (k) Fluorescent graphs of the detection of different viruses using the current method combined with MPXV Spacer. Only MPXV Target can be detected. (l) Fluorescent graphs of the detection of different viruses using the current method combined with HCV Spacer. Only HCV Target can be detected. (m) Raw fluorescence data recording for cross-verification of the detection of different viruses in Figure 3f. All experiments were performed in triplicate; error bars represent mean ± S.D. of triplicate measurements, ensuring statistical reliability.

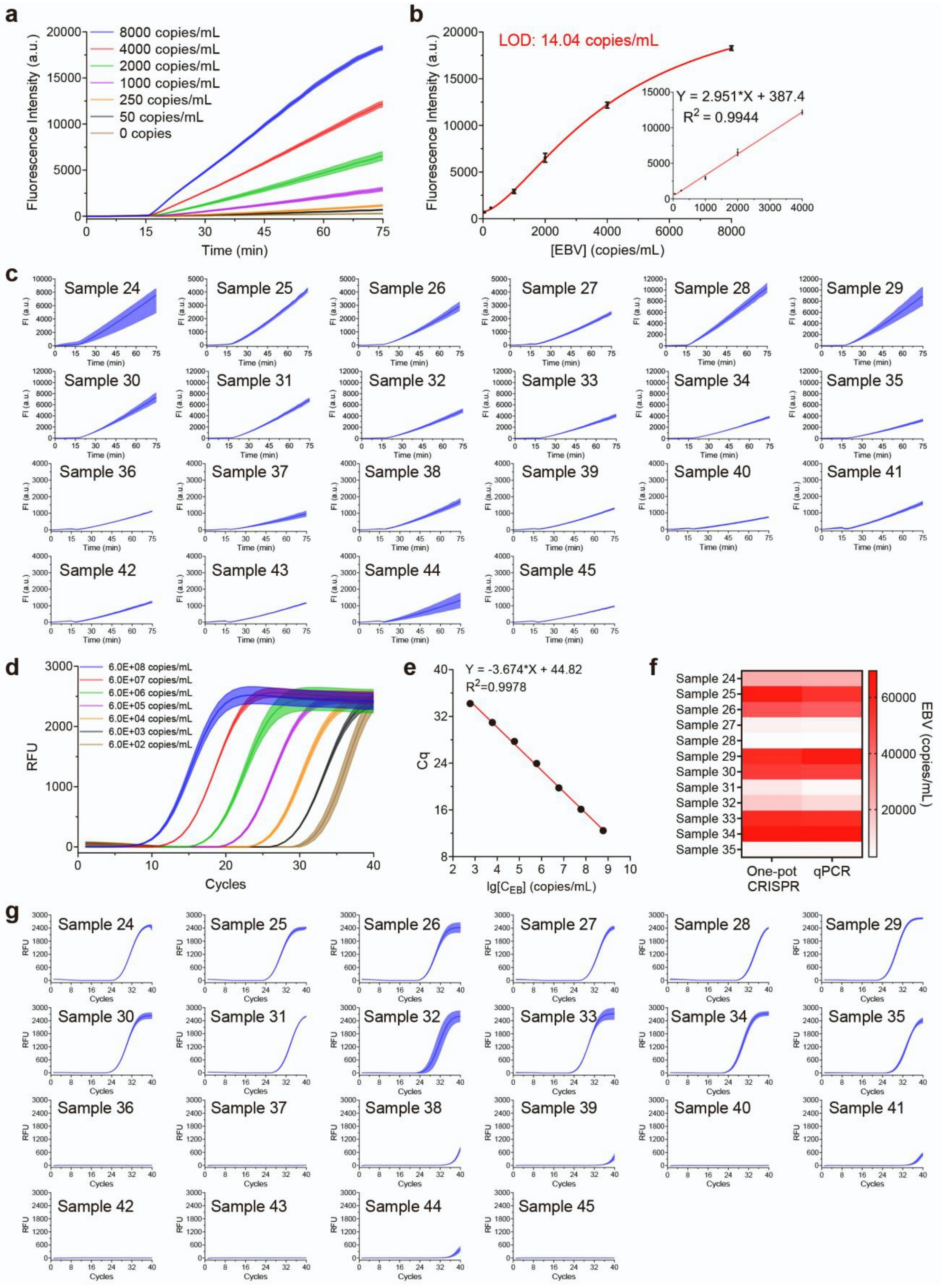
a
8000 copies/mL
4000 copies/mL
2000 copies/mL
1000 copies/mL
250 copies/mL
50 copies/mL
0 copies
Fluorescence Intensity (a.u.)
Time (min)
b
LOD: 14.04 copies/mL
Y = 2.951*X + 387.4
R^2 = 0.9944
Fluorescence Intensity (a.u.)
[EBV] (copies/mL)
c
Sample 24
Sample 25
Sample 26
Sample 27
Sample 28
Sample 29
Sample 30
Sample 31
Sample 32
Sample 33
Sample 34
Sample 35
Sample 36
Sample 37
Sample 38
Sample 39
Sample 40
Sample 41
Sample 42
Sample 43
Sample 44
Sample 45
FI (a.u.)
Time (min)
d
6.0E+08 copies/mL
6.0E+07 copies/mL
6.0E+06 copies/mL
6.0E+05 copies/mL
6.0E+04 copies/mL
6.0E+03 copies/mL
6.0E+02 copies/mL
RFU
Cycles
e
Y = -3.674*X + 44.82
R^2=0.9978
Cq
lg[C_{EB}] (copies/mL)
f
One-pot CRISPR
qPCR
EBV (copies/mL)
g
RFU
Cycles

Figure S5: Development and verification of the universal circular DR assisted RPA-CRISPR Cas12a one-pot method, related to Figure 3. (a) Fluorescent graphs of the detection of different concentrations of EB virus using this method. Error bars represent mean ± S.D. (n = 3). (b) Linear fit graphs of EB virus detection. (c) Raw fluorescence data recording for detection of clinical blood samples by current developed method, infected or not with the EB virus. Detection of EB virus using the traditional qPCR method, in order to make a comparison with Figures S5a-c. (d) Fluorescent graphs for detecting different concentrations of the EB virus using the traditional qPCR method, and (e) the corresponding linear fit graphs. (f) Comparisons of the detections of clinical blood samples using the universal DR-assisted one-pot RPA-CRISPR Cas12a method and the traditional qPCR method. (g) Raw fluorescence data recordings for the detection of clinical blood samples using the traditional qPCR method, in order to make a comparison with Figure S5c.

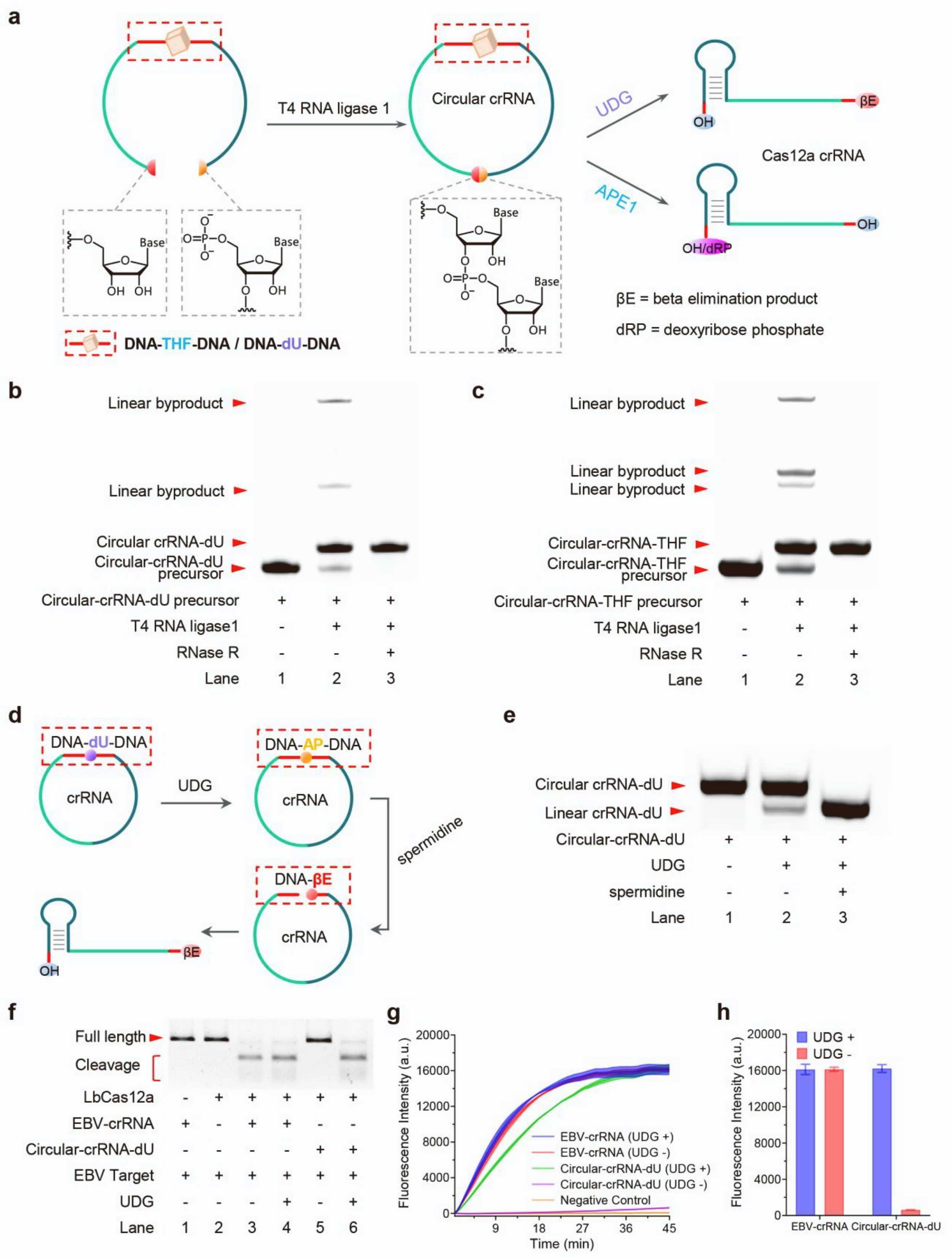
a
T4 RNA ligase 1
Circular crRNA
UDG
APE1
OH
βE
Cas12a crRNA
OH/dRP
OH
Base
DNA-THF-DNA / DNA-dU-DNA
βE = beta elimination product
dRP = deoxyribose phosphate
b
Linear byproduct
Linear byproduct
Circular crRNA-dU
Circular-crRNA-dU precursor
Circular-crRNA-dU precursor + + +
T4 RNA ligase1 - + +
RNase R - - +
Lane 1 2 3
c
Linear byproduct
Linear byproduct
Linear byproduct
Circular-crRNA-THF
Circular-crRNA-THF precursor
Circular-crRNA-THF precursor + + +
T4 RNA ligase1 - + +
RNase R - - +
Lane 1 2 3
d
DNA-dU-DNA
crRNA
UDG
DNA-AP-DNA
spermidine
DNA-βE
OH
βE
e
Circular crRNA-dU
Linear crRNA-dU
Circular-crRNA-dU + + +
UDG - + +
spermidine - - +
Lane 1 2 3
f
Full length
Cleavage
LbCas12a - + + + + +
EBV-crRNA + - + + - -
Circular-crRNA-dU - - - - + +
EBV Target + + + + + +
UDG - - - + - +
Lane 1 2 3 4 5 6
g
Fluorescence Intensity (a.u.)
Time (min)
EBV-crRNA (UDG +)
EBV-crRNA (UDG -)
Circular-crRNA-dU (UDG +)
Circular-crRNA-dU (UDG -)
Negative Control
h
UDG +
UDG -
Fluorescence Intensity (a.u.)
EBV-crRNA
Circular-crRNA-dU

Figure S6: Preparation and verification of circular crRNA with dU or THF modifications, related to Figure 4. (a) A schematic illustration of the preparation of circular crRNA with dU or THF modifications, and the subsequent disruption at the modified base of the circular RNA under UDG or APE1 conditions, reverting to its classic configuration. PAGE analysis showing the successful circulation of circular crRNA with dU (b) or THF modifications (c). Verification of the working principle of circular crRNA with dU modification. (d) Schematic of the working principle. dU is going to form AP Sites acting under UDG. Then, the addition reaction occurs between the AP sites with spermidine in the buffer solution, forming a beta elimination product (βE), opening the circular structure, and restoring the circular crRNA to its classic structure. (e) PAGE analysis to verify the principle in (d). Breakdown products are formed under the action of UDG and spermidine, consistent with the research report by Kent S Gate, et al (*Chem. Res. Toxicol.* 2022, 35, 218-232). Inhibition and restoration of *cis*- and *trans*-cleavage activities of the CRISPR-Cas12a system mediated by dU-modified circular crRNA and UDG enzyme. (f) Comparison of *cis*-cleavage activity in the CRISPR-Cas12a system facilitated by dU-modified circular crRNA versus conventional EBV crRNA, alongside the recovery of *cis*-cleavage activity following UDG enzyme treatment of the dU-modified circular crRNA. Reactions were incubated for 45 minutes. (g) Evaluation of *trans*-cleavage activity in the CRISPR-Cas12a system using dU-modified circular crRNA versus EBV crRNA, and the restoration of *trans*-cleavage activity upon UDG enzyme treatment of the dU-modified circular crRNA. (h) Bar graph representation of the fluorescence intensity at 45 minutes for the conditions described in panel (g), providing a quantitative comparison of fluorescence intensity generated by *trans*-cleavage activity.

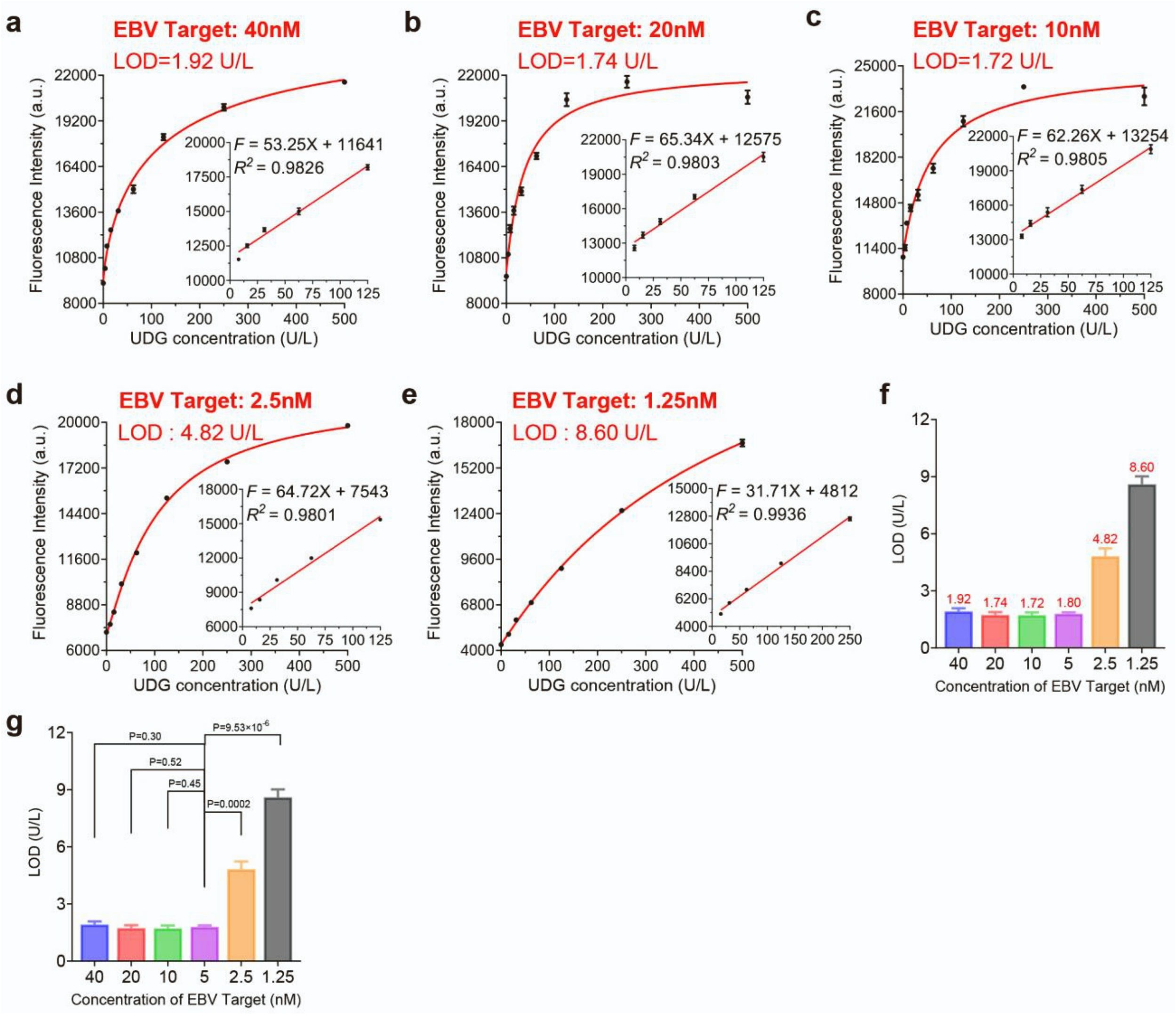

Figure S7: Relationship between initial EBV target concentration and UDG detection limit in the circular crRNA-based detection system, related to Figure 4. (a) UDG detection limit evaluated at 40 nM EBV target concentration. (b) UDG detection limit evaluated at 20 nM EBV target concentration. (c) UDG detection limit evaluated at 10 nM EBV target concentration. (d) UDG detection limit evaluated at 2.5 nM EBV target concentration. (e) UDG detection limit evaluated at 1.25 nM EBV target concentration. (f) Comparative bar graph illustrating the detection limits across EBV target concentrations of 40 nM, 20 nM, 10 nM, 2.5 nM, 1.25 nM, and 5 nM (as shown in Figure 3b of the main text). (g) Corresponding p-values from statistical analysis, where $p > 0.05$ indicates no significant differences. All experiments were performed in triplicate ($n = 3$), with error bars representing mean ± S.D.

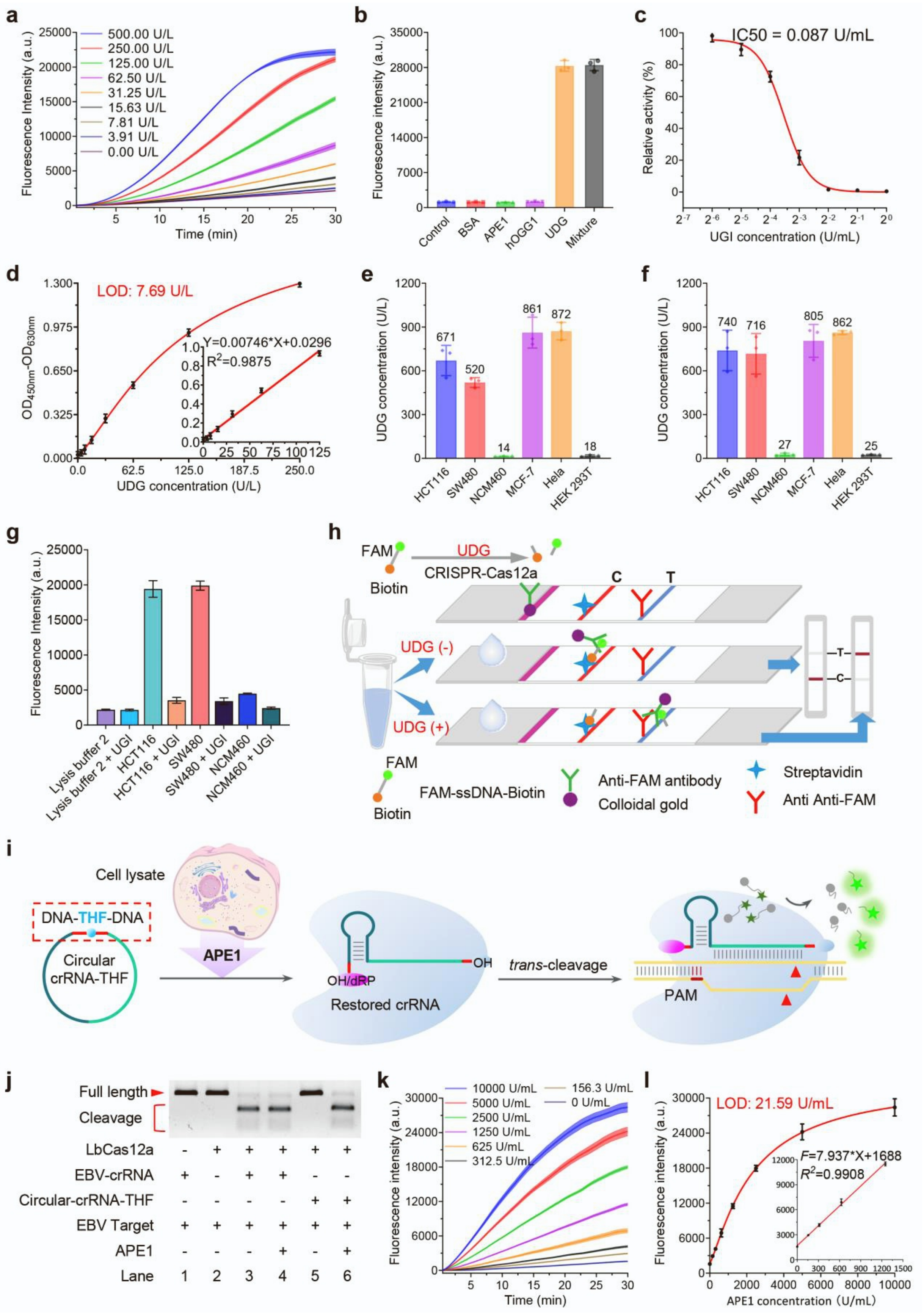
a
500.00 U/L
250.00 U/L
125.00 U/L
62.50 U/L
31.25 U/L
15.63 U/L
7.81 U/L
3.91 U/L
0.00 U/L
Fluorescence Intensity (a.u.)
Time (min)
b
Fluorescence intensity (a.u.)
Control
BSA
APE1
hOGG1
UDG
Mixture
c
IC50 = 0.087 U/mL
Relative activity (%)
UGI concentration (U/mL)
d
LOD: 7.69 U/L
Y=0.00746*X+0.0296
R2=0.9875
OD450nm-OD630nm
UDG concentration (U/L)
e
UDG concentration (U/L)
671
520
14
861
872
18
HCT116
SW480
NCM460
MCF-7
Hela
HEK 293T
f
UDG concentration (U/L)
740
716
27
805
862
25
HCT116
SW480
NCM460
MCF-7
Hela
HEK 293T
g
Fluorescence Intensity (a.u.)
Lysis buffer 2
Lysis buffer 2 + UGI
HCT116
HCT116 + UGI
SW480
SW480 + UGI
NCM460
NCM460 + UGI
h
FAM
UDG
CRISPR-Cas12a
Biotin
C
T
UDG (-)
UDG (+)
FAM
Biotin
FAM-ssDNA-Biotin
Anti-FAM antibody
Colloidal gold
Streptavidin
Anti Anti-FAM
i
Cell lysate
DNA-THF-DNA
Circular
crRNA-THF
APE1
OH
OH/dRP
Restored crRNA
trans-cleavage
PAM
j
Full length
Cleavage
LbCas12a
EBV-crRNA
Circular-crRNA-THF
EBV Target
APE1
Lane
1
2
3
4
5
6
k
10000 U/mL
5000 U/mL
2500 U/mL
1250 U/mL
625 U/mL
312.5 U/mL
156.3 U/mL
0 U/mL
Fluorescence intensity (a.u.)
Time (min)
l
LOD: 21.59 U/mL
F=7.937*X+1688
R2=0.9908
Fluorescence intensity (a.u.)
APE1 concentration (U/mL)

Figure S8: Verification of the selectivity of the detection of UDG by the CRISPR-Cas12a system, assisted by circular RNA with dU modification, related to Figure 4. (a) Raw fluorescence data graphs for varying concentrations detected by current detection system. (b) The fluorescence graphs of Bovine Serum Albumin (BSA), APE1, Recombinant Human OGG1 (hOGG1), and Uracil-DNA Glycosylase (UDG), as well as their mixtures tested in the current detection system, validating the specificity of the system for UDG detection. (c) Suppression of the Circular-crRNA-dU assisted CRISPR Cas12 detection system by UGI, UDG's inhibitor, demonstrating Circular-crRNA-dU assisted CRISPR Cas12 detection system regulation through various UGI concentrations. The calculated IC50 for UGI is 0.087 U/mL. (d) The linear fit graphs corresponding to the commercial Human Uracil-DNA Glycosylase (UNG) ELISA Kit (Ziker, cat. no. ZK-H2551) accurately detect the known UDG concentration. (e) A bar chart displaying the levels of UDG in various cell lines as detected by the currently developed methods. (f) A bar chart illustrating the levels of UDG in different cell lines as detected by the commercial Human Uracil-DNA Glycosylase (UNG) ELISA Kit. (g) Assessment of UDG detection system compatibility with various cell lysates. Fluorescence intensity profiles of HCT116, SW480, and NCM460 cell lysates, including samples supplemented with UGI, were measured using the current UDG detection system at the 30-minute time point. These results demonstrate the system's applicability beyond nuclear protein extracts, validating its robustness for broader biological sample analysis. (h) A schematic depiction of the paper strip-based visual detection device designed utilizing the current developed CRISPR-Cas12a system, supported by circular RNA with dU modification. CRISPR-Cas12a system assisted by crRNA with THF modification. (i) Schematic of the working principle of the CRISPR-Cas12a system aided by circular crRNA with THF modification. (j) PAGE analysis validating the inhibition of the *cis*-cleavage activity of the CRISPR-Cas12a system by circular crRNA with THF modification, and its reactivation by APE1. Varying concentrations of APE1 detected by the system show the suppression of *trans*-cleavage activity by circular crRNA with THF modification (0.00 U/mL), and its subsequent reactivation by APE1 with fluorescence intensity increasing with concentration. (k-l) The correlation between the fluorescent values (k) and the linear fit (l) of different concentrations of APE1 detected by the CRISPR-Cas12a system, assisted by circular crRNA with THF modification. All experiments were performed in triplicate, and error bars represent mean ± S.D. (n = 3).

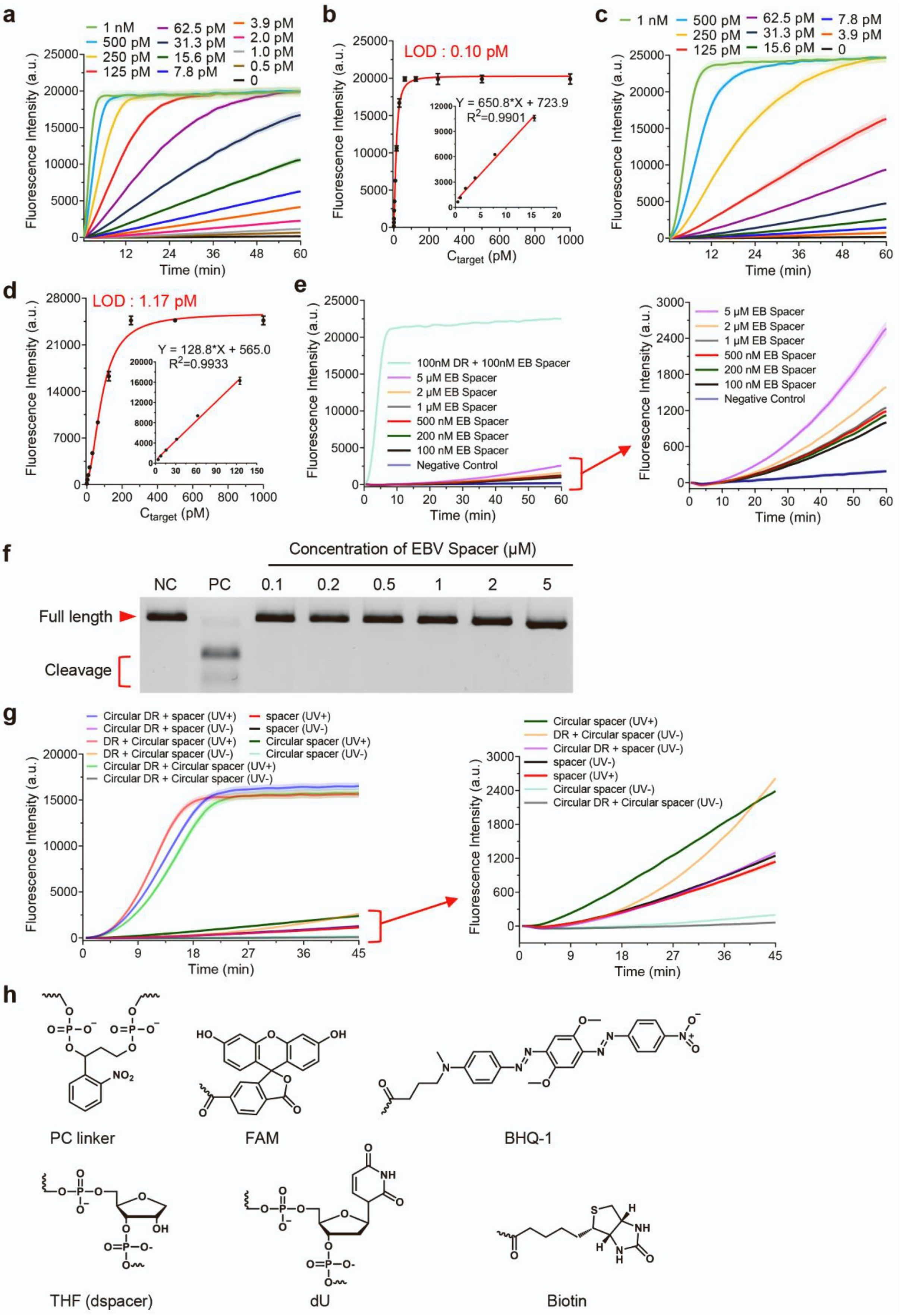

a
b
c
d
e
f
g
h
LOD : 0.10 pM
LOD : 1.17 pM
Y = 650.8*X + 723.9
R^2=0.9901
Y = 128.8*X + 565.0
R^2=0.9933
Fluorescence Intensity (a.u.)
Time (min)
C_{target} (pM)
1 nM
500 pM
250 pM
125 pM
62.5 pM
31.3 pM
15.6 pM
7.8 pM
3.9 pM
2.0 pM
1.0 pM
0.5 pM
0
100nM DR + 100nM EB Spacer
5 μM EB Spacer
2 μM EB Spacer
1 μM EB Spacer
500 nM EB Spacer
200 nM EB Spacer
100 nM EB Spacer
Negative Control
Concentration of EBV Spacer (μM)
NC
PC
0.1
0.2
0.5
1
2
5
Full length
Cleavage
Circular DR + spacer (UV+)
Circular DR + spacer (UV-)
DR + Circular spacer (UV+)
DR + Circular spacer (UV-)
Circular DR + Circular spacer (UV+)
Circular DR + Circular spacer (UV-)
spacer (UV+)
spacer (UV-)
Circular spacer (UV+)
Circular spacer (UV-)
PC linker
FAM
BHQ-1
THF (dspacer)
dU
Biotin

Figure S9: Evaluation of EBV crRNA and split crRNA systems for target detection, related to Figures 2, 3, 4, 5. (a) Real-time fluorescence intensity of full-length EBV-crRNA detecting EBV Target at varying concentrations under identical experimental conditions. (b) Calibration curve derived from (a), showing the relationship between fluorescence intensity and target concentration, including linear regression analysis and limit of detection (LOD). (c) Real-time fluorescence intensity of split crRNA (DR + EBV Spacer) detecting EBV Target at varying concentrations under identical conditions. (d) Calibration curve derived from (c), displaying the fluorescence-concentration relationship, linear regression, and LOD. All experiments were performed in triplicate, and error bars represent mean ± S.D. (n = 3). (e) Fluorescence comparison of *trans*-cleavage activity of LbCas12a directed by EBV Spacer alone (100 nM-5 μM) versus the combined DR (100 nM) and EBV Spacer (100 nM) system. (f) Agarose gel (2%) analysis of *cis*-cleavage activity of LbCas12a directed by EBV Spacer alone (100 nM-5 μM) versus the combined DR (100 nM) and EBV Spacer (100 nM) system. NC: EBV Target alone, in the absence of both DR and Spacer; PC: positive control (100 nM DR + 100 nM EBV Spacer); 0.1-5: concentrations of EBV Spacer in the absence of both DR (μM). (g) *trans*-cleavage activity comparison of circular DR+Spacer, DR+circular Spacer, and circular DR + circular Spacer under UV exposure and non-exposure conditions. Circular DR+circular Spacer exhibits the lowest background fluorescence. (h) Chemical Structures Corresponding to the Abbreviations of Nucleic Acid Modifications Used in the Manuscript.

Table S1: Oligonucleotide (ODN) sequences and modifications used in this study, related to Figures 1, 2, 3, 4, 5. The abbreviations and modifications include: /ideoxyU/ for deoxyUridine modification, /PC Linker/ for a UV-photocleavable C3 spacer arm linking two nucleotide sequences at any position, /5Phos/ for 5' Phosphorylation modification, /THF/ for dSpacer (1,2'-Dideoxyribose) modification, /56-FAM/ for 5' 6-FAM (Fluorescein) modification, /BHQ1/ for Black Hole Quencher-1 used to quench green and yellow dyes such as FAM, TET, and HEX, and /5mC/ for 5-methyl-2'-deoxycytidine.

Name	Sequence (5'-3')
EBV crRNA	AAU UUC UAC UAA GUG UAG AUC AAA CUC AUA UAU UUG CUG A
EBV-Cir-crRNA precursor	/5Phos/ CAA ACU CAU AUA UUU GCU GA /PC Linker/ A AUU UCU ACU AAG UGU AGA U
DR	AAU UUC UAC UCU UGU AGA U
Circular DR precursor	/5Phos/ACU CUU GUA GAU/PC Linker/ AAU UUC U
EBV spacer	CAA ACU CAU AUA UUU GCU GA
HCV spacer	GGC GUG CCC CCG CAA GAC UG
MPXV spacer	GUA UAU AAG UUG UAC GGC UAA UUC
SEPT spacer	AGC GGG GUU CGA GAG UCG UC
Circular-crRNA-dU precursor	/5Phos/ CAA ACU CAU AUA UUU GCU dGdAdT /ideoxyU/dAdC dAAA UUU CUA CUC UUG UAG AU
Circular-crRNA-THF precursor	/5Phos/ CAA ACU CAU AUA UUU GCU dGdA/THF/dT dAAA UUU CUA CUC UUG UAG AU
F-Q	/56-FAM/-CCC CCC CC-/BHQ1/
AAVS1 crRNA	UAA UUU CUA CUA AGU GUA GAU UCU GUC CCC UCC ACC CCA CAG UG
AAVS1-Cir-crRNA precursor	/5Phos/UCU GUC CCC UCC ACC CCA CAG UG/PC linker/A AUU UCU ACU AAG UGU AGA UC
AAVS1-PCR-F	GTT CTC CTG TGG ATT CGG GTC
AAVS1-PCR-R	TGA CGC ACG GAG GAA CAA TAT
EBV-RPA-F	GAC CCG GCC CAC AAC CTG GCC CAC TAA GGG
EBV-RPA-R	ACT CCA TCG TCA AAG CTG CAC ACA GTC ACC
HCV-RPA-F	GGG TCC TTT CTT GGA TAA ACC CGC TCA ATG C
HCV-RPA-R	CTC GCA AGC ACC CTA TCA GGC AGT ACC ACA A
B6R-RPA-F	CTA ATG CGG AAT GTC AAC CTC TTC AA
B6R-RPA-R	AGA AAA TGT AGA TCC GGA AAT TAA TC
SEPT9-5mC-Target	GGCTGTCCACTCAGTCGGAGGTGAGGAACGACCTCCCTATCCCGTTGC CGGGTCCAAG/5mC/GGGGCC/5mC/GAGAGC/5mC/GC/5mC/GGGGAGAG CCAAAGGGAGGGGACCGATGGATTTCCCAGAGTGAAACTGTGCGTCCT GGAGAGTTCCGAGGCAGCCTCGCGAGCCCTCGAGGAGGTCGCTGTCGC TTGG
SEPT9-C-Target	GGCTGTCCACTCAGTCGGAGGTGAGGAACGACCTCCCTATCCCGTTGC CGGGTCCAAGCGGGGCCCGAGAGCCGCCGGGGAGAGCCAAAGGGAGG GGACCGATGGATTTCCCAGAGTGAAACTGTGCGTCCTGGAGAGTTCCG AGGCAGCCTCGCGAGCCCTCGAGGAGGTCGCTGTCGCTTGG
FAM-Biotin	/56-FAM/-CCC CCC CC-/Biotin/
EBV Target	ATGTCGTATTACACCATTGAGTCGTCTCCCCTTTGGAATGGCCCCTGGA CCCGGCCCACAACCTGGCCCACTAAGGGAGTCCATTGTCTGTTATTTCA TGGTCTTTTTACAAACTCATATATTTGCTGAGGTTTTGAAGGATGCGAT TAAGGACCTTGTTATGACAAAGCCCGCTCCTACCTGCAATATCAAGGT GACTGTGTGCAGCTTTGACGATGGAGTAGATTTGCCTCCCTGGTTTCCA CCTATGGTGGAAGGGGCTGCCGCGGAGGGTGATGACGGAGATGACGG AGATGAAGGAGGTGATGGAGATGAGGGTGAGGAAGGGCAGGAGTGAT GTAACTTGTTAGGAGACGC
MPXV Target	GTAAAACGACGGCCAGTGAATTCGAGCTCGGTACCTCGCGAATGCATC

	TAGAATGAAAACGATTTCCGTTGTTACGTTGTTATGCGTACTACCTGCT<br>GTTGTTTATTCAACATGTACTGTACCCACTATGAATAACGCTAAATTAA<br>CGTCTACCGAAACATCGTTTAATGATAAACAGAAAGTTACGTTTACAT<br>GTGATTCAGGATATCATTCTTTGGATCCAAATGCTGTCTGTGAAACAGA<br>TAAATGGAAATACGAAAATCCATGCAAGAAAATGTGCACAGTTTCTGA<br>TTATGTCTCTGAACTATATGATAAGCCATTATACGAAGTGAATTCCACC<br>ATGACACTAAGTTGCAACGGTGAAACAAAATATTTTCGTTGTGAAGAA<br>AAAAATGGAAATACTTCTTGGAATGATACTGTCACGTGTCCTAATGCG<br>GAATGTCAACCTCTTCAATTAGAACACGGATCGTGTCAACCAGTTAAA<br>GAAAAATACTCATTTGGGGAATATATGACTATCAACTGTGATGTTGGA<br>TATGAGGTTATTGGTGTTTCGTATATAAGTTGTACGGCTAATTCTTGGA<br>ATGTTATTCCATCATGTCAACAAAAATGTGATATACCGTCCCTATCTAA<br>TGGATTAATTTCCGGATCTACATTTTCTATCGGTGGCGTTATACATCTT<br>AGTTGTAAAAGTGGTTTACACTAACGGGGTCTCCATCATCCACATGTA<br>TCGACGGTAAATGGAATCCCATACTCCCAACATGTGTACGATCTAACG<br>AAGAATTTGATCCAGTGGATGATGGTCCCGACGATGAGACAGATCTGA<br>GCAAACTCTCGAAAGACGTTGTACAATATGAACAAGAAATAGAATCGT<br>TAGAAGCAACTTATCATATAATCATAATGGCGTTGACAATTATGGGTG<br>TCATATTTCTAATCTCCATTATAGTATTAGTTTGTTCCTGTGACAAAAA<br>TAATGACCAATATAAGTTCCATAAATTGCTACCGTGACCCGGGCCCGT<br>CGACTGCAGAGGCCTGCATGCAAGCTTGGCGTAATCATGGTCATAGCT<br>GTTTCCTG
HCV Target	GTAAAACGACGGCCAGTGAATTCGAGCTCGGTACCTCGCGAATGCATC<br>TAGATAATACGACTCACTATAGGGAACTACTGTCTTCACGCAGAAAGC<br>GTCTAGCCATGGCGTTAGTATGAGTGTCGTGCAGCCTCCAGGACCCCC<br>CCTCCCGGGAGAGCCATAGTGGTCTGCGGAACCGGTGAGTACACCGGA<br>ATTGCCAGGACGACCGGGTCCTTTCTTGGATAAACCCGCTCAATGCCT<br>GGAGATTTGGGCGTGCCCCCGCAAGACTGCTAGCCGAGTAGTGTTGGG<br>TCGCGAAAGGCCTTGTGGTACTGCCTGATAGGGTGCTTGCGAGTGCCC<br>CGGGAGGTCTCGTAGACCGTGCACCATGAGCACGAATCCTAAACCTCA<br>AAGACCCGGGCCCGTCGACTGCAGAGGCCTGCATGCAAGCTTGGCGTA<br>ATCATGGTCATAGCTGTTTCCTG
AAVS1 Target	GTTCTCCTGTGGATTCGGGTCACCTCTCACTCCTTTCATTTGGGCAGCT<br>CCCCTACCCCCCTTACCTCTCTAGTCTGTGCTAGCTCTTCCAGCCCCCT<br>GTCATGGCATCTTCCAGGGGTCCGAGAGCTCAGCTAGTCTTCTTCCTCC<br>AACCCGGGCCCTATGTCCACTTCAGGACAGCATGTTTGCTGCCTCCAG<br>GGATCCTGTGTCCCCGAGCTGGGACCACCTTATATTCCCAGGGCCGGTT<br>AATGTGGCTCTGGTTCTGGGTACTTTTATCTGTCCCCTCCACCCCACAG<br>TGGGGCCACTAGGGACAGGATTGGTGACAGAAAAGCCCCCATCCTTAG<br>GCCTCCTCCTTCCTAGTCTCCTGATATTCGTCTAACCCCCACCTCCTGTT<br>AGGCAGATTCCTTATCTGGTGACACACCCCCATTTCCTGGAGCCATCTC<br>TCTCCTTGCCAGAACCTCTAAGGTTTGCTTACGATGGAGCCAGAGAGG<br>ATCCTGGGAGGGAGACTTGGCAGGGGGTGGGAGGGAAGGGGGGGATG<br>CGTGACCTGCCCGGTTCTCAGTGGCCACCCTGCGCTACCCTCTCCCAGA<br>ACCTGAGCTGCTCTGACGCGGCTGTCTGGTGCGTTTCACTGATCCTGGT<br>GCTGCAGCTTCCTTACACTTCCCAAGAGGAGAAGCAGTTTGGAAAAAC<br>AAAATCAGAATAAGTTGGTCCTGAGTTCTAACTTTGGCTCTTCACCTTT<br>CTAGNCCCCAATTTATATTGTTCCTCCGTGCGTCA
