# Supplementary material for: Enhancement of CRISPR-Cas12a system through universal circular RNA design
Source: Cell Rep Methods. 2025 Jun 16;5(6):101076. doi: 10.1016/j.crmeth.2025.101076 (PMC12272218; doi:10.1016/j.crmeth.2025.101076)
Supplement: Document S2. Article plus supplemental information [file mmc2.pdf]

Cell Reports
Methods

Article

Enhancement of CRISPR-Cas12a system through universal circular RNA design

Graphical abstract

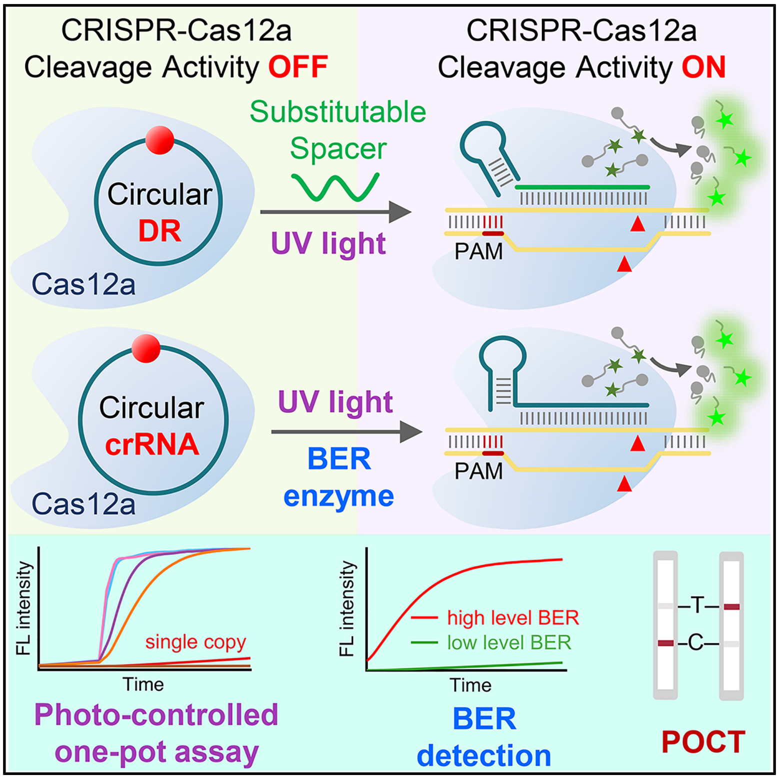

Highlights

- Developed a light-activated circular RNA system for dynamic LbCas12a regulation
- Enabled high-sensitivity, specific detection of trace nucleic acids in clinical samples
- Utilized DNA-modified circular RNA for precise Cas12a control via BER enzymes
- Proposed a strategy for selective DNA cleavage with high BER expression

Authors

Jiaqi Wang, Wei Zhang, Wentao Li, Qinyuan Xie, Ziyu Zang, Chaoxing Liu

Correspondence

liuchx69@mail.sysu.edu.cn

In brief

Wang et al. present a universal circular RNA design enabling precise control of Cas12a activity via UV light or base excision repair enzymes. This system achieves high-sensitivity detection of trace nucleic acids in clinical samples and offers potential for non-nucleic acid target analysis, advancing diagnostics and targeted therapeutic applications.

Wang et al., 2025, Cell Reports Methods *5*, 101076
June 16, 2025 © 2025 The Author(s). Published by Elsevier Inc.
https://doi.org/10.1016/j.crmeth.2025.101076

CellPress

Cell Reports Methods

CellPress
OPEN ACCESS

Article

Enhancement of CRISPR-Cas12a system through universal circular RNA design

Jiaqi Wang,[1,4] Wei Zhang,[1,4] Wentao Li,[2] Qinyuan Xie,[3] Ziyu Zang,[3] and Chaoxing Liu[1,5,*]
[1]Guangdong Provincial Key Laboratory of Digestive Cancer Research, Digestive Diseases Center, Scientific Research Center, The Seventh Affiliated Hospital of Sun Yat-sen University, Shenzhen, Guangdong 518107, P.R. China
[2]Department of Clinical Laboratory, The Seventh Affiliated Hospital of Sun Yat-sen University, Shenzhen, Guangdong 518107, P.R. China
[3]School of Medicine, Sun Yat-sen University, Shenzhen, Guangdong 518107, P.R. China
[4]These authors contributed equally
[5]Lead contact
*Correspondence: liuchx69@mail.sysu.edu.cn
https://doi.org/10.1016/j.crmeth.2025.101076

MOTIVATION The CRISPR-Cas12a system faces limitations in its use in clinical diagnostics, including incompatibility with isothermal amplification and lack of precise activity control. These challenges hinder its application in one-pot nucleic acid detection and non-nucleic acid target analysis. To address these issues, we developed a universal circular RNA system for dynamic LbCas12a regulation. This approach resolves amplification-detection incompatibility and introduces a mechanism for CRISPR-Cas12a control via light triggering or base excision repair enzymes. Our method bridges gaps by offering transformative potential for precise diagnostics and expanding CRISPR-based applications in clinical settings.

SUMMARY

Precise control of Cas12a activity is crucial to address incompatibility in isothermal amplification-CRISPR-Cas12a one-pot nucleic acid detection. We developed a light-triggerable circular RNA system for dynamic LbCas12a regulation. By employing circular CRISPR guide RNA (crRNA) or a split circular universal direct repeat region with a replaceable spacer, we resolved the incompatibility between isothermal amplification and CRISPR detection. This system demonstrated robust performance in detecting trace nucleic acids in clinical samples. Furthermore, DNA modifications on circular crRNA enabled CRISPR-Cas12a regulation via base excision repair (BER) enzymes, offering potential for BER enzyme detection and modulation of LbCas12a cleavage activity by BER enzymes. This programmable strategy holds promise for selective gene editing in cells with elevated BER enzyme expression, such as uracil DNA glycosylase (UDG) in colon cancer cells. The circular RNA-assisted approach represents a resource-efficient method with significant potential for medical diagnostics and future clinical gene therapy applications.

INTRODUCTION

The CRISPR-Cas12a system is a natural immune system found in prokaryotes, such as the Lachnospiraceae bacterium ND2006 (Lb), Acidaminococcus sp. BV3L6 (As) and *Francisella novicida* (Fn).[1–3] The Cas12a protein, also known as Cpf1 in this system is a nuclease that, with the assistance of CRISPR guide RNA (crRNA), can recognize and cleave foreign invasive DNA.[4] One remarkable feature of this system is its *cis*-cleavage activity, enabling it to effectively cleave target double-stranded DNA (dsDNA).[5,6] This makes it a potentially powerful tool in the field of gene editing.[7–10] Additionally, activated CRISPR-Cas12a also possesses *trans*-cleavage activity,[11,12] enabling the cleavage of non-specific single-stranded DNA (ssDNA),[13,14] λDNA,[15] and 3′ overhang dsDNA.[16] A highly effective nucleic acid detection platform has been developed utilizing the *trans*-cleavage activity of CRISPR-Cas12a.[17–19] This technology is regarded as a powerful and immensely promising tool for nucleic acid detection, potentially surpassing qPCR technology to become the new gold standard.[20–22]

DETECTR[14] and HOLMES,[17] methods developed in 2018 using CRISPR-Cas12a, marked the beginning of a period of rapid development for the CRISPR-Cas12a nucleic acid detection system. Typically, CRISPR-Cas12a-based nucleic acid detection systems require specific crRNA design, including a universal direct repeat (DR) region and a spacer region (18–23 nt).[3] The Cas12a protein interacts with the crRNA to form a complex. Activation of this complex occurs when it binds to the target DNA, leading to non-specific cleavage of ssDNA through *trans*-cleavage activity.[13,19] This activity not only cleaves the target DNA but

© 2025 The Author(s). Published by Elsevier Inc.
This is an open access article under the CC BY license (http://creativecommons.org/licenses/by/4.0/).

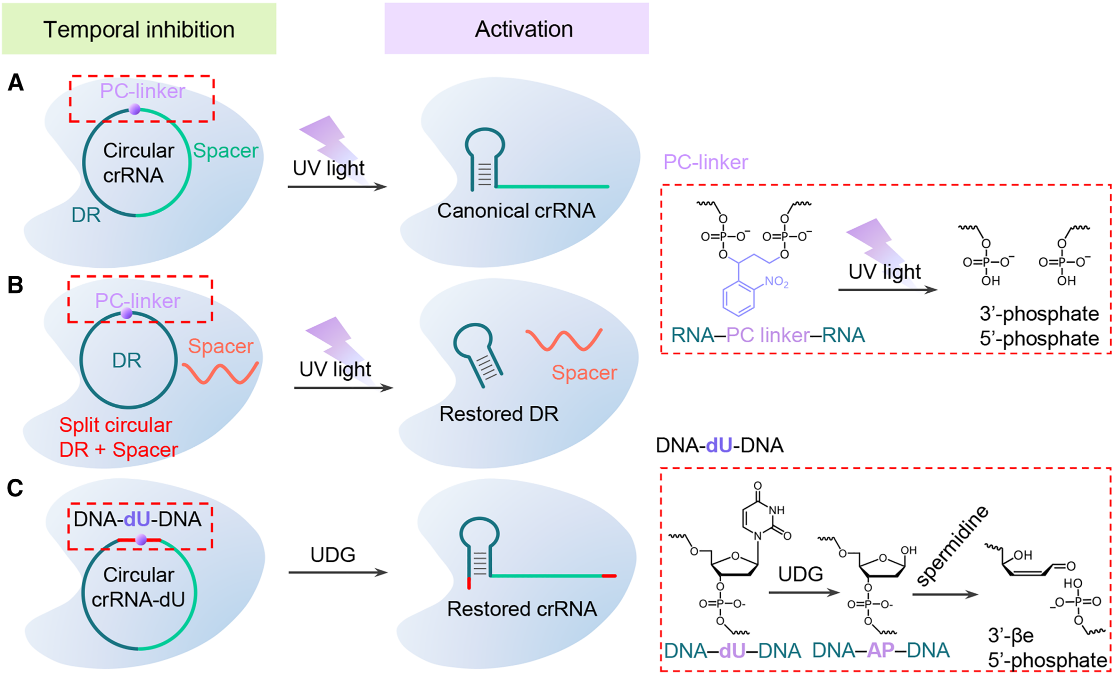

Figure 1. Development and optimization of circular RNA for enhanced CRISPR-Cas12a-based detection

(A) Schematic of circular crRNA incorporating a commercially available PC linker.

(B) Design of a universal circular DR region with a PC linker and a replaceable spacer region, enabling target-specific detection via a photocontrolled one-pot strategy to address compatibility challenges between isothermal amplification and CRISPR-Cas12a.

(C) Introduction of DNA modifications into circular crRNA to regulate the CRISPR-Cas12a system using base excision repair (BER) enzymes.

also ssDNA reporters, such as fluorophore and quencher (F-Q) (Table S1), which contains a fluorophore (FAM) at one end and a quencher (BHQ1) at the other (Figure S9H). This cleavage event leads to the release of a fluorescent signal, as demonstrated in previous studies.[14,17] Monitoring the change in fluorescent intensity allows for the determination of the target DNA content. Combining this technology with isothermal amplification and lateral flow chromatography has proven effective for virus detection, such as COVID-19,[18,23–28] providing excellent results and societal value. Continuous improvement has expanded the application of the CRISPR-Cas12a system to detect RNA (e.g., SAHARA,[29] asymmetric CRISPR technology[30]) and other non-nucleic acid targets.[31,32]

The CRISPR-Cas12a system holds great potential in the field of clinical nucleic acid testing, offering improved detection sensitivity, reduced reliance on large instruments, lower testing costs, and faster detection speed.[20,33–35] It allows for early screening of low copy-number viruses and tumor nucleic acid biomarkers.[30,36,37] However, integrating the isothermal amplification system with the CRISPR reaction in a single tube can hinder nucleic acid amplification efficacy due to the *trans*-cleavage activity of the Cas12a protein, particularly with low copy-number inputs. This can impair amplification efficiency and decrease detection sensitivity.[25,38] Initial versions of DETECTR[14] and HOLMES[17] required separate tubes for amplification and the CRISPR reaction, increasing the complexity of the diagnostic procedure and the potential for false positive results due to liquid transfer and aerosol contamination.[39] Various optimization strategies have been explored to address these challenges, such as physical segregation of reagents,[40,41] specialized buffers,[42] selection of suboptimal protospacer adjacent motif (PAM) sites,[43] and phase-separation solutions.[44] However, conventional approaches have shown limitations or hindered the CRISPR reaction in specific clinical specimen tests, leading to unresolved compatibility issues.[25,38] Through the implementation of temporary-deactivated light-start CRISPR strategies,[45–48] researchers have achieved high-sensitivity detection without the need for separate reaction tubes or reagent transfer.[25,38,49,50] Furthermore, the validation of the inhibitory effect of circular crRNA's topological structure on Cas12a activity offers promising prospects for completely overcoming the compatibility issues between isothermal amplification and CRISPR-Cas12a.[31,51] However, previous Cas12a crRNA circularization methods focused on canonical nucleic acids without chemical modifications, relying solely on nucleic acid enzyme for Cas protein reactivation, limiting their compatibility with clinical isothermal amplification and CRISPR integration.[31] Existing photocontrolled methods, although promising for clinical nucleic acid detection, require target-specific design and synthesis, including uracil substitution[38]—a challenge for uracil-free sequences—or the addition of complementary sequences,[25] increasing complexity and reducing versatility. A universal strategy that necessitates only a single modification of nucleic acids for the detection of arbitrary targets is urgently required.

Here, we present a method called CIRCLE (enhancement of CRISPR-Cas12a system through universal circular RNA design) (Figure 1). This method focuses on incorporating a

CellPress
OPEN ACCESS

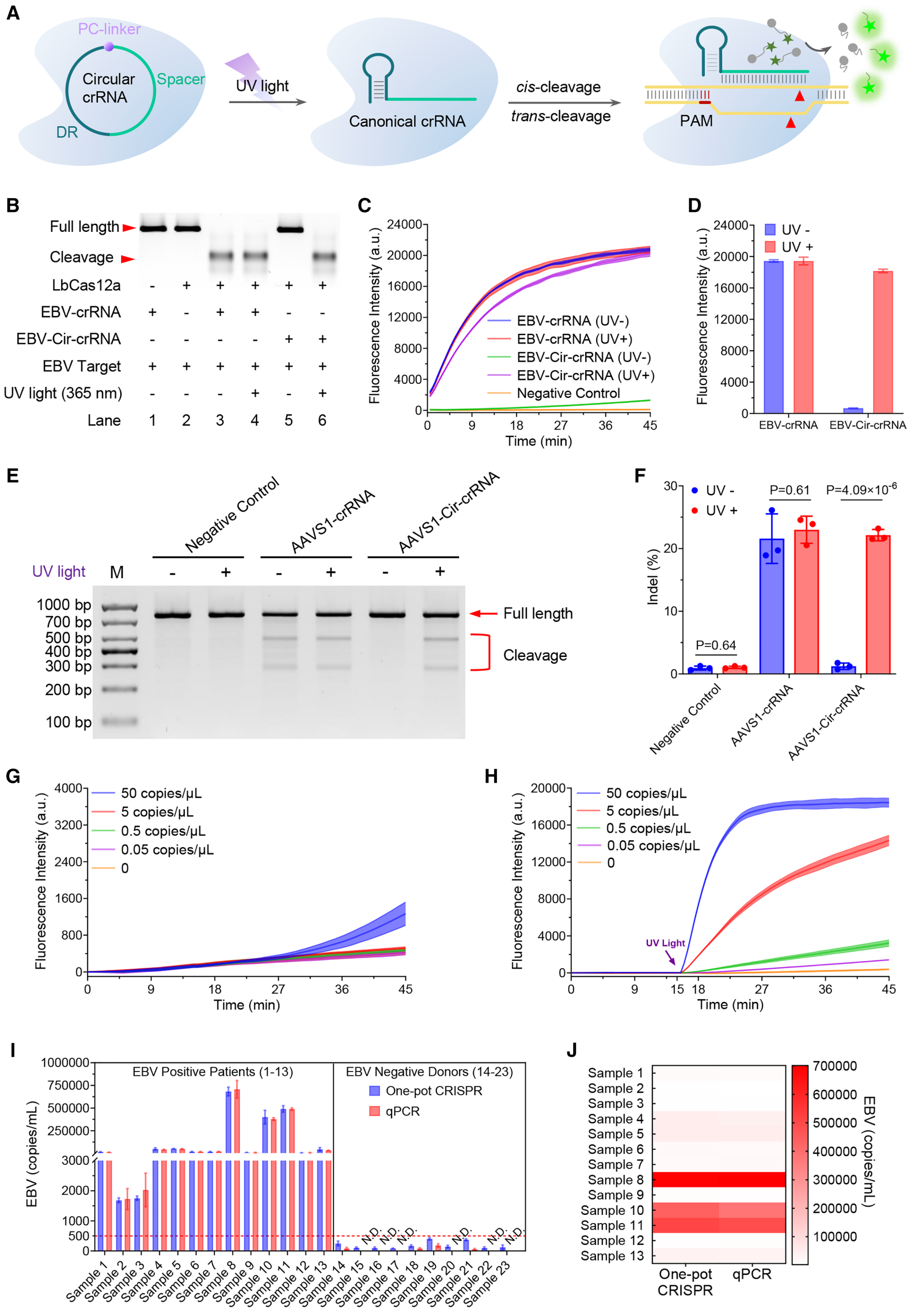

(legend on next page)

photocleavable (PC) linker into either the circular crRNA or universal circular DR. We hypothesize that these circular RNA designs effectively suppress LbCas12a activity, while ultraviolet (UV) light-mediated cleavage of the PC linker restores the native topology and reinstates the CRISPR-Cas12a cleavage function. This design promises to address the critical challenge of incompatibility between CRISPR systems and isothermal amplification of clinical trace nucleic acids, offering a robust platform for precise molecular control. Additionally, we propose introducing DNA modifications within the circular crRNA, such as deoxyuridine (dU). We hypothesize that circular crRNA with dU similarly inhibits LbCas12a activity, which can be restored by the corresponding base excision repair (BER) enzymes, such as uracil DNA glycosylase (UDG). Building upon this principle, we propose developing a series of BER enzyme detection methods by incorporating specific modified bases into the circular crRNA that correspond to target BER enzymes. This proposed circular system demonstrates several distinct advantages. The circular DR with a PC linker represents a universal platform requiring only a single design and synthesis, yet maintains versatility through its ability to pair with any spacer for target-specific detection. Furthermore, the inherent stability of circular RNA architecture surpasses that of linear RNA counterparts, significantly enhancing its suitability for long-term preservation and clinical implementation. Moreover, our system has the potential to expand the application of CRISPR-based detection beyond nucleic acids and offers a promising approach for precision gene editing by regulating the *cis*-cleavage activity of LbCas12a through the modulation of specific BER enzyme levels.

RESULTS

Circular crRNA with PC linker for UV-controlled regulation of LbCas12a cleavage activity

To investigate the inhibitory effects of circular crRNA incorporating a PC linker on both *cis*- and *trans*-cleavage activities of LbCas12a, along with its potential reactivation in photocontrolled isothermal amplification-CRISPR-Cas12a nucleic acid detection systems, we designed and synthesized an RNA sequence (EBV-Cir-crRNA precursor, Table S1) targeting Epstein-Barr virus (EBV) containing a commercially available PC linker (Figure 2A). The circular crRNA precursor was designed with a 5′-phosphate and the PC linker inserted in the middle (Figure S1A). Circularization of the crRNA precursor was achieved using T4 RNA ligase 1, followed by elimination of linear molecules through RNase R treatment. Successful circularization was confirmed using 18% denaturing polyacrylamide gel electrophoresis (PAGE) (Figure S1B). Furthermore, exposure of the PC linker-modified circular crRNA (EBV-Cir-crRNA) to UV light ($\lambda = 365$ nm, 35 W) resulted in rapid and efficient breakage of the PC linker. Complete breakage was achieved within 30 s and remained stable for 20 min (Figure S1C). These findings demonstrate the feasibility of adjusting the conformation of commercially available PC linker-modified circular crRNA through brief UV light exposure.

Subsequently, we investigated the inhibitory effect of circular crRNA with a PC linker on CRISPR-Cas12a *cis*-cleavage activity and explored the possibility of restoring this activity through UV irradiation-induced breakage of the PC linker. We synthesized a 357-bp target DNA sequence specific to the EBV (EBV target) for experimental purposes.[38] We compared the *cis*-cleavage activity of EBV-Cir-crRNA and conventional EBV crRNA (Table S1) using 2% agarose gel analysis under varying conditions. Our results showed that EBV-Cir-crRNA effectively inhibited CRISPR-Cas12a *cis*-cleavage activity due to its altered configuration (Figure 2B, lanes 1, 2, 3, and 5). This inhibition was completely reversed when the circular crRNA was subjected to short-duration UV irradiation, breaking the PC linker and restoring its canonical form (Figure 2B, lane 6). Notably, a 30-s UV irradiation had no impact on the system (Figure 2B, lanes 3, 4, and 6). These findings highlight the potential of circular crRNA with a PC linker as a versatile tool in controlling CRISPR-Cas12a cleavage activity.

Furthermore, we investigated the inhibitory effect of circular crRNA with a PC linker on the *trans*-cleavage activity of CRISPR-Cas12a and explored the potential for restoring this

Figure 2. Verification of photoinducible regulation of CRISPR-Cas12a *trans*- and *cis*-cleavage activities by circular crRNA with a commercially available PC linker under 30-s UV light exposure

(A) Schematic depiction reveals that circular crRNA with a PC linker can be disintegrated under UV light, reverting to conventional crRNA, further restoring the *trans*- and *cis*-cleavage activities of LbCas12a.

(B) A 2% agarose gel analysis verifies that circular crRNA with a PC linker can suppress LbCas12a *cis*-cleavage activity. Notably, 30 s under UV light can restore this activity.

(C) Fluorescence data showcase that circular crRNA can inhibit LbCas12a *trans*-cleavage activity. Again, exposure to UV light for 30 s is capable of bringing back this activity.

(D) The bar graph quantitatively compares the fluorescence values of classic and circular crRNAs at the 45-min time point under both UV irradiation and non-irradiation conditions, as shown in (C).

(E) T7E1 nuclease assays illustrate that the circular crRNA structure suppresses CRISPR-Cas12a gene editing at the AAVS1 locus in HEK293T cells in the absence of UV light. However, exposure to UV light induces the breakage of the circular crRNA, thereby restoring the functionality of CRISPR-Cas12a.

(F) A bar chart presents the percentage of indels (insertion or deletion mutations) observed under different conditions: negative control, canonical crRNA, and circular crRNA with and without UV exposure.

(G and H) Comparative analysis of detection limit between the conventional RPA-CRISPR-Cas12a one-pot detection method (G) and the circular crRNA-assisted RPA-CRISPR-Cas12a photocontrollable one-pot nucleic acid detection system we adopted for our current work (H) is depicted.

(I and J) The bar chart (I) and heatmap (J) illustrate the comparison between the traditional qPCR method and our current circular crRNA-assisted photocontrollable one-pot nucleic acid detection method in detecting EBV-infected blood samples in clinical settings. N.D., not detected. The red dashed line in (I) denotes the clinically established threshold of 500 copies/mL for EBV-DNA viral load. Values exceeding this threshold are indicative of EBV positivity in clinical diagnostics, as supported by the current literature and diagnostic standards.[52,53] This demarcation serves as a critical reference for interpreting EBV infection status in both experimental and clinical contexts.

In all panels, error bars represent the mean ± standard deviation (SD) ($n = 3$). $p > 0.05$ indicates no significant difference between groups.

activity by UV irradiation-induced cleavage of the PC linker. Comparative analysis of canonical EBV crRNA and EBV-Cir-crRNA with the addition of F-Q (Table S1, λ_{ex}: 494 nm, λ_{em}: 518 nm).[14,17] Our results (Figures 2C and 2D) showed that EBV-Cir-crRNA did not induce LbCas12a *trans*-cleavage activity without UV irradiation. However, under UV irradiation, breakage of the PC linker successfully restored CRISPR-Cas12a *trans*-cleavage activity. Short-term UV irradiation for 30 s had no significant effect on the system. These results provide strong evidence for the effective inhibition of LbCas12a cleavage activities by commercially available PC linker-modified circular crRNA. We demonstrated that a brief 30-s exposure to UV irradiation can successfully restore LbCas12a cleavage function.

To evaluate the photostability of circular crRNA containing the PC linker under experimental conditions, we performed stability assessments without specialized light protection, replicating standard laboratory illumination. Specifically, 100 ng EBV-Cir-crRNA was incubated in transparent 0.2-mL microcentrifuge tubes on ice for 0, 1, 2, 4, 8, 12, and 24 h. For comparative analysis, 100 ng UV-induced EBV-Cir-crRNA cleavage product served as a positive control for PC linker disruption. All samples were evaluated using 12% denaturing PAGE (150 V, 30 min) to assess PC linker integrity. The results confirmed the structural stability of circular RNA under normal laboratory lighting (Figure S1D). Furthermore, *trans*-cleavage activity assays comparing standard laboratory light-exposed and unexposed samples over 24 h showed no significant variation in LbCas12a activation, thereby validating the photostability of our circular RNA design (Figures S1E and S1F). To further investigate the enhanced environmental stability of our circular RNA (EBV-Cir-crRNA) relative to linear EBV crRNA, 100 ng of each RNA was incubated with 2 μL Dulbecco's Modified Eagle Medium supplemented with 10% fetal bovine serum (FBS) at 37°C for 0, 5, 10, 20, 40, and 60 min. Analysis by 12% denaturing PAGE (150 V, 30 min) revealed that linear crRNA underwent significant degradation within 10 min and was completely degraded by 40 min, whereas circular RNA retained its structural integrity throughout the 60-min incubation (Figure S1G). These findings highlight the superior stability of our circular RNA design, underscoring its potential advantages for commercial production, transportation, and practical applications compared to conventional linear RNA constructs.

CRISPR-Cas12a gene editing holds significant promise for the treatment of numerous human genetic diseases.[10,51,54] The adeno-associated virus integration site 1 (AAVS1), located on chromosome 19 of the human genome, is recognized as a "safe harbor" site.[55] This site is well validated for ensuring the intended functionality of integrated DNA fragments due to its open chromatin structure, which facilitates normal transcription of introduced genes. Importantly, the insertion of exogenous target sequences at the AAVS1 site has no known adverse effects on cellular function, making it a critical locus for *in vivo* gene editing studies.[56–58] In our study, we targeted the AAVS1 site with both a classical crRNA (AAVS1 crRNA, Table S1) and a circular crRNA (AAVS1-Cir-crRNA, Table S1). We systematically investigated the editing efficiency of these crRNAs in HEK293T cells under conditions with and without UV light (λ = 365 nm) exposure. First, we validated *in vitro* that AAVS1-Cir-crRNA-assisted CRISPR-Cas12a could not activate LbCas12a function in the absence of UV light. Conversely, after 30 s of UV exposure, the circular structure of AAVS1-Cir-crRNA reverted to its native state and reactivated LbCas12a function to a level comparable to that of classical crRNA (Figures S2A–S2C). Second, to evaluate the effects of UV light on cellular viability, HEK293T cells were exposed to UV irradiation for 0, 1, 2, 3, 5, and 10 min. Cell viability remained unchanged after 0–3 min of exposure, while a marginal decline was observed after 5 min (Figure S2E). To assess the genome-wide impact of UV irradiation, particularly its potential to induce DNA damage, a comet assay was performed. HEK293T cells were cultured under standard conditions and exposed to UV light (λ = 365 nm) for 1, 2, 3, 5, and 10 min, with the UV lamp positioned 15 cm above culture dishes (with lids) placed on ice. Untreated cells served as the negative control, and cells treated with 50 μM H_2O_2 for 1 h were used as the positive control. Post-exposure, cells were trypsinized, washed twice with PBS, and resuspended in PBS at a concentration of 1×10^6 cells/mL. The comet assay was conducted following the manufacturer's protocol (DNA Damage Comet Assay Kit, GBCBIO Technologies, catalog no. G7668). Fluorescence microscopy was used for image acquisition, and automated analysis was performed using the OpenComet plugin in ImageJ. The results revealed no significant DNA damage after 0–5 min of UV exposure; however, genome-wide damage was detected after 10 min (Figure S2D). Based on these findings, a mild condition of 365 nm UV irradiation for 3 min was selected for subsequent gene editing experiments, as it does not significantly compromise cell viability or induce detectable DNA damage. Then, we delivered AAVS1-Cir-crRNA and LbCas12a into HEK293T cells using the TransIT-X2 Dynamic Delivery System (Mirus Bio, catalog no. MIR 6000). The cells were then exposed to UV light (λ = 365 nm) for either 3 min or not, followed by another 42 h of culture. The T7 endonuclease I (T7E1) assay[59] was then used to measure the percentage of insertions or deletions (indels), which indicate non-homologous end joining events. Our results demonstrated that the classical crRNA achieved approximately 20% indels, independent of UV light exposure. In contrast, AAVS1-Cir-crRNA exhibited negligible editing activity without UV exposure, comparable to unedited samples. Upon UV light exposure, the editing efficiency of AAVS1-Cir-crRNA was comparable to that of classical crRNA (Figures 2E, 2F, S2F, and S2G). These findings confirm that UV light exposure can selectively regulate the CRISPR-Cas12a editing activity facilitated by circular RNA in living cells. This controlled gene editing strategy adds a valuable layer of precision, making it a promising tool for future *in vivo* genetic modification applications.

Development of a one-pot CRISPR-Cas12a nucleic acid detection system with PC linker-containing circular crRNA

Based on the exciting findings mentioned above, we developed a one-pot CRISPR-Cas12a nucleic acid detection system based on circular crRNA combined with recombinase polymerase amplification (RPA) technology.[60] After a 15-min isothermal amplification reaction at 37°C, 30 s of UV irradiation was used to initiate the CRISPR-Cas12a reaction, and fluorescence signals

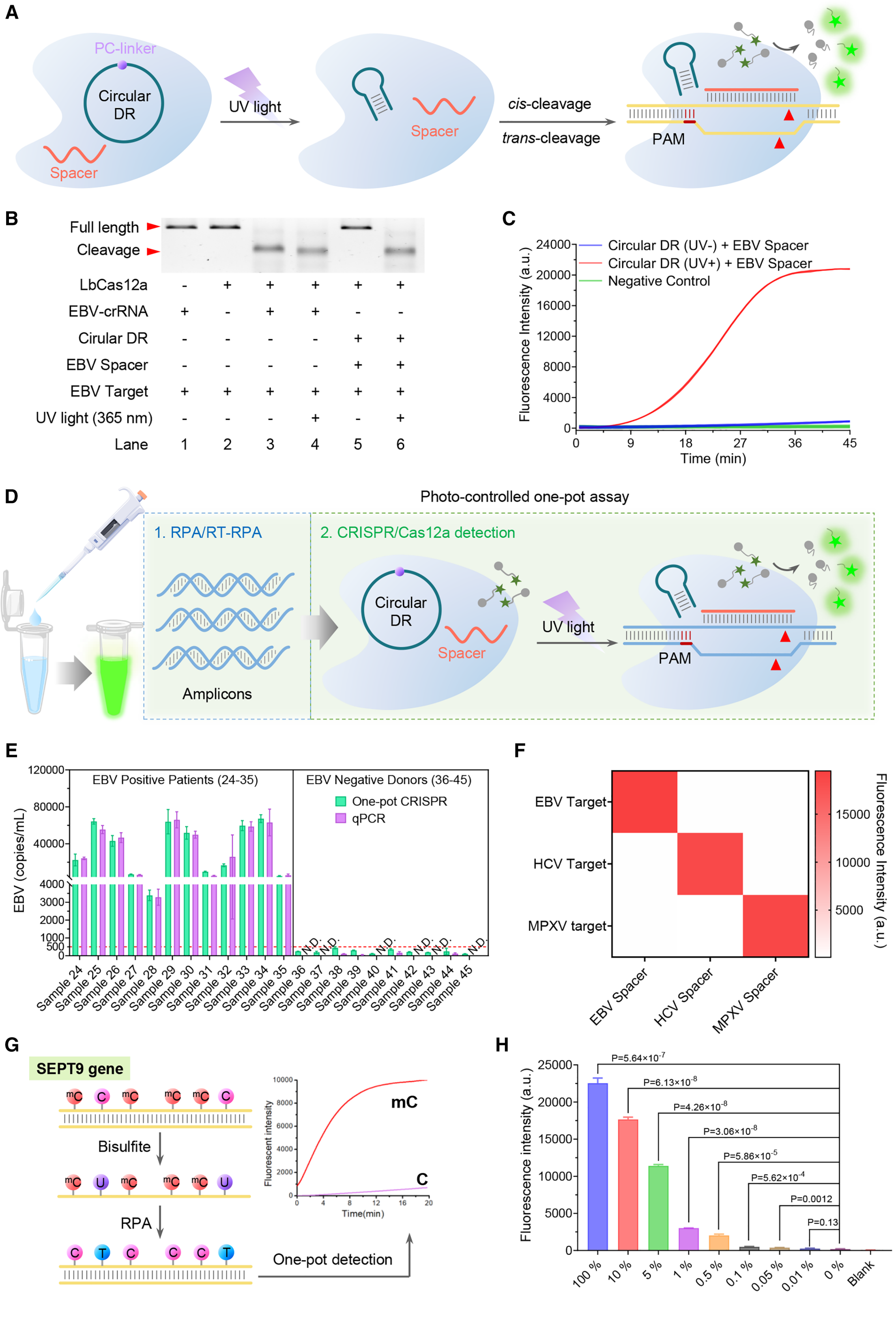

(legend on next page)

were recorded. Compared to canonical EBV crRNA (Figure 2G), our approach utilizing EBV-Cir-crRNA (Figure 2H) exhibited superior performance in detecting lower concentrations of the target DNA. The cleavage activity of LbCas12a degraded the EBV target during isothermal amplification, leading to the absence of detectable signals for low-concentration targets (Figure 2G; 0.05–5 copies/μL). Our system (Figure 2H) demonstrated a 100-fold improvement in detection limit compared to the conventional RPA-CRISPR-Cas12a system (Figure 2G).

Subsequently, we collected 13 blood samples from patients infected with EBV, as well as 10 samples from EBV^- donors, for further validation (Figures 2I and 2J). Worldwide, most individuals contract EBV in childhood, becoming lifelong asymptomatic carriers with low viral loads unless immunocompromised.[61] Clinically, EBV-DNA levels above 500 copies/mL define positive cases.[52,53] Initially, we extracted DNA from the blood samples and performed detection using the developed detection system with circular crRNA mentioned above. Utilizing the established standard curve by this system (Figures S3A and S3B), we calculated the copy number of the EBV in the clinic samples (Figures 2I and S3C). To evaluate performance, we compared our method with the gold standard qPCR (Figures S3D–S3F). In samples with high viral loads, our method exhibited detection consistency comparable to qPCR (Figures 2I and 2J). Notably, in samples with EBV levels below the clinical cutoff of 500 copies/mL, where qPCR often fails to provide precise quantification due to its higher detection limit, our method demonstrated superior sensitivity and reliable detection capabilities. This highlights its potential for enhanced accuracy in low-abundance viral load scenarios (Figures 2I, 2J, and S3). The PC linker-based crRNA circularization, while enabling trace nucleic acid detection, necessitates individual crRNA precursor re-circularization per target, substantially increasing costs. This highlights an urgent need for a universal circularization strategy applicable to diverse targets.

Development of a one-pot CRISPR-Cas12a nucleic acid detection system through a universal photocontrolled circular DR design

Building upon experimental evidence demonstrating that split crRNA retains full functionality in Cas12a systems,[30,62–64] we hypothesize that a circular DR with a PC linker could effectively inhibit both the *trans*- and *cis*-cleavage activities of Cas12a, with the potential for reactivation upon UV irradiation. That is, this circular DR region with a PC linker design could be universally employed, only modifying the spacer to detect different nucleic acid substrates (Figure 3A). To verify this hypothesis, we cyclized the DR sequence with a commercially available PC linker using the method previously described. The cyclization was confirmed by denaturing PAGE (Figure S4A). Experiments showed that 30-s UV light exposure could successfully restore the circular DR sequence to its canonical state (Figure S4B). Similarly, we have also demonstrated that the circular DR exhibits excellent stability and robust resistance to degradation (Figures S4C–S4F). We then put this discovery to test using a spacer targeting the EBV (EBV spacer, Table S1). The results indicate that when the circular DR is combined with the spacer, the enzymatic activity of CRISPR-Cas12a is completely abolished. However, upon exposure to UV light for 30 s, the circular DR returns to its usual form, restoring the activity of the LbCas12a (Figures 3B and 3C). We further augmented our system with RPA techniques, creating a one-pot testing system for EBV (Figure 3D). Our method was corroborated using 12 blood samples from EBV-infected and 10 blood samples from healthy individuals and the standard qPCR method (Figures 3E and S5). Results indicate our method can effectively be used for clinical detection of EBV.

To demonstrate the versatility of our design, we selected different target spacers like mpox (monkeypox) virus, hepatitis C virus, and EBV (Figures 3F and S4G–S4M). The results reveal that by using a universal circular DR with a PC linker and matching spacers, it is feasible to conduct one-pot isothermal amplification detection for different virus samples. Additionally, our method shows promise in early colon cancer detection. Previous research suggests that high methylation of the SEPT9 gene is a strong predictor of colon cancer.[65] We synthesized positive control samples with high methylation SEPT9 sequences and negative control samples with non-methylation sequences (Table S1).[37] After sodium bisulfite treatment, we used our established RPA-CRISPR-Cas12a system for detection. Figures 3G and 3H indicate that we have successfully detected high

Figure 3. Validation of the effective regulation of the LbCas12a *cis*- and *trans*-cleavage activities by circular DR with a PC linker under UV light exposure

(A) The schematic image reveals that UV light can break the circular DR containing a PC linker, which then reverts to its conventional form, subsequently restoring the *cis*- and *trans*-cleavage activities of LbCas12a with the help of different spacer.

(B) A 2% agarose gel analysis verifies the ability of the circular DR and spacer to inhibit LbCas12a *cis*-cleavage activity effectively. This activity can be restored following 30 s of UV light exposure.

(C) Fluorescence data demonstrate the inhibition of the *trans*-cleavage activity of LbCas12a by the circular DR with a PC linker. Again, a 30-s UV light exposure can restore this activity.

(D) The schematic image portrays the RPA/RT-RPA-CRISPR LbCas12a photocleavable one-pot nucleic acid detection system assisted by circular DR. Changing the replaceable spacer can enable its application for different nucleic acid target detection.

(E) Bar chart demonstrates a comparison between the traditional qPCR method and our current development, the circular DR-assisted RPA/RT-RPA-CRISPR-Cas12a photocleavable one-pot nucleic acid detection method for the detection of clinic EBV-infected blood samples.

(F) Heatmap analysis indicates that universal circular DR, when paired with different spacer, can be utilized for highly selective detection of various targets in a single separate way.

(G) Schematic representation of the current CRISPR-Cas12a-based assay for determining the methylation status of the SEPT9 gene, which is a nucleic acid biomarker in colorectal cancer. The feasibility of this method has been validated using synthetic hypermethylated (four sites) and non-methylated SEPT9 (Table S1).

(H) The detection results of different methylation proportions of the SEPT9 gene. All experiments were performed in triplicate. Data are presented as mean ± SD ($n = 3$). $p > 0.05$ indicates no significant difference between groups.

methylation of the SEPT9 gene, demonstrating that by changing the spacer, our method can detect not just multiple viral nucleic acid sequences, but also DNA modifications, such as 5-methylcytosine, that serve as colon cancer early detection biomarkers. Future endeavors will aim to expand the application of this system to detect a broader range of clinical samples.

Development of a BER enzyme detection platform based on DNA modification-containing circular crRNA-assisted CRISPR-Cas12a system

BER enzymes are involved in various biological processes, including DNA repair, gene expression regulation, and genetic recombination.[66] Additionally, DNA modifications are associated with specific BER enzymes, which interact with them.[66] DNA, as the primary genetic information repository, is susceptible to alterations caused by epigenetic processes and external factors like chemicals, radiation, and metabolic byproducts.[67,68] These alterations, such as base modifications, can spontaneously occur in DNA and affect cellular activities.[69] Recently, BER enzymes that interact with these DNA modifications have gained significant attention.[70] For example, UDG is a BER enzyme that specifically identifies and repairs dU, which is naturally present in genomic DNA.[71] The apurinic/apyrimidinic (AP) site generated by UDG from dU can be eliminated through alpha or beta elimination,[72] leading to a DNA strand break.[73] This BER process maintains DNA integrity and stability by preventing incorrect base pair integration.[66] Moreover, UDG plays a crucial role in detecting viral infections[74,75] and can be used as a biomarker for colon cancer.[76] Therefore, the development of an efficient and user-friendly method for UDG detection is essential.

Based on the findings presented above, it is proposed that the incorporation of specific DNA modifications into circular crRNA could inhibit the activity of CRISPR-Cas12a. This could be achieved by using a specific BER enzyme to selectively cleave the DNA modification-containing crRNA, restore its canonical conformation, and thereby reactivate CRISPR-Cas12a function. This would result in the digestion of the ssDNA fluorescent reporter (e.g., F-Q), allowing us to quantify BER enzyme levels based on the increase in fluorescence.

In this study, we selected dU as the model system for testing (Figure 4A). We designed a circular crRNA with a universal 19-nt DR region, an 18-nt RNA spacer targeting EBV, and a 7-nt DNA segment with dU strategically positioned in the center. Previous studies have shown that an 18-nt RNA spacer can greatly preserve the activity of CRISPR-Cas12a,[54] and UDG has been found to efficiently recognize dU within a DNA chain of at least 7 bases.[77] We performed cyclization of the precursor crRNA containing dU using T4 RNA ligase 1. Residual starting molecules and linear ligation by-products were removed using RNase R, generating a circular crRNA containing dU (Figures S6A and S6B). This dU-containing crRNA was then treated with UDG in a CRISPR-Cas12a working buffer containing spermidine. Our observations showed that UDG efficiently removes the uracil base, creating an AP site. With the assistance of spermidine, an addition reaction occurs at the AP site, followed by beta elimination, resulting in the generation of a beta-elimination product[72] that reverts the circular crRNA back to its canonical form (Figures S6D and S6E).

Next, we developed a UDG detection platform using CRISPR-Cas12a, circular crRNA with dU targeting EBV, EBV target, and F-Q. Validation experiments confirmed that the dU-containing circular crRNA could suppress CRISPR-Cas12a activity, and the dU-specific BER enzyme UDG could selectively restore CRISPR-Cas12a activity by cleaving the dU-containing circular crRNA (Figures S6F–S6H). To further optimize the system, we investigated the influence of the initial concentration of the reaction substrate (EBV target) on the detection performance. Under consistent experimental conditions, we tested UDG detection limits at EBV target concentrations ranging from 40 nM, 20 nM, 10 nM, 5 nM, and 2.5 nM to 1.25 nM. Our results revealed that the detection limit for UDG remained stable at approximately 1.72–1.92 U/L, when the EBV target concentration was between 40 and 5 nM (Figures 3B and S7). However, when the EBV target concentration decreased to 2.5 nM, the detection limit deteriorated to 4.82 U/L (Figure S7D). Based on these findings, we selected 5 nM as the optimal initial concentration of the reaction substrate (EBV target) for our system. We then conducted UDG fluorescence detection experiments by systematically diluting UDG in the range of 0–500 U/L (Figures 4B and S8A). A strong linear relationship was observed between fluorescence and UDG concentration within the 0–125 U/L range (Figure 4B). Control experiments with bovine serum albumin, AP endonuclease 1 (APE1), human 8-oxoguanine DNA N-glycosylase 1 (hOGG1), UDG, and their mixtures (Figure S8B) demonstrated the specificity of the UDG detection method, with only UDG or the UDG-containing mixture opening the circular crRNA and restoring CRISPR-Cas12a activity, leading to increased fluorescence.

We also cultured various cancer cells (HCT116, SW480, MCF-7, HeLa), normal cells (human colon mucosal epithelial cells NCM460), and HEK293T to examine UDG concentrations. The UDG concentrations in MCF-7 and HeLa cells were found to be 850–880 U/L (Figure S8E), consistent with a previous report.[78] The UDG concentrations in colorectal cancer cells (HCT116: 670.5 U/L, SW480: 519.6 U/L) were significantly higher than those in the non-cancer cell line HEK293T (17.9 U/L) and normal colon mucosal epithelial cells NCM460 (14.4 U/L) (Figure S8E). To validate our experiments, a commercial UDG ELISA kit was employed to evaluate the UDG content[78] in the same cultured cells. The high level of consistency and comparability observed in the results serves as strong validation for the reliability of our methodology (Figures 4D and S8D–S8F). Additionally, uracil glycosylase inhibitor (UGI) was used as an inhibitor of UDG, and fluorescence detection in cell protein extracts was conducted with and without UGI. The addition of UGI resulted in a noticeable decrease in the fluorescence detection signal of UDG in cancer cells, bringing the fluorescence intensity of cancer cells with UGI to a level similar to that of HEK293T and NCM460 cells (Figure 4C). This secondary validation confirmed the high sensitivity of our UDG detection method. The half-maximal inhibitory concentration value for UGI in our method was calculated to be 0.087 U/mL (Figure S8C), consistent with the literature record of 0.07 U/mL.[78] In addition to nuclear protein extracts, we also quantified UDG levels in lysates from diverse cell lines. Our findings revealed that the fluorescence intensity of UDG detection was significantly higher in HCT116 and

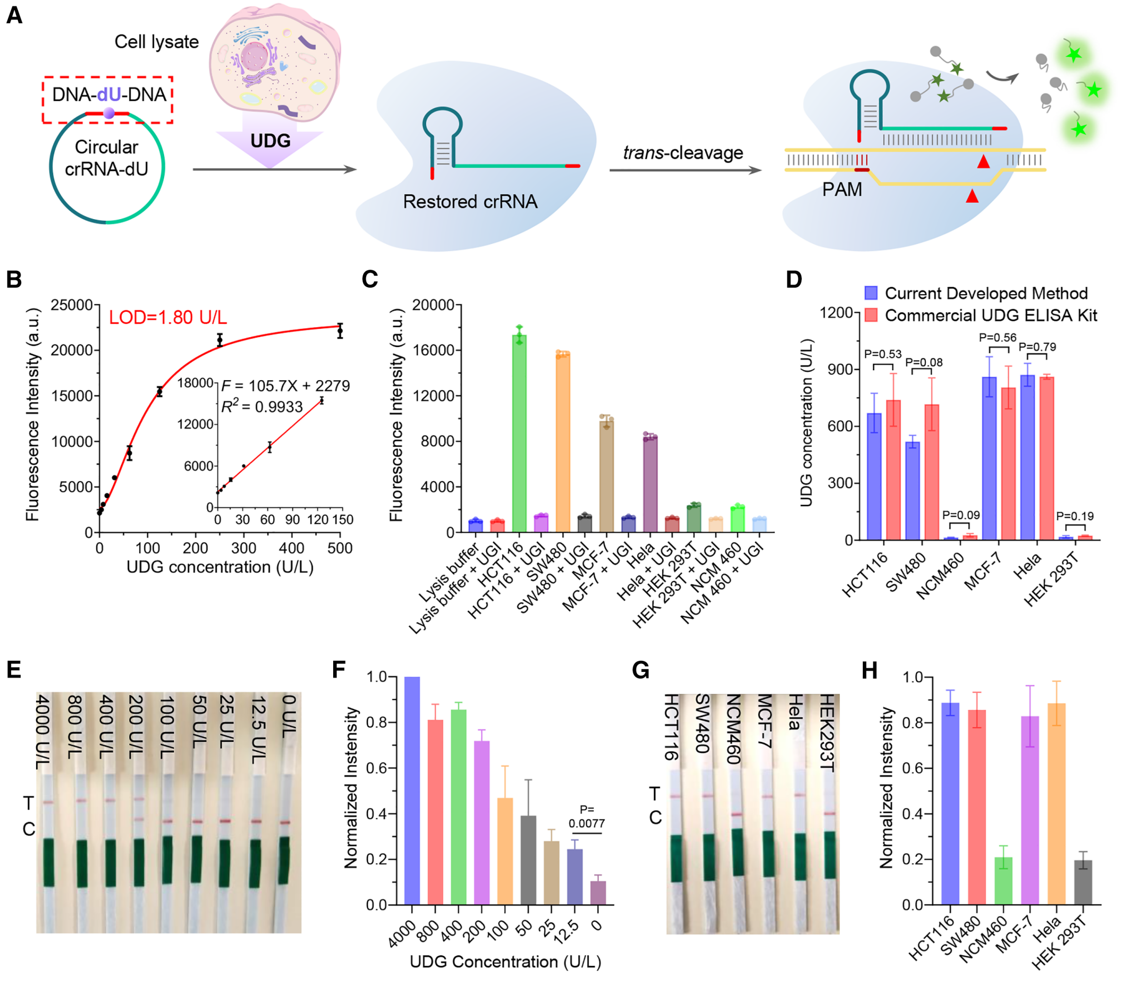

Figure 4. Development of a DNA modification-containing circular crRNA-assisted CRISPR-Cas12a system for BER enzyme detection and regulation of CRISPR-Cas12a gene editing by using BER enzymes

(A) A conceptual diagram of the DNA modification-containing circular crRNA-assisted CRISPR-Cas12a system utilized for BER enzyme detection. BER enzymes can selectively act on specific DNA modifications, and circular crRNA can be opened to revert to its conventional form, thereby igniting LbCas12a *trans*-cleavage activity and releasing a fluorescent signal. This procedure is exemplified by UDG enzymes and dU.

(B) Fluorescence data of detection of various UDG enzyme concentrations by this strategy, depicting the detection limit and quantitative relationship.

(C) Comparison with and without UGI in different cell line protein extracts validates the uniqueness and effectiveness of UDG detection in the system.

(D) Comparative bar graph for levels of UDG in different cell lines detected by current developed method and commercial UDG ELISA kit.

(E) The paper strip assay was conducted to quantify the concentrations of UDG, and the results were documented using a mobile phone camera.

(F) ImageJ software was used to analyze the grayscale bands in the images obtained from the paper strip assay for various concentrations of UDG.

(G) The paper strip assay was employed for visual UDG detection of cell protein extracts from HCT116, SW480, NCM460, HeLa, and HEK293T cell lines.

(H) ImageJ analysis was carried out on the grayscale bands from the visual detection results of the paper strip assay for the aforementioned cell lines. Error bars are mean ± SD ($n = 3$). Statistical significance was determined using a threshold of $p < 0.05$, where $p > 0.05$ indicates no significant difference and $p < 0.05$ denotes a statistically significant difference.

SW480 cells compared to the non-cancerous cell line NCM460 (Figure S8G). These results provide strong validation of the effectiveness and success of our UDG detection method based on circular crRNA containing dU modifications.

To demonstrate the broad applicability and developmental value of this approach, we have enhanced the method and integrated it with a commercial test strip to develop a point-of-care testing (POCT) system for detecting BER enzymes (Figure S8H). This test strip is based on an immunochromatographic assay system, where the original F-Q has been replaced with an FAM-biotin DNA reporter (Figure S9H; Table S1), a DNA probe labeled with biotin at one end and FAM at the other end. In the absence of UDG, LbCas12a lacks *trans*-cleavage activity, leaving the FAM-biotin DNA reporter intact. When the strip is immersed in this solution, the intact reporter binds to colloidal gold conjugated with anti-FAM antibodies, migrating to the control (C) line and becoming fully captured by avidin, resulting in a visible band. In the presence of UDG, the circular RNA is opened, activating the *trans*-cleavage activity of LbCas12a, which cleaves the FAM-biotin DNA reporter. The cleaved DNA

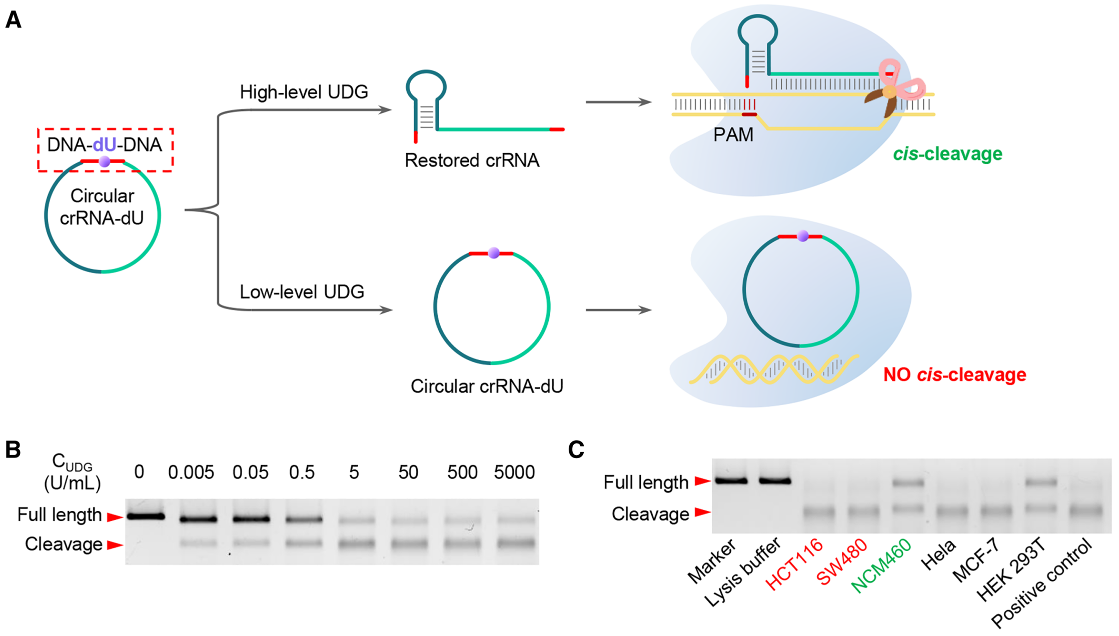

Figure 5. Precise regulation of CRISPR-Cas12a *cis*-cleavage through the use of BER enzymes corresponding to DNA modifications on circular crRNA

(A) Schematic representation of the regulation of CRISPR-Cas12a *cis*-cleavage through different concentrations of UDG.

(B) A 2% agarose gel showing variable UDG concentrations (from 0 to 5,000 U/mL) markedly affects the gene editing capabilities of this dU-containing circular crRNA-assisted CRISPR-Cas12a system.

(C) *In vitro* regulation of this system's *cis*-cleavage abilities by protein extracts from different cell lines. Relative to normal human colonic epithelial cells, highlighted in green, this system possesses stronger *cis*-cleavage abilities in high UDG expression colon cancer cell line protein extracts, highlighted in red.

fragment with FAM but without biotin binds to the colloidal gold conjugated with anti-FAM antibodies, migrates to the C line, but is not captured by the avidin due to the absence of biotin, ultimately reaching the test (T) line where it is captured by immobilized anti-*anti*-FAM antibodies, resulting in a visible band at the T line. We tested this system using UDG solutions with known concentrations ranging from 0 to 4,000 U/L (Figure 4E). The test strip results enabled us to detect UDG concentrations as low as 12.5 U/L (Figure 4F). As the UDG concentration increased, the signal intensity at the T line strengthened, while that at the C line weakened. By analyzing the grayscale values of the test strip images via ImageJ, we were able to achieve a rough quantification of UDG levels (Figure 4F). To evaluate the reliability of our method, we tested protein extracts from different cell lines. Notably, cell lines with high UDG expression such as HCT116, SW480, MCF-7, and HeLa effectively activated the LbCas12a system, displaying stronger visible bands on the T line. In contrast, cell lines with low UDG expression like NCM460 and HEK293T exhibited weaker bands on the T line (Figures 4G and 4H). This innovative and visual detection method is quickly responsive and reliable in various environments, successfully eliminating the need for complex instrumentation and simplifying the existing detection process, thus providing an efficient pathway for high-performance POCT.

To demonstrate the programmability and versatility of our design, we developed a circular crRNA containing tetrahydrofuran (THF; Figure S9H), a modification, for the detection of APE1. APE1 is known to be highly selective for breaking THF.[79] We designed a circular crRNA with a universal 19-nt DR region, an 18-nt RNA spacer, and 5-nt DNA bases with THF incorporated (Figure S8I). The circular crRNA containing THF was synthesized and confirmed (Figure S6C). We also demonstrated that APE1 could efficiently open the circular crRNA containing THF, and the crRNA with THF could be used to detect APE1 levels (Figures S8J–S8L). This highlights the potential of our design paradigm to be extended to other modified bases and enzymes, such as 8-oxo-guanine and hOGG1.[67,68]

Regulation of CRISPR-Cas12a cleavage activity through the use of BER enzyme corresponding to DNA modification

Based on the recognition ability of BER enzymes for DNA modifications and the abundance of certain BER enzymes in cancer cells, we proposed the development of a regulatory system for CRISPR-Cas12a *cis*-cleavage activity using BER enzymes. Initial testing was performed using crRNA containing dU, as shown in Figure 5A. In systems with high UDG expression, the circular crRNA containing dU was broken down due to UDG treatment, resulting in a reduction of the inhibitory effect on LbCas12a and an enhancement of its *cis*-cleavage capability. Conversely, in systems with low or no UDG expression, the circular crRNA containing dU remained intact, leading to the inhibition of the *cis*-cleavage capacity of LbCas12a.

To examine UDG's effect on the *cis*-cleavage of CRISPR-Cas12a, we tested its EBV-targeting activity at varying

concentrations. As shown in Figure 5B, CRISPR-Cas12a showed no *cis*-cleavage without UDG, but activity increased progressively with higher UDG levels. Subsequently, we extracted proteins from both colorectal cancer cell lines HCT116 and SW480, known for high UDG expression, and from HEK293T and human colon mucosal epithelial cells NCM460 known for low UDG expression (Figure 4D). Through *in vitro* verification, we affirmed that extracted protein from HCT116 and SW480 (colorectal cancer cells) gave the developed CRISPR-Cas12a system a notably stronger *cis*-cleavage ability (Figure 5C). On the contrary, protein extracts from NCM460 (a normal human colon mucosal epithelial cell line) resulted in a comparatively low *cis*-cleavage performance (Figure 5C). This assay verified the feasibility of our approach to regulate LbCas12a *cis*-cleavage through the use of BER enzymes acting on DNA modification-containing circular crRNA. This study contributes to the development of more precise and controlled *cis*-cleavage techniques. By utilizing BER enzymes in the regulation of LbCas12a *cis*-cleavage, we created a potential strategy for enhancing editing precision and specificity. Future endeavors will focus on more in-depth studies in cellular and animal models. Our design, based on the DNA modification-containing circular crRNA-assisted CRISPR-Cas12a system, holds great promise for future gene therapy applications, particularly in cell types exhibiting differential BER enzyme levels.

DISCUSSION

The CRISPR-Cas12a system has emerged as a transformative technology in medical nucleic acid detection, offering remarkable clinical utility in emergency scenarios and disease outbreaks.[20,39] Its advantages include minimal sample requirements, isothermal operation at 37°C within 30–45 min, and independence from specialized equipment or personnel.[21,34] However, challenges persist in integrating isothermal amplification with CRISPR-Cas12a for trace nucleic acid detection, particularly in one-pot assays, alongside difficulties in precise Cas12a regulation and the system's current limitation to nucleic acid targets.[38]

In this study, we systematically evaluated various engineered circular RNA, including full-length and split systems, for their ability to modulate LbCas12a activity. Our approach introduces a universal circular DR with a PC linker, compatible with diverse spacers for different target detection, significantly reducing cost and complexity while enhancing versatility. Compared to linear RNAs, our circular RNAs demonstrated superior stability, maintaining integrity after 60-min incubation in 10% FBS or 24-h exposure to lab routine light, underscoring their robust practical utility (Figures S1D–S1G and S4C–S4F). Furthermore, our circularization strategy extends beyond nucleic acid detection, enabling non-nucleic acid target detection through introducing DNA modifications on circular RNA, thereby expanding potential applications in gene therapy and enzyme detection.

A key innovation involves integrating circular RNA design with split crRNA systems. While split crRNAs showed slightly higher detection limits (1.17 pM; Figures S9C and S9D) compared to full-length crRNA (0.10 pM; Figures S9A and S9B) in stand-alone CRISPR assays, performance converged in one-pot RPA-CRISPR assays (12.72 vs. 14.04 copies/mL; Figures 5B and S3B). This performance parity can be attributed to the temporal inhibition of Cas protein activity by circular crRNA or DR during isothermal amplification, which facilitates effective target amplification even at low initial concentrations. Consequently, in the RPA-CRISPR one-pot system, the detection limit becomes less dependent on the efficiency of the CRISPR system, whether using full-length or split constructs. These findings align with recent work by Cheng et al., which demonstrated that heparin sodium reduces Cas12a efficiency while enhancing low-concentration target detection in one-pot assays, published during our manuscript review period.[80] Our universal circular DR design, requiring only a single synthesis, holds promise for application to diverse targets.

Notably, our optimized system demonstrated minimal *trans*-cleavage activity with spacer alone (100 nM; Figure S9E), remaining negligible compared to DR + spacer complexes. Even at 5 μM, no significant spacer-induced *cis*-cleavage was observed (Figure S9F), aligning with observations by Shebanova et al.,[62] reinforcing the crucial role of DR + spacer complexes in driving LbCas12a activity. While our current focus is on developing universal circular DR, we acknowledge the potential of spacer circularization for precise Cas protein activity control, with circular DR + circular spacer combinations showing particularly low background signals (Figure S9G).

We further introduced DNA modifications in circular crRNAs, effectively inhibiting LbCas12a activity while enabling non-nucleic acid target detection. In UDG detection, our method outperformed commercial ELISA kits, offering superior specificity by eliminating antibody dependence and associated cross-reactivity risks. The streamlined protocol, leveraging UDG's inherent specificity for dU in circular crRNA, enables rapid detection with minimal sample preparation, significantly reducing time and cost. This modular platform technology allows for the detection of various DNA modification-related enzymes through simple target-specific DNA modification substitutions, contrasting with ELISA's requirement for target-specific antibody development.

Additionally, we propose a potential precise regulation of the CRISPR-Cas12a *cis*-cleavage strategy based on the expression of BER enzymes. Our *in vitro* experiments have revealed that higher expression of BER enzymes, such as UDG, in colorectal cancer cell lines leads to enhanced *cis*-cleavage activity compared to normal colon mucosal epithelial cell line with lower UDG expression. Given the varying expression levels of enzymes in specific cells, this design, which is matched one-to-one with DNA modifications and BER enzymes, holds potential for future clinical applications in gene therapy. These findings provide perspectives and opportunities for clinical nucleic acid detection and future gene editing.

In summary, this study demonstrates improved regulation of the CRISPR-Cas12a system through circular RNA designs and establishes a platform for BER enzyme detection. Using universal circular DR with a PC linker, we developed a versatile, simple, and cost-effective trace nucleic acid detection system compatible with isothermal amplification. The introduction of DNA modification on circular crRNAs enables BER enzyme detection and shows potential for future gene therapy applications. These findings contribute to clinical diagnostics and gene editing technologies.

Limitations of the study

The light-triggerable circular RNA system is dependent on external UV light activation, which may restrict its applicability in resource-limited settings and necessitates additional UV light-protection measures and operator safety protocols. While DNA modifications on circular crRNA facilitate CRISPR-Cas12a regulation through BER enzymes, and this mechanism has been validated *in vitro*, the potential impact of cell-to-cell variability in BER enzyme expression on *in vivo* gene editing efficacy remains unexplored. Commercially available UDG exhibited greater DNA cleavage variability in *in vitro* assays compared to endogenously expressed UDG in cellular extracts, highlighting the significant challenges this strategy may encounter within the complex intracellular environment. Insufficiently distinct BER enzyme expression levels may compromise the system's regulatory efficacy. Although RNA circularization enhances the regulation of Cas12a activity and expands its utility for non-nucleic acid targets, the process of RNA circularization itself presents technical and cost-related challenges. Advances in RNA circularization technology are anticipated to address these limitations in the future.

RESOURCE AVAILABILITY

Lead contact

Further information and requests for resources and reagents should be directed to and will be fulfilled by the lead contact, Chaoxing Liu (liuchx69@mail.sysu.edu.cn).

Materials availability

This study did not generate new unique reagents.

Data and code availability

- The uncropped raw gel images associated with this study have been deposited in Figshare and are publicly accessible under the following weblink: https://doi.org/10.6084/m9.figshare.28887740.
- This paper does not report original code.
- Any additional information required to reanalyze the data reported in this paper is available from the lead contact upon request.

ACKNOWLEDGMENTS

This work was supported by the National Natural Science Foundation of China (grant no. 22307150), the Shenzhen Medical Research Fund (grant nos. A2303012 and A2401010), the Guangdong Basic and Applied Basic Research Foundation (grant no. 2024A1515012319), and the Shenzhen Science and Technology Program (grant nos. JCYJ20230807110315032 and JCYJ20240813150427036). Human blood samples were collected and provided by the Seventh Affiliated Hospital, Sun Yat-sen University, with protocols approved by the ethics committee at the Seventh Affiliated Hospital, Sun Yat-sen University (KY-2024-009-02 and KY-2023-117-01).

AUTHOR CONTRIBUTIONS

C.L., J.W., and W.Z. contributed to conceptualization, supervision, and study design. J.W., W.Z., W.L., Q.X., and Z.Z. performed the experiments and data analysis. C.L., J.W., and W.Z. verified the underlying data and wrote the manuscript. All authors reviewed, revised, and approved the final version of the manuscript.

DECLARATION OF INTERESTS

The authors declare no competing interests.

STAR★METHODS

Detailed methods are provided in the online version of this paper and include the following:

- KEY RESOURCES TABLE
- EXPERIMENTAL MODEL AND STUDY PARTICIPANT DETAILS
  - Cell culture
  - Patient material & consent
- METHOD DETAILS
  - Preparation and validation of circular RNA
  - Determining the cleavage time of the PC linker in circular RNA
  - Photocontrolled AAVS1-Cir-crRNA assisted CRISPR-Cas12a gene editing experiment
  - T7 endonuclease I (T7EI) assay for evaluating gene editing efficiency
  - Traditional RPA-Cas12a one-pot nucleic acid detection for EBV
  - Photocontrolled one-pot method for Clinical EBV sample analysis using EBV-Cir-crRNA or circular DR
  - Detection of different UDG enzyme levels in various cell lines using circular-crRNA-dU assisted CRISPR-Cas12 system
  - Detection of nuclear UDG enzyme activity in various cell lines using lateral flow assay strips
- QUANTIFICATION AND STATISTICAL ANALYSIS
  - Statistical analysis and data interpretation

SUPPLEMENTAL INFORMATION

Supplemental information can be found online at https://doi.org/10.1016/j.crmeth.2025.101076.

Received: September 13, 2024
Revised: May 6, 2025
Accepted: May 16, 2025
Published: June 16, 2025

REFERENCES

1. Barrangou, R., Fremaux, C., Deveau, H., Richards, M., Boyaval, P., Moineau, S., Romero, D.A., and Horvath, P. (2007). CRISPR Provides Acquired Resistance Against Viruses in Prokaryotes. Science *315*, 1709–1712.
2. Barrangou, R., and Marraffini, L.A. (2014). CRISPR-Cas Systems: Prokaryotes Upgrade to Adaptive Immunity. Mol. Cell *54*, 234–244.
3. Zetsche, B., Gootenberg, J.S., Abudayyeh, O.O., Slaymaker, I.M., Makarova, K.S., Essletzbichler, P., Volz, S.E., Joung, J., van der Oost, J., Regev, A., et al. (2015). Cpf1 Is a Single RNA-Guided Endonuclease of a Class 2 CRISPR-Cas System. Cell *163*, 759–771.
4. Fonfara, I., Richter, H., Bratovič, M., Le Rhun, A., and Charpentier, E. (2016). The CRISPR-associated DNA-cleaving enzyme Cpf1 also processes precursor CRISPR RNA. Nature *532*, 517–521.
5. Swarts, D.C., van der Oost, J., and Jinek, M. (2017). Structural basis for guide RNA processing and seed-dependent DNA targeting by CRISPR-Cas12a. Mol. Cell *66*, 221–233.e4.
6. Stella, S., Alcón, P., and Montoya, G. (2017). Structure of the Cpf1 endonuclease R-loop complex after target DNA cleavage. Nature *546*, 559–563.
7. Shmakov, S., Abudayyeh, O.O., Makarova, K.S., Wolf, Y.I., Gootenberg, J. S., Semenova, E., Minakhin, L., Joung, J., Konermann, S., Severinov, K., et al. (2015). Discovery and Functional Characterization of Diverse Class 2 CRISPR-Cas Systems. Mol. Cell *60*, 385–397.
8. Ling, X., Chang, L., Chen, H., Gao, X., Yin, J., Zuo, Y., Huang, Y., Zhang, B., Hu, J., and Liu, T. (2021). Improving the efficiency of CRISPR-Cas12a-based genome editing with site-specific covalent Cas12a-crRNA conjugates. Mol. Cell *81*, 4747–4756.e7.

9. Zhang, X., Wang, X., Lv, J., Huang, H., Wang, J., Zhuo, M., Tan, Z., Huang, G., Liu, J., Liu, Y., et al. (2023). Engineered circular guide RNAs boost CRISPR/Cas12a- and CRISPR/Cas13d-based DNA and RNA editing. Genome Biol. *24*, 145.
10. Bin Moon, S., Lee, J.M., Kang, J.G., Lee, N.E., Ha, D.I., Kim, D.Y., Kim, S. H., Yoo, K., Kim, D., Ko, J.H., and Kim, Y.S. (2018). Highly efficient genome editing by CRISPR-Cpf1 using CRISPR RNA with a uridinylate-rich 3'-overhang. Nat. Commun. *9*, 3651.
11. Nguyen, L.T., Smith, B.M., and Jain, P.K. (2020). Enhancement of trans-cleavage activity of Cas12a with engineered crRNA enables amplified nucleic acid detection. Nat. Commun. *11*, 4906.
12. Rossetti, M., Merlo, R., Bagheri, N., Moscone, D., Valenti, A., Saha, A., Arantes, P.R., Ippodrino, R., Ricci, F., Treglia, I., et al. (2022). Enhancement of CRISPR/Cas12a trans-cleavage activity using hairpin DNA reporters. Nucleic Acids Res. *50*, 8377–8391.
13. Li, S.Y., Cheng, Q.X., Liu, J.K., Nie, X.Q., Zhao, G.P., and Wang, J. (2018). CRISPR-Cas12a has both cis- and trans-cleavage activities on single-stranded DNA. Cell Res. *28*, 491–493.
14. Chen, J.S., Ma, E., Harrington, L.B., Da Costa, M., Tian, X., Palefsky, J.M., and Doudna, J.A. (2018). CRISPR-Cas12a target binding unleashes indiscriminate single-stranded DNase activity. Science *360*, 436–439.
15. Mohammad, N., Katkam, S.S., and Wei, Q. (2022). A Sensitive and Non-optical CRISPR Detection Mechanism by Sizing Double-Stranded λ DNA Reporter. Angew. Chem. Int. Ed. *61*, e202213920.
16. Mohammad, N., Talton, L., Hetzler, Z., Gongireddy, M., and Wei, Q. (2023). Unidirectional trans-cleaving behavior of CRISPR-Cas12a unlocks for an ultrasensitive assay using hybrid DNA reporters containing a 3' toehold. Nucleic Acids Res. *51*, 9894–9904.
17. Li, S.-Y., Cheng, Q.-X., Wang, J.-M., Li, X.-Y., Zhang, Z.-L., Gao, S., Cao, R.-B., Zhao, G.-P., and Wang, J. (2018). CRISPR-Cas12a-assisted nucleic acid detection. Cell Discov. *4*, 20.
18. Broughton, J.P., Deng, X., Yu, G., Fasching, C.L., Servellita, V., Singh, J., Miao, X., Streithorst, J.A., Granados, A., Sotomayor-Gonzalez, A., et al. (2020). CRISPR-Cas12-based detection of SARS-CoV-2. Nat. Biotechnol. *38*, 870–874.
19. Dai, Y., Somoza, R.A., Wang, L., Welter, J.F., Li, Y., Caplan, A.I., and Liu, C.C. (2019). Exploring the Trans-Cleavage Activity of CRISPR-Cas12a (cpf1) for the Development of a Universal Electrochemical Biosensor. Angew. Chem. Int. Ed. *58*, 17399–17405.
20. Dronina, J., Samukaite-Bubniene, U., and Ramanavicius, A. (2022). Towards application of CRISPR-Cas12a in the design of modern viral DNA detection tools. J. Nanobiotechnol. *20*, 1–15.
21. Mao, Z., Chen, R., Wang, X., Zhou, Z., Peng, Y., Li, S., Han, D., Li, S., Wang, Y., Han, T., et al. (2022). CRISPR/Cas12a-based technology: A powerful tool for biosensing in food safety. Trends Food Sci. Technol. *122*, 211–222.
22. Shi, K., Xie, S., Tian, R., Wang, S., Lu, Q., Gao, D., Lei, C., Zhu, H., and Nie, Z. (2021). A CRISPR-Cas autocatalysis-driven feedback amplification network for supersensitive DNA diagnostics. Sci. Adv. *7*, eabc7802.
23. Ma, P., Meng, Q., Sun, B., Zhao, B., Dang, L., Zhong, M., Liu, S., Xu, H., Mei, H., Liu, J., et al. (2020). MeCas12a, a Highly Sensitive and Specific System for COVID-19 Detection. Adv. Sci. *7*, 2001300.
24. Ooi, K.H., Liu, M.M., Tay, J.W.D., Teo, S.Y., Kaewsapsak, P., Jin, S., Lee, C.K., Hou, J., Maurer-Stroh, S., Lin, W., et al. (2021). An engineered CRISPR-Cas12a variant and DNA-RNA hybrid guides enable robust and rapid COVID-19 testing. Nat. Commun. *12*, 1739.
25. Hu, M., Qiu, Z., Bi, Z., Tian, T., Jiang, Y., and Zhou, X. (2022). Photocontrolled crRNA activation enables robust CRISPR-Cas12a diagnostics. Proc. Natl. Acad. Sci. USA *119*, e2202034119.
26. Chen, Y., Shi, Y., Chen, Y., Yang, Z., Wu, H., Zhou, Z., Li, J., Ping, J., He, L., Shen, H., et al. (2020). Contamination-free visual detection of SARS-CoV-2 with CRISPR/Cas12a: A promising method in the point-of-care detection. Biosens. Bioelectron. *169*, 112642.
27. Park, J.S., Hsieh, K., Chen, L., Kaushik, A., Trick, A.Y., and Wang, T.H. (2021). Digital CRISPR/Cas-Assisted Assay for Rapid and Sensitive Detection of SARS-CoV-2. Adv. Sci. *8*, 2003564.
28. Huang, W., Yu, L., Wen, D., Wei, D., Sun, Y., Zhao, H., Ye, Y., Chen, W., Zhu, Y., Wang, L., et al. (2020). A CRISPR-Cas12a-based specific enhancer for more sensitive detection of SARS-CoV-2 infection. EBioMedicine *61*, 103036.
29. Rananaware, S.R., Vesco, E.K., Shoemaker, G.M., Anekar, S.S., Sandoval, L.S.W., Meister, K.S., Macaluso, N.C., Nguyen, L.T., and Jain, P. K. (2023). Programmable RNA detection with CRISPR-Cas12a. Nat. Commun. *14*, 5409.
30. Moon, J., and Liu, C. (2023). Asymmetric CRISPR enabling cascade signal amplification for nucleic acid detection by competitive crRNA. Nat. Commun. *14*, 7504.
31. Wu, Y., Chang, D., Chang, Y., Zhang, Q., Liu, Y., Brennan, J.D., Li, Y., and Liu, M. (2023). Nucleic Acid Enzyme-Activated CRISPR-Cas12a With Circular CRISPR RNA for Biosensing. Small *19*, e2303007.
32. Xiong, Y., Zhang, J., Yang, Z., Mou, Q., Ma, Y., Xiong, Y., and Lu, Y. (2020). Functional DNA regulated CRISPR-Cas12a sensors for point-of-care diagnostics of non-nucleic-acid targets. J. Am. Chem. Soc. *142*, 207–213.
33. Li, T., and Cheng, N. (2023). Sensitive and Portable Signal Readout Strategies Boost Point-of-Care CRISPR/Cas12a Biosensors. ACS Sens. *8*, 3988–4007.
34. Shi, Y., Fu, X., Yin, Y., Peng, F., Yin, X., Ke, G., and Zhang, X. (2021). CRISPR-Cas12a System for Biosensing and Gene Regulation. Chem. Asian J. *16*, 857–867.
35. Xiu, L., Li, H., Hu, Q., Zhang, Y., Chen, S.-B., Wang, C., Zhou, X.-N., Chen, J.-H., and Yin, K. (2023). A versatile microfluidic platform for malaria infection screening and Plasmodium species genotyping. EBioMedicine *98*, 104898.
36. Wang, X., Song, J., Fan, X., Shi, C., Zeng, B., Xiao, Y., Sun, F., and Hu, X. (2023). Universal and highly accurate detection of circulating tumor DNA mutation in non-small cell lung cancer based on CRISPR/Cas12a system. Sens. Actuators B Chem. *383*, 133493.
37. Li, Y., Cai, M., Zhang, W., Liu, Y., Yuan, X., Han, N., Li, J., Jin, S., and Ding, C. (2024). Cas12a-based direct visualization of nanoparticle-stabilized fluorescence signal for multiplex detection of DNA methylation biomarkers. Biosens. Bioelectron. *244*, 115810.
38. Hu, M., Liu, R., Qiu, Z., Cao, F., Tian, T., Lu, Y., Jiang, Y., and Zhou, X. (2023). Light-Start CRISPR-Cas12a Reaction with Caged crRNA Enables Rapid and Sensitive Nucleic Acid Detection. Angew. Chem. Int. Ed. *62*, e202300663.
39. Joung, J., Ladha, A., Saito, M., Kim, N.-G., Woolley, A.E., Segel, M., Barretto, R.P.J., Ranu, A., Macrae, R.K., Faure, G., et al. (2020). Detection of SARS-CoV-2 with SHERLOCK One-Pot Testing. N. Engl. J. Med. *383*, 1492–1494.
40. Wang, B., Wang, R., Wang, D., Wu, J., Li, J., Wang, J., Liu, H., and Wang, Y. (2019). Cas12aVDet: A CRISPR/Cas12a-Based Platform for Rapid and Visual Nucleic Acid Detection. Anal. Chem. *91*, 12156–12161.
41. Wang, R., Qian, C., Pang, Y., Li, M., Yang, Y., Ma, H., Zhao, M., Qian, F., Yu, H., Liu, Z., et al. (2021). opvCRISPR: One-pot visual RT-LAMP-CRISPR platform for SARS-cov-2 detection. Biosens. Bioelectron. *172*, 112766.
42. Arizti-Sanz, J., Freije, C.A., Stanton, A.C., Petros, B.A., Boehm, C.K., Siddiqui, S., Shaw, B.M., Adams, G., Kosoko-Thoroddsen, T.-S.F., Kemball, M.E., et al. (2020). Streamlined inactivation, amplification, and Cas13-based detection of SARS-CoV-2. Nat. Commun. *11*, 5921.
43. Lu, S., Tong, X., Han, Y., Zhang, K., Zhang, Y., Chen, Q., Duan, J., Lei, X., Huang, M., Qiu, Y., et al. (2022). Fast and sensitive detection of SARS-CoV-2 RNA using suboptimal protospacer adjacent motifs for Cas12a. Nat. Biomed. Eng. *6*, 286–297.
44. Yin, K., Ding, X., Li, Z., Zhao, H., Cooper, K., and Liu, C. (2020). Dynamic Aqueous Multiphase Reaction System for One-Pot CRISPR-Cas12a-Based Ultrasensitive and Quantitative Molecular Diagnosis. Anal. Chem. *92*, 8561–8568.

45. Moroz-Omori, E.V., Satyapertiwi, D., Ramel, M.-C., Høgset, H., Sunyovszki, I.K., Liu, Z., Wojciechowski, J.P., Zhang, Y., Grigsby, C.L., Brito, L., et al. (2020). Photoswitchable gRNAs for Spatiotemporally Controlled CRISPR-Cas-Based Genomic Regulation. ACS Cent. Sci. *6*, 695–703.

46. Chen, L., Liu, Y., Guo, W., and Liu, Z. (2022). Light responsive nucleic acid for biomedical application. Exploration *2*, 20210099.

47. Liu, H., Dong, J., Duan, Z., Xia, F., Willner, I., and Huang, F. (2024). Light-activated CRISPR-Cas12a for amplified imaging of microRNA in cell cycle phases at single-cell levels. Sci. Adv. *10*, eadp6166.

48. Wang, X., Dong, K., Kong, D., Zhou, Y., Yin, J., Cai, F., Wang, M., and Ye, H. (2021). A far-red light–inducible CRISPR-Cas12a platform for remote-controlled genome editing and gene activation. Sci. Adv. *7*, eabh2358.

49. Chen, Y., Xu, X., Wang, J., Zhang, Y., Zeng, W., Liu, Y., and Zhang, X. (2022). Photoactivatable CRISPR/Cas12a Strategy for One-Pot DETECTR Molecular Diagnosis. Anal. Chem. *94*, 9724–9731.

50. Li, Q.-N., Wang, D.-X., Chen, D.-Y., Lyu, J.-A., Wang, Y.-X., Wu, S.-L., Jiang, H.-X., and Kong, D.-M. (2024). Photoactivatable CRISPR/Cas12a Sensors for Biomarkers Imaging and Point-of-Care Diagnostics. Anal. Chem. *96*, 2692–2701.

51. Sun, Y.J., Chen, W.D., Liu, J., Li, J.J., Zhang, Y., Cai, W.Q., Liu, L., Tang, X.J., Hou, J., Wang, M., and Cheng, L. (2023). A Conformational Restriction Strategy for the Control of CRISPR/Cas Gene Editing with Photoactivatable Guide RNAs. Angew. Chem. Int. Ed. *62*, e202212413.

52. Zhang, J., Qin, S., Jin, Z., Chen, Q., Xing, L., Qiu, T., Xia, Y., Liang, J., Zhu, H., Wang, L., et al. (2023). The Clinical Significance and Prognostic Role of Whole-Blood Epstein-Barr Virus DNA in Lymphoma-Associated Hemophagocytic Lymphohistiocytosis. J. Clin. Immunol. *43*, 1302–1310.

53. Zeng, M.-C., Jia, Q.-J., Xu, J., Chen, J.-J., Qin, K., and Xu, L.-M. (2023). The whole-blood Epstein-Barr virus DNA can serve as a valuable molecular marker for diagnosis and prognosis prediction of nasopharyngeal carcinoma. Am. J. Cancer Res. *13*, 5431–5442.

54. McMahon, M.A., Prakash, T.P., Cleveland, D.W., Bennett, C.F., and Rahdar, M. (2018). Chemically Modified Cpf1-CRISPR RNAs Mediate Efficient Genome Editing in Mammalian Cells. Mol. Ther. *26*, 1228–1240.

55. Sadelain, M., Papapetrou, E.P., and Bushman, F.D. (2011). Safe harbours for the integration of new DNA in the human genome. Nat. Rev. Cancer *12*, 51–58.

56. Luk, K., Liu, P., Zeng, J., Wang, Y., Maitland, S.A., Idrizi, F., Ponnienselvan, K., Iyer, S., Zhu, L.J., Luban, J., et al. (2022). Optimization of Nuclear Localization Signal Composition Improves CRISPR-Cas12a Editing Rates in Human Primary Cells. GEN Biotechnol. *1*, 271–284.

57. Xu, Z., Wang, Q., Zhong, H., Jiang, Y., Shi, X., Yuan, B., Yu, N., Zhang, S., Yuan, X., Guo, S., and Yang, Y. (2022). Carrier strategies boost the application of CRISPR/Cas system in gene therapy. Exploration *2*, 20210081.

58. Zhu, D., Brookes, D.H., Busia, A., Carneiro, A., Fannjiang, C., Popova, G., Shin, D., Donohue, K.C., Lin, L.F., Miller, Z.M., et al. (2024). Optimal trade-off control in machine learning–based library design, with application to adeno-associated virus (AAV) for gene therapy. Sci. Adv. *10*, eadj3786.

59. Zhou, Y., Zhu, S., Cai, C., Yuan, P., Li, C., Huang, Y., and Wei, W. (2014). High-throughput screening of a CRISPR/Cas9 library for functional genomics in human cells. Nature *509*, 487–491.

60. Piepenburg, O., Williams, C.H., Stemple, D.L., and Armes, N.A. (2006). DNA detection using recombination proteins. PLoS Biol. *4*, e204.

61. Scott, R.S. (2017). Epstein–Barr virus: a master epigenetic manipulator. Curr. Opin. Virol. *26*, 74–80.

62. Shebanova, R., Nikitchina, N., Shebanov, N., Mekler, V., Kuznedelov, K., Ulashchik, E., Vasilev, R., Sharko, O., Shmanai, V., Tarassov, I., et al. (2022). Efficient target cleavage by Type V Cas12a effectors programmed with split CRISPR RNA. Nucleic Acids Res. *50*, 1162–1173.

63. Zhang, W., Zhong, Y., Wang, J., Zou, G., Chen, Q., and Liu, C. (2025). Direct repeat region 3′ end modifications regulate Cas12a activity and expand its applications. Nucleic Acids Res. *53*, gkaf040.

64. Huang, W., Wang, J., Wang, C., Liu, Y., Li, W., Chen, Q., Zhai, J., Xiang, Z., and Liu, C. (2025). Expanding Cas12a Activity Control with an RNA G-Quadruplex at the 5′ end of CRISPR RNA. Adv. Sci. *12*, 2411305.

65. Johnson, D.A., Barclay, R.L., Mergener, K., Weiss, G., König, T., Beck, J., and Potter, N.T. (2014). Plasma Septin9 versus fecal immunochemical testing for colorectal cancer screening: a prospective multicenter study. PLoS One *9*, e98238.

66. Visnes, T., Grube, M., Hanna, B.M.F., Benitez-Buelga, C., Cázares-Körner, A., and Helleday, T. (2018). Targeting BER enzymes in cancer therapy. DNA Repair *71*, 118–126.

67. Fleming, A.M., Ding, Y., and Burrows, C.J. (2017). Oxidative DNA damage is epigenetic by regulating gene transcription via base excision repair. Proc. Natl. Acad. Sci. USA *114*, 2604–2609.

68. Fleming, A.M., and Burrows, C.J. (2020). On the irrelevancy of hydroxyl radical to DNA damage from oxidative stress and implications for epigenetics. Chem. Soc. Rev. *49*, 6524–6528.

69. Harcourt, E.M., Kietrys, A.M., and Kool, E.T. (2017). Chemical and structural effects of base modifications in messenger RNA. Nature *541*, 339–346.

70. Jun, Y.W., and Kool, E.T. (2022). Chemical Tools for the Study of DNA Repair. Acc. Chem. Res. *55*, 3495–3506.

71. Shu, X., Liu, M., Lu, Z., Zhu, C., Meng, H., Huang, S., Zhang, X., and Yi, C. (2018). Genome-wide mapping reveals that deoxyuridine is enriched in the human centromeric DNA. Nat. Chem. Biol. *14*, 680–687.

72. Haldar, T., Jha, J.S., Yang, Z., Nel, C., Housh, K., Cassidy, O.J., and Gates, K.S. (2022). Unexpected complexity in the products arising from NaOH-heat-amine-and glycosylase-induced strand cleavage at an abasic site in DNA. Chem. Res. Toxicol. *35*, 218–232.

73. Kumar, A., Reed, A.J., Zahurancik, W.J., Daskalova, S.M., Hecht, S.M., and Suo, Z. (2022). Interlocking activities of DNA polymerase β in the base excision repair pathway. Proc. Natl. Acad. Sci. USA *119*, e2118940119.

74. Su, M.-T., Liu, I.-H., Wu, C.-W., Chang, S.-M., Tsai, C.-H., Yang, P.-W., Chuang, Y.-C., Lee, C.-P., and Chen, M.-R. (2014). Uracil DNA glycosylase BKRF3 contributes to Epstein-Barr virus DNA replication through physical interactions with proteins in viral DNA replication complex. J. Virol. *88*, 8883–8899.

75. Prichard, M.N., Duke, G.M., and Mocarski, E.S. (1996). Human cytomegalovirus uracil DNA glycosylase is required for the normal temporal regulation of both DNA synthesis and viral replication. J. Virol. *70*, 3018–3025.

76. Dusseau, C., Murray, G.I., Keenan, R.A., O'Kelly, T., Krokan, H.E., and McLeod, H.L. (2001). Analysis of uracil DNA glycosylase in human colorectal cancer. Int. J. Oncol. *18*, 393–399.

77. Lindahl, T., Ljungquist, S., Siegert, W., Nyberg, B., and Sperens, B. (1977). DNA N-glycosidases: properties of uracil-DNA glycosidase from Escherichia coli. J. Biol. Chem. *252*, 3286–3294.

78. Zhang, Q., Li, C.-C., Ma, F., Luo, X., and Zhang, C.-Y. (2022). Catalytic single-molecule Förster resonance energy transfer biosensor for uracil-DNA glycosylase detection and cellular imaging. Biosens. Bioelectron. *213*, 114447.

79. Freudenthal, B.D., Beard, W.A., Cuneo, M.J., Dyrkheeva, N.S., and Wilson, S.H. (2015). Capturing snapshots of APE1 processing DNA damage. Nat. Struct. Mol. Biol. *22*, 924–931.

80. Cheng, Z.-H., Luo, X.-Y., Yu, S.-S., Min, D., Zhang, S.-X., Li, X.-F., Chen, J.-J., Liu, D.-F., and Yu, H.-Q. (2025). Tunable control of Cas12 activity promotes universal and fast one-pot nucleic acid detection. Nat. Commun. *16*, 1166.

STAR★METHODS

KEY RESOURCES TABLE

REAGENT or RESOURCE	SOURCE	IDENTIFIER
Biological samples		
Clinical human blood samples	The Seventh Affiliated Hospital, Sun Yat-sen University	N/A
Chemicals, peptides, and recombinant proteins		
LbCas12a protein	Hylegen Bioscience	Cat#CAS12-010B
EnGen Lba Cas12a (Cpf1)	New England Biolabs	Cat#M0653T
T4 RNA Ligase 1	New England Biolabs	Cat#M0204L
Uracil-DNA Glycosylase (UDG)	New England Biolabs	Cat#M0280L
APE1	New England Biolabs	Cat#M0282S
TranslTX2 Dynamic Delivery System MIR 6000	Mirus bio	Cat#MIR 6000
RNase R	Yeasen Biotechnology	Cat#14606ES72
Dulbecco's Modified Eagle Medium	Gibco	Cat#11995065
Fetal Bovine Serum	Yeasen Biotechnology	Cat#40130ES76
1% penicillin-streptomycin	Bioleaper	Cat#BR3001460
Roswell Park Memorial Institute 1640 medium	Gibco	Cat#11875119
PBS	Biosharp	Cat#BL302A
T7 Endonuclease I	Vazyme Biotech	Cat#EN303-02
VAHTS DNA Clean Beads	Vazyme Biotech	Cat#N411-02
VAHTS RNA Clean Beads	Vazyme Biotech	Cat#N412-02
DNA Dye SuperRed	Biosharp	Cat#BS354B
Opti-MEM I	Thermo Fisher Scientific	Cat#31985070
DNA Dye SuperRed	Biosharp	Cat#BS354B
Stabilized dNTP Mix	GenScript Biotech Corporation	Cat#C01689
dUTP (100 mM)	GenScript Biotech Corporation	Cat#C01717
T7 RNA Polymerase	GenScript Biotech Corporation	Cat#E00066
RIPA Lysis Buffer II	Sangon Biotech	Cat#C510006
ApexHF HS DNA Polymerase FS	Accurate Biotechnology	Cat#AG12201
Hieff Canace Plus High-Fidelity DNA Polymerase	Yeasen Biotechnology	Cat#10153ES76
Critical commercial assays		
RT-RPA 2.0	Keer Life	Cat#901221021
EpiTect Bisulfite Kit	Qiagen	Cat#59104
MolPure Cell/Tissue DNA Kit	Yeasen Biotechnology	Cat#18700ES70
QIAamp DNA Blood Mini Kit	Qiagen	Cat#51183
MolPure PCR Purification Kit	Yeasen Biotechnology	Cat#19106ES50
Enhanced Cell Counting Kit 8 (WST-8/CCK8)	Elabscience Biotechnology	Cat#E-CK-A362
Lateral flow detection strips	Keer Life	Cat#CAS-cmCSA01
Ready to use 2XTaq Master Mix	Generary Biotechnology	Cat#GK8005
DNA Damage Comet Assay Kit	GBCBIO Technologies	Cat#G7668
Human Uracil-DNA Glycosylase (UNG) ELISA Kit	Ziker	Cat#ZK-H2551
Deposited data		
The uncropped raw gel images.xlsx	Figshare	https://doi.org/10.6084/m9.figshare.28887740
Experimental models: Cell lines		
Human cell line HCT116	China Center for Type Culture Collection	Cat#GDC0625
Human cell line SW480	China Center for Type Culture Collection	Cat#GDC0306
Human cell line NCM460	Wuhan Sunncell Biotechnology Co., Ltd	Cat#SNL-519

(*Continued on next page*)

Continued		
REAGENT or RESOURCE	SOURCE	IDENTIFIER
Human cell line Hela	China Center for Type Culture Collection	Cat#GDC0009
Human cell line MCF-7	China Center for Type Culture Collection	Cat#GDC0055
Human cell line HEK 293T	Wuhan Sunncell Biotechnology Co., Ltd	Cat#SNL-015
Oligonucleotides		
Oligonucleotides used in this work	This paper	See Table S1
Software and algorithms		
Prism v9.5.1	GraphPad Software LLC.	https://www.graphpad.com/updates/prism-951-release-notes
Image Lab v5.1	Bio-Rad Laboratories, Inc.	https://www.bio-rad.com/en-cn/product/image-lab-software?ID=KRE6P5E8Z
ImageJ v1.46r	National Institutes of Health (NIH)	https://imagej.net/ij/docs/guide/146.html
Other		
Celemetor Real-Time Fluorescent Quantitative PCR Analysis System	Yeasen Biotechnology Co., Ltd.	Celemetor 96
CFX96 Touch Real-Time PCR Detection System	Bio-Rad Laboratories, Inc.	CFX96 Touch
ChemiDoc MP Imaging System	Bio-Rad Laboratories, Inc.	ChemiDoc MP
Leica DM6B Upright Microscopes	Leica	DM6B

EXPERIMENTAL MODEL AND STUDY PARTICIPANT DETAILS

Cell culture

The HCT116 and SW480 cell lines were obtained from the China Center for Type Culture Collection (CCTCC, Wuhan, China). The NCM460 cell line was purchased from Wuhan Sunncell Biotechnology Co., Ltd (Wuhan, China). All cell lines were authenticated using STR identification and confirmed to be free of contamination. All cell lines were confirmed to be free of Mycoplasma contamination. Culturing of HCT116, SW480, HeLa, MCF-7, and HEK293T cells was performed in Dulbecco's Modified Eagle Medium (DMEM) (Gibco), supplemented with 1% penicillin-streptomycin (Bioleaper) and 10% fetal bovine serum (FBS) (YEASEN), at 37°C, with 5% CO_2. For NCM460 cell culture, Roswell Park Memorial Institute 1640 medium (RPMI-1640) (Gibco) complemented with 10% FBS was used under similar conditions as listed above.

Patient material & consent

Human blood samples were collected and provided by the Seventh Affiliated Hospital, Sun Yat-sen University with protocols approved by the ethics committee at the Seventh Affiliated Hospital, Sun Yat-sen University (KY-2024-009-02 and KY-2023-117-01). All patients in this study signed an informed consent form. 25 blood samples from EBV-infected patients and 20 samples from healthy donors were collected. Using the QIAamp DNA Blood Mini Kit (Qiagen) extracted all clinical human blood samples DNA following the manufacturer's protocol. And the final extracted DNA products were stored at −80°C for further use.

METHOD DETAILS

Preparation and validation of circular RNA

To obtain circular RNA, we mixed a final concentration of 1X Reaction Buffer (50 mM Tris-HCl, pH 7.5, 10 mM $MgCl_2$, 1 mM DTT), 0.5 μM Circular RNA precursor with 5'-phosphoric acid and 3'-hydroxy termini (EBV-Cir-crRNA precursor, Circular DR precursor, Circular-crRNA-dU precursor or Circular-crRNA-THF precursor, Table S1), 1 unit of T4 RNA Ligase 1, 10% (v/v) PEG 8000, 50 μM ATP, and nuclease-free water to a final volume of 20 μL. The reaction mixture was then incubated at 37°C for 1 hour, followed by inactivation of the ligase at 95°C for 2 minutes. Subsequently, 4 units of RNase R were added, and the mixture was further incubated at 37°C for 30 minutes to digest non-circular RNA. The remaining product was heated at 70°C for 10 minutes to inactivate RNase R, followed by PAGE purification for storage at -20°C. The circularization efficiency was validated by performing denaturing 18% PAGE with 7 M urea.

Determining the cleavage time of the PC linker in circular RNA

To determine the time required for the breakage of the PC linker in Circular EBV-Cir-crRNA or Circular DR under UV light exposure (λ= 365 nm, 35 W), we prepared a mixture containing a final concentration of 1X LbCas12a Reaction Buffer (10 mM Tris-HCl, pH 8.5, 10 mM $MgCl_2$, 20 mM KCl, 1 mM DTT, 40 mM glycine, 0.01% (v/v) Tween 20), 1 μM Circular EBV-Cir-crRNA or 2 μM Circular DR and

nuclease-free water to achieve a final volume of 10 μL. Subsequently, the mixture was subjected to UV light exposure for 0 s, 10 s, 30 s, 60 s, 600 s and 1200 s. To evaluate the breakage of the PC linker after UV light exposure, the Circular EBV-Cir-crRNA or Circular DR samples were analyzed using denaturing 18% PAGE with 7 M urea after GelstainRed Nucleic Acid Dye staining.

Photocontrolled AAVS1-Cir-crRNA assisted CRISPR-Cas12a gene editing experiment

HEK 293T cells were seeded at a density of $1x10^5$ cells per well in a 24-well plate. The cells were cultured in Dulbecco's Modified Eagle Medium (DMEM) supplemented with 10% Fetal Bovine Serum and 1% penicillin/streptomycin. The culture conditions were maintained at 37°C in a 5% CO_2 atmosphere for 20 hours until the cells reached approximately 80% confluence. After that, 250 pmol of either AAVS1 crRNA or AAVS1-Cir-crRNA was mixed with 50 μL of Opti-MEM I and gently mixed. Subsequently, 100 pmol of EnGen Lba LbCas12a (Cpf1) was added to the mixture, followed by gentle mixing and incubation at room temperature for 15 minutes. Next, 2 μL of the TransIT-X2 Dynamic Delivery System was added to the transfection mixture, gently mixed again, and incubated at room temperature for an additional 15 minutes. The entire mixture was then added to the cells in the 24-well plate and gently shaken to ensure even distribution. After 6 hours of incubation, the cells were exposed to ultraviolet light (λ = 365 nm, 35 W) for either 3 minutes or left unexposed. The cells were then incubated for an additional 42 hours at 37°C in a 5% CO_2 atmosphere.

T7 endonuclease I (T7EI) assay for evaluating gene editing efficiency

Genomic DNA was extracted from HEK 293T cells 48 hours post-transfection using the MolPure Cell/Tissue DNA Kit. After that, a PCR mixture containing 5 pmol of AAVS1-PCR-F and AAVS1-PCR-R primers, 100 ng of extracted HEK 293T genomic DNA, 1×Canace Plus PCR buffer (with Mg^{2+} and dNTPs), and 1 unit of Hieff Canace Plus High-Fidelity DNA Polymerase was prepared and brought to a final volume of 50 μL with nuclease-free water. The PCR conditions were as follows: initial denaturation at 98°C for 8 minutes, followed by 40 cycles of 98°C for 10 seconds, 60°C for 15 seconds, and 72°C for 30 seconds. A final extension was performed at 72°C for 5 minutes. The PCR products were then purified using the MolPure PCR Purification Kit. Next, A 200 ng aliquot of the purified PCR product was mixed with 1×T7EI Reaction Buffer, and nuclease-free water was added to a final volume of 19 μL. The mixture was subjected to the following annealing protocol: 95°C for 5 minutes, followed by a gradual cooling to 85°C at ∼2°C/second and further cooling to 25°C at ∼0.1°C/second. After annealing, 1 μL of T7EI was added, and the reaction was incubated at 37°C for 20 minutes. The digestion products were immediately analyzed by 2% agarose gel electrophoresis. T7EI specifically cleaves DNA duplexes at mismatched base pairs, generating distinct fragment patterns. In this assay, T7EI targets mutation sites introduced by gene editing, producing fragments of varying lengths compared to the wild-type. The cleavage efficiency, which reflects the percentage of insertions and deletions (Indels), is quantified by analyzing gel electrophoresis images using ImageJ software. This is achieved by calculating the ratio of the intensities of cleaved bands to the total band intensities.

Traditional RPA-Cas12a one-pot nucleic acid detection for EBV

The reaction mixture was prepared in a final volume of 20 μL, containing the following components: 1× LbCas12a Reaction Buffer, 100 nM LbCas12a, 1 nM EBV-specific crRNA, 250 nM F-Q, and 9 μL of RT-RPA 2.0 master mix (each lyophilized pellet dissolved in a total of 25 μL ddH2O). Additionally, 0.1 μM of each primer (EBV-RPA-F and EBV-RPA-R; Table S1) was included, along with varying concentrations of EBV target DNA. Nuclease-free water was used to adjust the total reaction volume to 20 μL. All reagents were thoroughly mixed in qPCR-compatible tubes and incubated at 37°C for 45 minutes in a qPCR instrument. Fluorescence signals were monitored in real-time, and the EBV content in clinical samples was quantified by extrapolating the fluorescence intensity against a pre-established standard curve.

Photocontrolled one-pot method for Clinical EBV sample analysis using EBV-Cir-crRNA or circular DR

For EBV-Cir-crRNA assisted Photocontrolled one-pot Method, we prepared a final concentration of 1X LbCas12a RPA Reaction Buffer (2 mM Tris-HCl, pH 8.5, 28 mM $MgCl_2$, 4 mM KCl, 0.2 mM DTT, 8 mM glycine, 0.002 % (v/v) Tween 20), 100 nM LbCas12a, 1 nM EBV-Cir-crRNA, 250 nM F-Q, 9 μL RT-RPA 2.0 Mix (each lyophilized pellet dissolved in a total of 25 μL ddH_2O), and 0.1 μM of each primer (EBV-RPA-F and EBV-RPA-R, Table S1). We also added 2 μL of either a template made with varying copy numbers of EBV Target described above or a clinical sample to adjust the final volume to 20 μL.

For Circular DR assisted one, the similar conditions are used, we prepared a final concentration of 1X Circular DR-LbCas12a RPA Reaction Buffer (2 mM Tris-HCl, pH 8.5, 28 mM $MgCl_2$, 4 mM KCl, 0.2 mM DTT, 8 mM glycine, 0.002% (v/v) Tween 20, 1 mM ADP, 10 units of T4 Polynucleotide kinase), 100 nM LbCas12a, 100 nM Circular DR, 100 nM EBV Spacer, and 250 nM F-Q, 9 μL RT-RPA 2.0 mix, 0.1 μM of each primer (EBV-RPA-F and EBV-RPA-R). All reagents are mixed in qPCR tubes and placed into the qPCR instrument at 37°C for an RPA amplification of 15 minutes (monitoring fluorescence every 5 minutes). After amplification, the tubes are placed under UV light (λ= 365 nm, 35 W) for 30 seconds before returning to the qPCR instrument for continued fluorescence collection at 37°C for 60 cycles, lasting for 30 minutes. The EBV content in the clinical samples is calculated based on standard curves.

Detection of different UDG enzyme levels in various cell lines using circular-crRNA-dU assisted CRISPR-Cas12 system

Firstly, we prepared a reaction mixture with a final concentration of 0.5 nM Circular-crRNA-dU, 1X LbCas12a-UDG Reaction Buffer (10 mM Tris-HCl, pH 8.5, 10 mM $MgCl_2$, 20 mM KCl, 1 mM DTT, 40 mM glycine, 1 mM spermidine, 0.01% (v/v) Tween 20). Next, we

added 1 μL of UDG diluted with LbCas12a-UDG Reaction Buffer to different final concentrations (500, 250, 125, 62.5, 31.25, 15.63, 7.81, 3.91 U/L), or 1 μL of cell nuclear protein extract diluted with LbCas12a-UDG Reaction Buffer (HCT116, SW480, HeLa, MCF-7 extract diluted 10 times, NCM460, HEK293T extract diluted 2 times). The reaction mixture was brought to a total volume of 10 μL with nuclease-free water, followed by incubation at 37°C for 20 minutes. Finally, we added a final concentration of 100 nM LbCas12a, 250 nM F-Q, and 5 nM EBV Target, and brought the volume to 20 μL with nuclease-free water. The reaction mixture was promptly placed in a qPCR instrument, and fluorescence was collected every 30 seconds at 37°C for a total of 60 cycles, lasting 30 minutes. The UDG enzyme levels in the cell nuclear protein extracts were determined using the standard curve generated with different concentrations of commercial UDG standard (NEB, Cat. No. M0280L).

Detection of nuclear UDG enzyme activity in various cell lines using lateral flow assay strips

A reaction mixture was prepared containing 0.5 nM Circular-crRNA-dU in LbCas12a-UDG Reaction Buffer (10 mM Tris-HCl, pH 8.5, 10 mM MgCl2, 20 mM KCl, 1 mM DTT, 40 mM glycine, 1 mM spermidine, 0.01% (v/v) Tween 20). For the standard curve, 3 μL of UDG at various concentrations (4000, 800, 400, 200, 100, 50, 25, 12.5, 0 U/L) was added. For sample analysis, 3 μL of nuclear protein extract from HCT116, SW480, NCM460, MCF-7, HeLa, and HEK 293T cells was added. The total volume was adjusted to 60 μL with nuclease-free water, and the mixture was incubated at 37°C for 20 minutes. Subsequently, 100 nM LbCas12a, 250 nM F-Q, and 5 nM EBV Target were added to the reaction, and the volume was adjusted to 20 μL with nuclease-free water. After incubation at 37°C for 20 minutes, a lateral flow assay strip was inserted into the reaction tube and allowed to react for 8 minutes. The results were observed and photographed, followed by analysis using ImageJ software.

QUANTIFICATION AND STATISTICAL ANALYSIS

Statistical analysis and data interpretation

All experimental data were analyzed using GraphPad Prism software (version 9.5.1). Results are presented as the mean ± standard deviation (SD) from three independent replicates, unless otherwise specified. Nonlinear regression was performed using the Sigmoidal, 4PL (four-parameter logistic) model with concentration (X) as the independent variable, while linear regression was conducted using the Simple Linear Regression tool. Statistical comparisons were performed using unpaired two-tailed t-tests, with $p > 0.05$ considered not significant and $p < 0.05$ deemed statistically significant. For real-time fluorescence curve analysis, the endpoint fluorescence intensity was used for bar graph representations unless otherwise noted. The time points for establishing the linear relationship between target concentration and fluorescence intensity were selected based on the following criteria: the fluorescence curve must remain in the linear phase without reaching the plateau, the fluorescence intensity at the chosen time point should exhibit significant differences across the target concentration gradient, and the intensity must demonstrate a strong linear correlation with target concentration, as evidenced by an R^2 value greater than 0.9. This approach ensures robust and reproducible quantification of target analytes.

Cell Reports Methods, Volume *5*

Supplemental information

Enhancement of CRISPR-Cas12a system through universal circular RNA design

Jiaqi Wang, Wei Zhang, Wentao Li, Qinyuan Xie, Ziyu Zang, and Chaoxing Liu

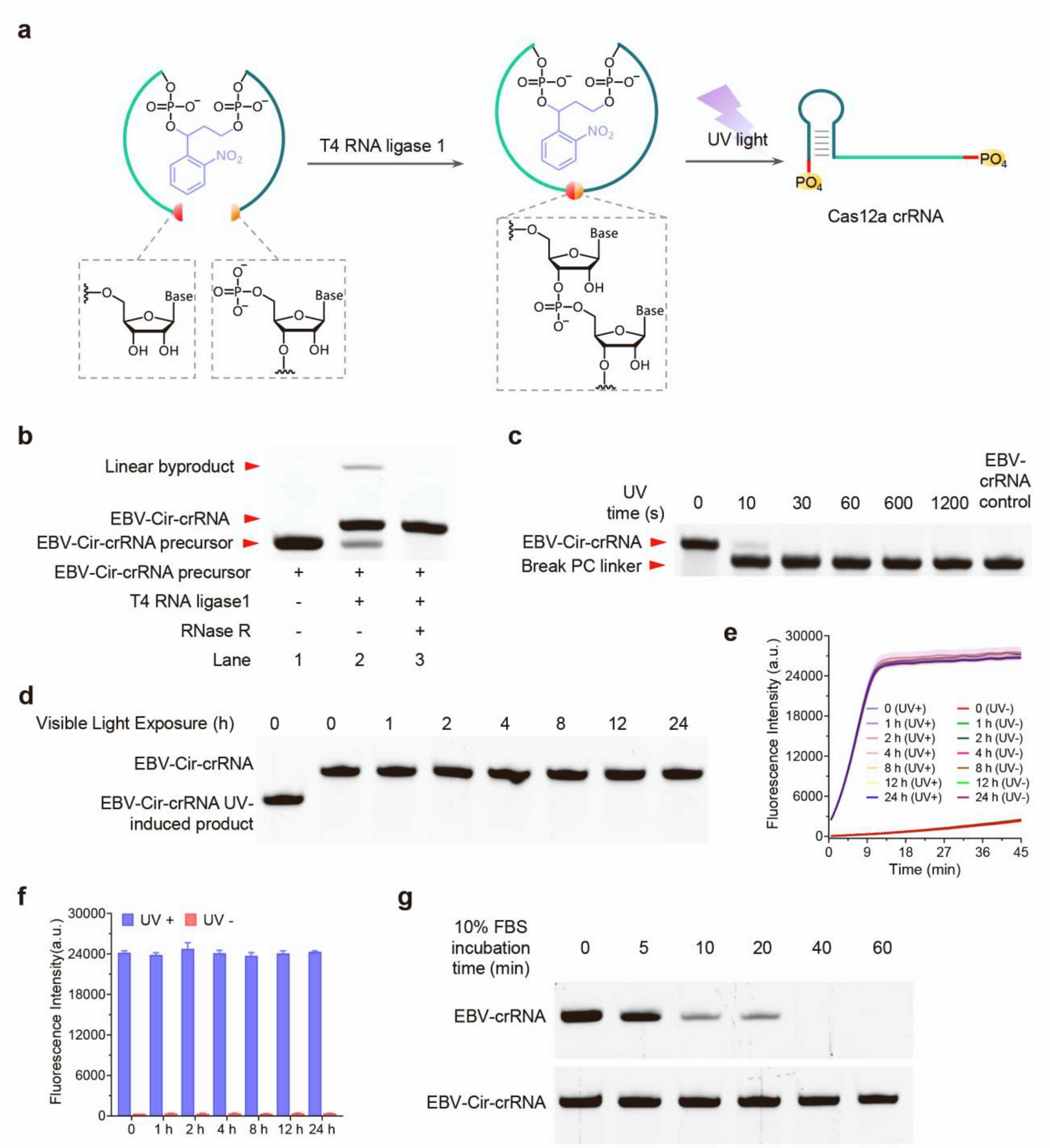

Figure S1: Preparation and verification of circular RNA, related to Figures 1, 2. (a) A schematic illustration of the creation of circular crRNA and the subsequent disruption at the PC linker of the circular crRNA under UV light, reverting to its classic configuration. (b) PAGE analysis of EBV-Cir-crRNA precursor producing a circular product under the action of T4 RNA Ligase1. Subsequently, RNase R is used to remove all linear molecules, including intermolecular ligated products and unreacted starting materials. (c) PAGE analysis of the condition of the PC linker of EBV-Cir-crRNA after UV radiation

(365 nm, 35w) for 0-1200s. From the results, it can be seen that upon 30s of UV exposure, EBV-Cir-crRNA completely breaks and reverts to the non-circular state and no significant changes occur even up to 20 minutes. (d) Structural stability assessment of EBV-Cir-crRNA under normal laboratory lighting. 100 ng of EBV-Cir-crRNA was incubated in transparent 0.2 mL microcentrifuge tubes on ice for 0, 1, 2, 4, 8, 12, and 24 hours, separately. For comparison, 100 ng of UV-induced EBV-Cir-crRNA cleavage product was used as a positive control for PC linker disruption. All samples were analyzed by 12% denaturing PAGE (150 V, 30 min) to evaluate PC linker integrity. The results demonstrated the robust structural stability of circular RNA under standard laboratory conditions. (e) Real-time fluorescence monitoring of LbCas12a *trans*-cleavage activity assays comparing EBV-Cir-crRNA exposed to standard laboratory lighting versus unexposed controls over a 24-hour period. No significant variation in LbCas12a activation was observed, confirming the photostability of the circular RNA design. (f) Quantitative analysis of fluorescence intensity at the 45-minute time point from panel (e), presented as a bar graph for direct comparison. (g) Enhanced environmental stability of EBV-Cir-crRNA compared to linear EBV crRNA. 100 ng of each RNA was incubated with 2 μL of Dulbecco's Modified Eagle Medium (DMEM) supplemented with 10% fetal bovine serum (FBS) at 37 °C for 0, 5, 10, 20, 40, and 60 minutes, separately. Analysis by 12% denaturing PAGE (150 V, 30 min) revealed that linear crRNA exhibited significant degradation within 10 minutes and was completely degraded by 40 minutes, whereas EBV-Cir-crRNA maintained its structural integrity throughout the 60-minute incubation period. Error bars represent the mean ± standard deviation (S.D.) of triplicate measurements, ensuring statistical reliability.

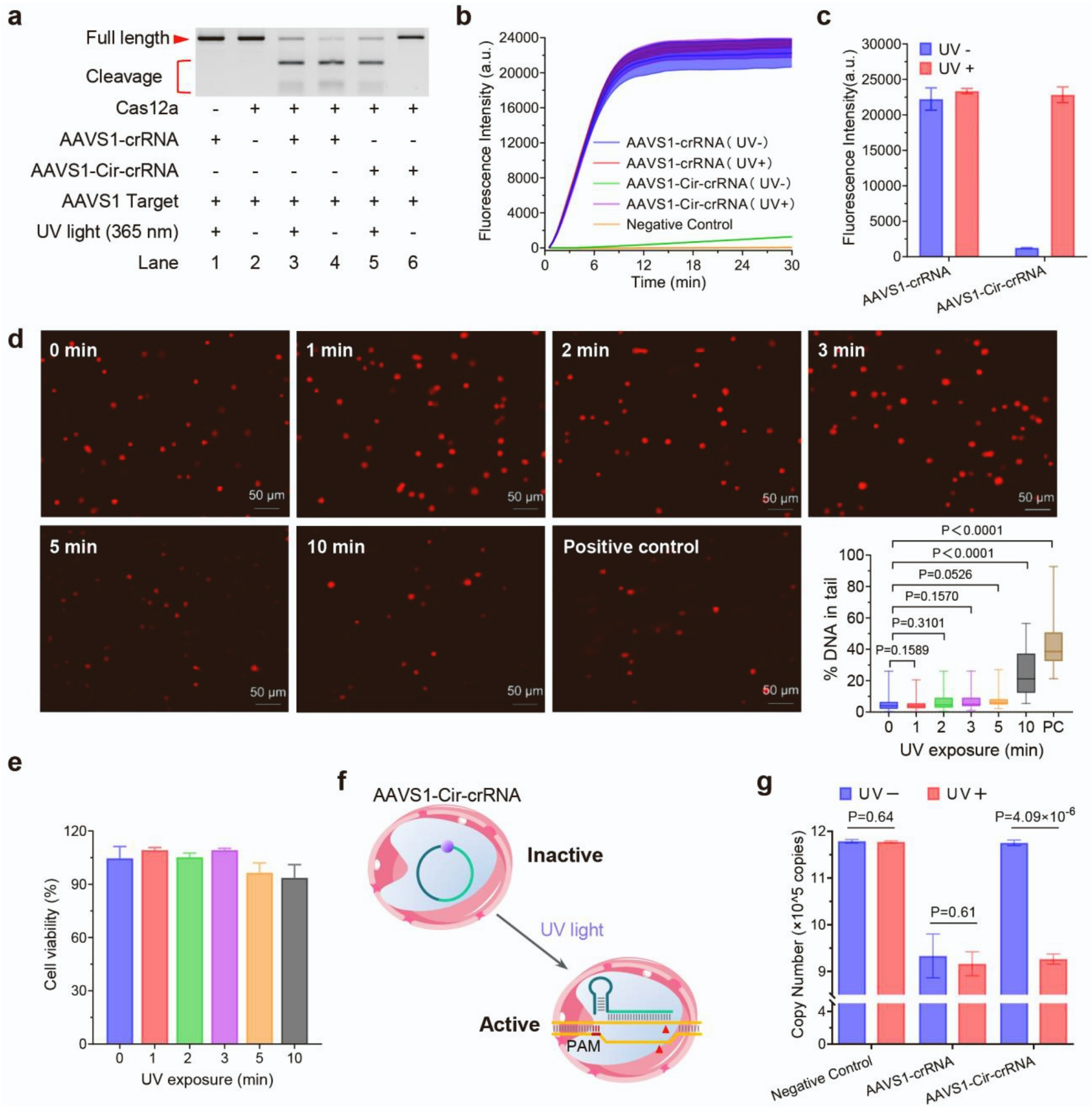

Figure S2: Functional characterization of circular AAVS1-Cir-crRNA with a PC linker in CRISPR-Cas12a activity and UV-induced effects, related to Figure 2. (a) Analysis on a 2% agarose gel confirms that circular AAVS1-Cir-crRNA with a PC linker effectively inhibits Cas12a *cis*-cleavage activity towards the AAVS1 Target. Notably, half a minute under UV light can restore this activity. (b) Investigation of circularized crRNA targeting the AAVS1 locus with a PC linker. Fluorescence release from the corresponding DNA target was measured in vitro, induced by LbCas12a *trans*-cleavage activity under two conditions: with and without 30-second UV irradiation. The results demonstrate that circularized crRNA effectively inhibits LbCas12a activity, while UV irradiation for 30 seconds restores

its function. (c) Fluorescence intensity data of (b) at the 30-minute time point are compared under two conditions: with and without UV light exposure. (d) Genome-wide assessment of UV-induced DNA damage. HEK293T cells were exposed to UV light (λ = 365 nm) for 1, 2, 3, 5, and 10 minutes with the lamp 15 cm above culture dishes on ice. Untreated cells (negative control) and cells treated with 50 μM H_2O_2 for 1 hour (positive control) were used. Post-exposure, cells were trypsinized, washed twice with PBS, and resuspended at 1×10^6 cells/mL. The comet assay was performed using a DNA Damage Comet Assay Kit (GBCBIO Technologies, Cat. No. G7668) following the manufacturer's protocol. Images were acquired via fluorescence microscopy and analyzed using the OpenComet plugin in ImageJ. (e) UV irradiation effects on cell viability. HEK293T cells were exposed to UV light (λ = 365 nm) for 0, 1, 2, 3, 5, and 10 minutes. Viability remained stable for 0-3 minutes but showed a slight decline after 5 minutes. (f) Schematic showing the influence of circular AAVS1-Cir-crRNA with a PC linker assisted CRISPR-Cas12a system's gene editing at the cellular level with or without UV light exposure. (g) A bar chart depicts the unedited AAV1 gene copy number in AAVS1 crRNA, AAVS1-Cir-crRNA assisted CRISPR-Cas12a system's gene editing at the cellular level with and without UV light exposure. All experiments were performed in triplicate; error bars represent mean ± S.D., $P > 0.05$ indicates no statistically significant difference.

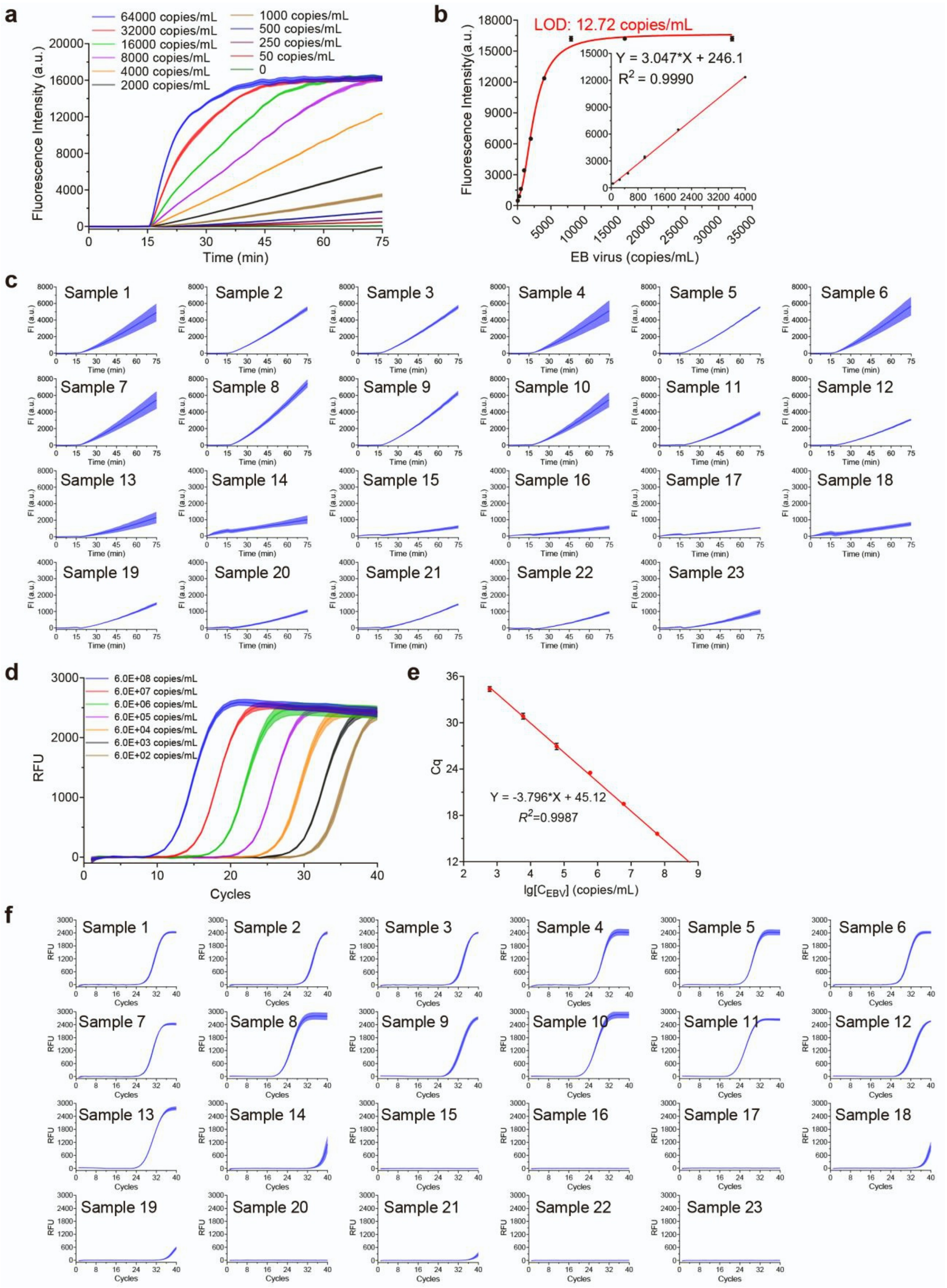
a
64000 copies/mL
32000 copies/mL
16000 copies/mL
8000 copies/mL
4000 copies/mL
2000 copies/mL
1000 copies/mL
500 copies/mL
250 copies/mL
50 copies/mL
0
Fluorescence Intensity (a.u.)
Time (min)
b
LOD: 12.72 copies/mL
Y = 3.047*X + 246.1
R2 = 0.9990
Fluorescence Intensity(a.u.)
EB virus (copies/mL)
c
Sample 1
Sample 2
Sample 3
Sample 4
Sample 5
Sample 6
Sample 7
Sample 8
Sample 9
Sample 10
Sample 11
Sample 12
Sample 13
Sample 14
Sample 15
Sample 16
Sample 17
Sample 18
Sample 19
Sample 20
Sample 21
Sample 22
Sample 23
FI (a.u.)
Time (min)
d
6.0E+08 copies/mL
6.0E+07 copies/mL
6.0E+06 copies/mL
6.0E+05 copies/mL
6.0E+04 copies/mL
6.0E+03 copies/mL
6.0E+02 copies/mL
RFU
Cycles
e
Y = -3.796*X + 45.12
R2=0.9987
Cq
lg[CEBV] (copies/mL)
f
Sample 1
Sample 2
Sample 3
Sample 4
Sample 5
Sample 6
Sample 7
Sample 8
Sample 9
Sample 10
Sample 11
Sample 12
Sample 13
Sample 14
Sample 15
Sample 16
Sample 17
Sample 18
Sample 19
Sample 20
Sample 21
Sample 22
Sample 23
RFU
Cycles

Figure S3: Development and verification of the RPA-CRISPR Cas12a one-pot method assisted by circular crRNA with a PC linker, related to Figure 2. (a) Fluorescent graphs of the detection of different concentrations of EB virus using this method. Error bars represent mean ± S.D. (n = 3). (b) Linear fit graphs of EB virus detection. (c) Raw fluorescence data recording for detection of clinical blood samples by current developed method, infected or not with the EB virus. First, nucleic acids were extracted from 200 μL of whole blood samples, eluted to 20 μL nuclease-free ddH_2O, then 2 μL of each sample was added to the current one-pot detection system, and the volume was filled up to 20 μL with nuclease-free ddH_2O. After 15 minutes of RPA at 37 °C, the samples were exposed to UV light for 30s, and fluorescence variations are then monitored and recorded at 37 °C. Detection of EB virus using the traditional qPCR method (d) Fluorescent graphs for detecting different concentrations of the EB virus using the traditional qPCR method, and (e) the corresponding linear fit graphs. (f) Raw fluorescence data recordings for the detection of clinical blood samples using the traditional qPCR method, in order to make a comparison with Figure S3c.

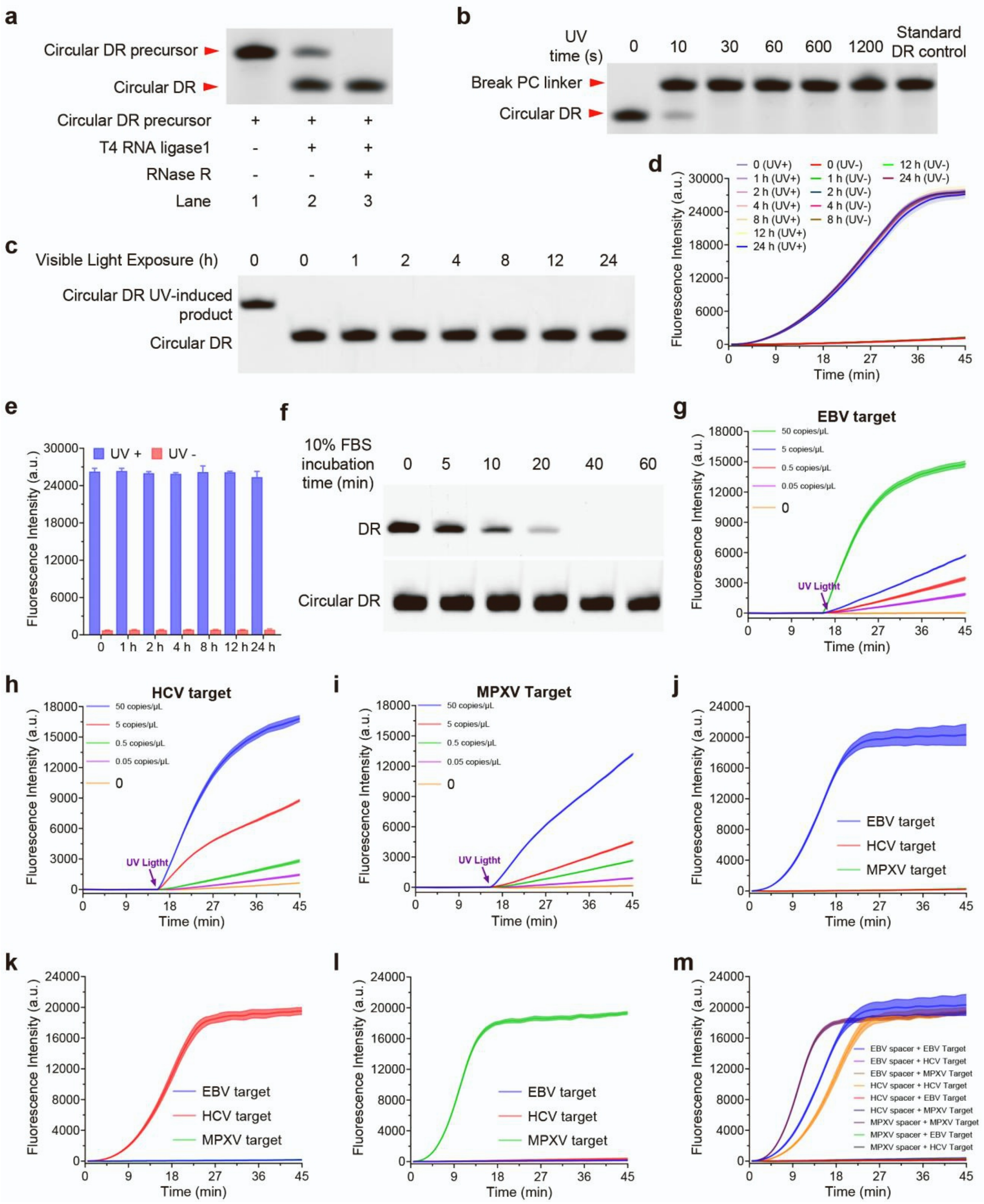

Figure S4: Comprehensive characterization and functional validation of circular DR with PC linker stability and development of RPA-CRISPR/Cas12a one-pot method for different target detection, related to Figure 3. (a) PAGE analysis of Circular DR with a PC linker precursor and its

circularization product. Circularization of the precursor was achieved using T4 RNA Ligase 1, followed by RNase R treatment to eliminate linear molecules, including intermolecular ligation products and unreacted starting materials. (b) UV irradiation effects on the PC linker of Circular DR. Circular DR with a PC linker was exposed to UV light (365 nm, 35 W) for 0-1200 s, and its integrity was assessed by 12% denaturing PAGE (150 V, 30 min). (c) Structural stability of Circular DR with a PC linker under laboratory lighting. Circular DR with a PC linker (100 ng) was incubated in transparent 0.2 mL microcentrifuge tubes on ice for 0, 1, 2, 4, 8, 12, and 24 hours. UV-induced cleavage products served as positive controls. PAGE analysis confirmed the robust stability of the PC linker under standard laboratory conditions. (d) Real-time fluorescence monitoring of LbCas12a trans-cleavage activity. Circular DR with a PC linker + EBV Spacer was exposed to standard laboratory lighting versus unexposed controls over 24 hours. No significant variation in LbCas12a activation was observed, demonstrating the photostability of the circular RNA design. (e) Quantitative fluorescence intensity analysis. Fluorescence intensity at the 45-minute time point from panel (d) was quantified and presented as a bar graph for direct comparison. (f) Environmental stability of Circular DR with a PC linker. Circular DR with a PC linker and canonical DR (100 ng each) were incubated with DMEM + 10% FBS at 37 °C for 0, 5, 10, 20, 40, and 60 minutes. PAGE analysis revealed that canonical DR degraded significantly within 10 minutes and completely by 40 minutes, while Circular DR with a PC linker maintained full integrity throughout the 60-minute incubation. (g) Development and verification of the RPA-CRISPR Cas12a one-pot method assisted by circular DR, to confirm its versatility for detecting various targets when combined with different Spacer regions. Fluorescent graphs of the detection of different concentrations of the EB virus. (h) Fluorescent graphs of the detection of different concentrations of the Hepatitis C virus (HCV). (i) Fluorescent graphs of the detection of different concentrations of Monkeypox virus (MPXV). Cross-verification of the detection of different viruses (EBV, HCV, MPXV) using the universal circular DR combined with different Spacers. (j) Fluorescent graphs of the detection of different viruses (EBV, HCV, MPXV) using the universal circular DR combined with EBV Spacer. Only EBV Target can be detected. (k) Fluorescent graphs of the detection of different viruses using the current method combined with MPXV Spacer. Only MPXV Target can be detected. (l) Fluorescent graphs of the detection of different viruses using the current method combined with HCV Spacer. Only HCV Target can be detected. (m) Raw fluorescence data recording for cross-verification of the detection of different viruses in Figure 3f. All experiments were performed in triplicate; error bars represent mean ± S.D. of triplicate measurements, ensuring statistical reliability.

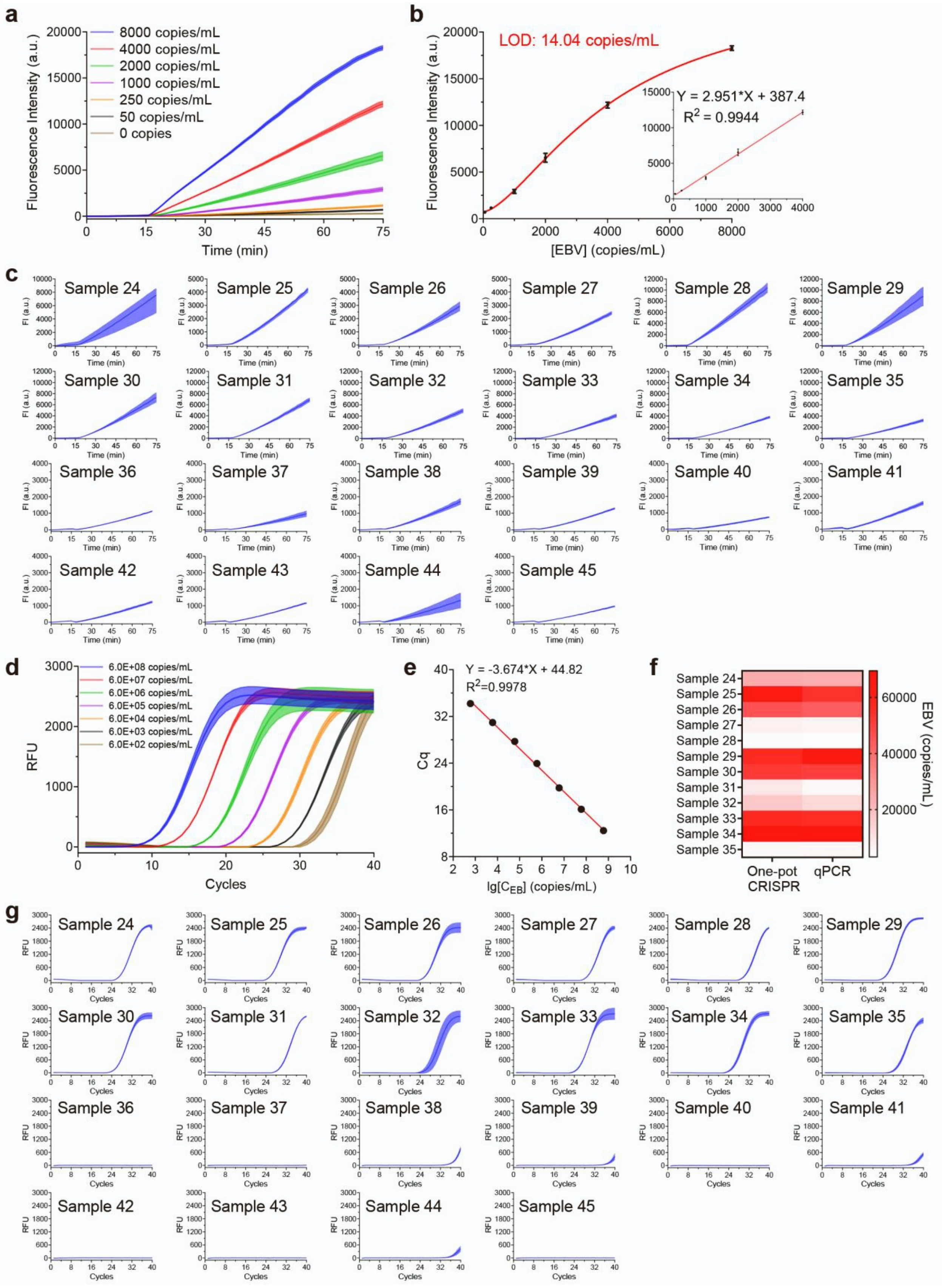
a
8000 copies/mL
4000 copies/mL
2000 copies/mL
1000 copies/mL
250 copies/mL
50 copies/mL
0 copies
Fluorescence Intensity (a.u.)
Time (min)
b
LOD: 14.04 copies/mL
Y = 2.951*X + 387.4
R2 = 0.9944
Fluorescence Intensity (a.u.)
[EBV] (copies/mL)
c
Sample 24
Sample 25
Sample 26
Sample 27
Sample 28
Sample 29
Sample 30
Sample 31
Sample 32
Sample 33
Sample 34
Sample 35
Sample 36
Sample 37
Sample 38
Sample 39
Sample 40
Sample 41
Sample 42
Sample 43
Sample 44
Sample 45
FI (a.u.)
Time (min)
d
6.0E+08 copies/mL
6.0E+07 copies/mL
6.0E+06 copies/mL
6.0E+05 copies/mL
6.0E+04 copies/mL
6.0E+03 copies/mL
6.0E+02 copies/mL
RFU
Cycles
e
Y = -3.674*X + 44.82
R2=0.9978
Cq
lg[CEB] (copies/mL)
f
One-pot CRISPR
qPCR
EBV (copies/mL)
g
RFU
Cycles

Figure S5: Development and verification of the universal circular DR assisted RPA-CRISPR Cas12a one-pot method, related to Figure 3. (a) Fluorescent graphs of the detection of different concentrations of EB virus using this method. Error bars represent mean ± S.D. (n = 3). (b) Linear fit graphs of EB virus detection. (c) Raw fluorescence data recording for detection of clinical blood samples by current developed method, infected or not with the EB virus. Detection of EB virus using the traditional qPCR method, in order to make a comparison with Figures S5a-c. (d) Fluorescent graphs for detecting different concentrations of the EB virus using the traditional qPCR method, and (e) the corresponding linear fit graphs. (f) Comparisons of the detections of clinical blood samples using the universal DR-assisted one-pot RPA-CRISPR Cas12a method and the traditional qPCR method. (g) Raw fluorescence data recordings for the detection of clinical blood samples using the traditional qPCR method, in order to make a comparison with Figure S5c.

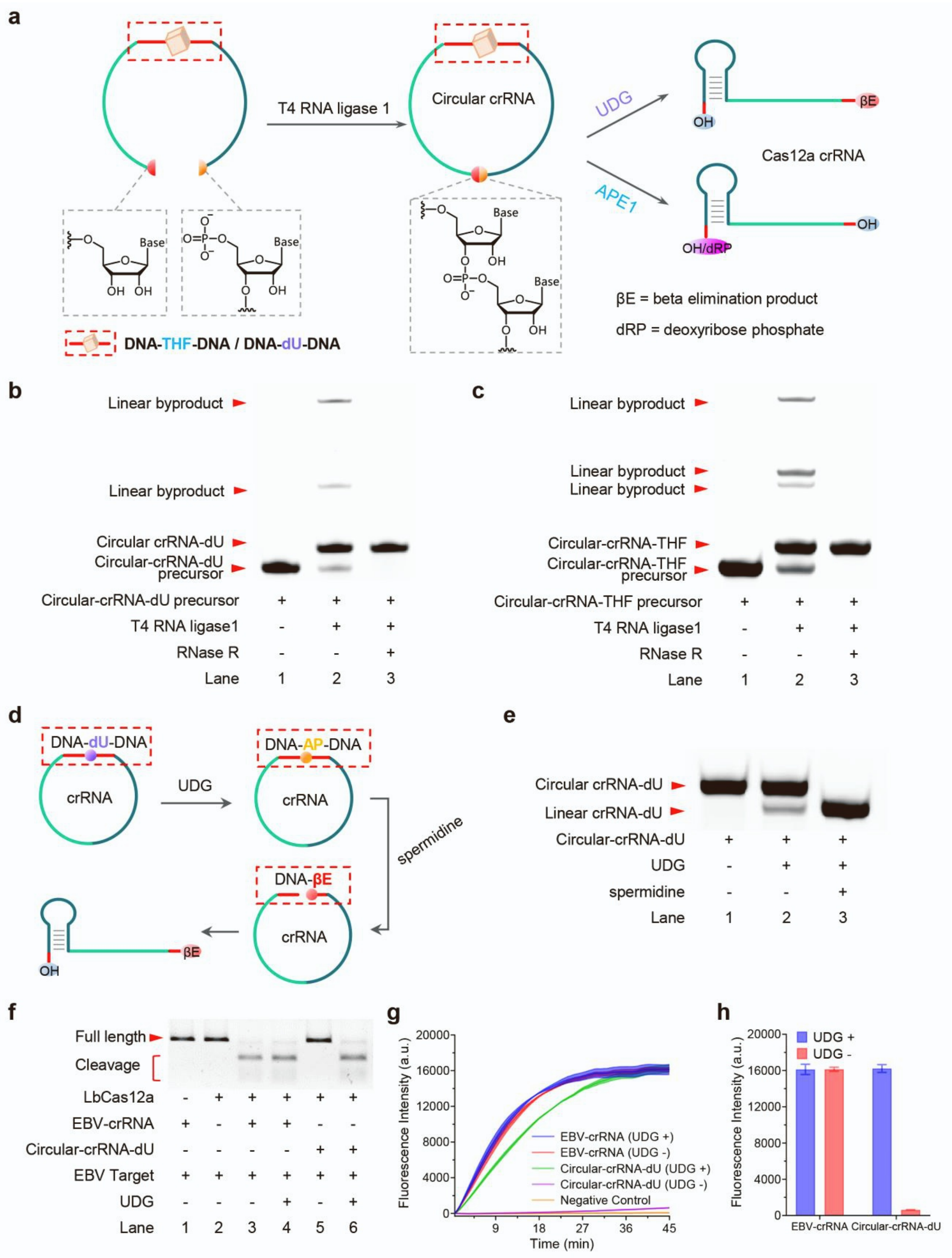
a
T4 RNA ligase 1
Circular crRNA
UDG
APE1
OH
βE
Cas12a crRNA
OH/dRP
OH
Base
DNA-THF-DNA / DNA-dU-DNA
βE = beta elimination product
dRP = deoxyribose phosphate
b
Linear byproduct
Linear byproduct
Circular crRNA-dU
Circular-crRNA-dU precursor
Circular-crRNA-dU precursor
T4 RNA ligase1
RNase R
Lane 1 2 3
c
Linear byproduct
Linear byproduct
Linear byproduct
Circular-crRNA-THF
Circular-crRNA-THF precursor
Circular-crRNA-THF precursor
T4 RNA ligase1
RNase R
Lane 1 2 3
d
DNA-dU-DNA
crRNA
UDG
DNA-AP-DNA
spermidine
DNA-βE
OH
βE
e
Circular crRNA-dU
Linear crRNA-dU
Circular-crRNA-dU
UDG
spermidine
Lane 1 2 3
f
Full length
Cleavage
LbCas12a
EBV-crRNA
Circular-crRNA-dU
EBV Target
UDG
Lane 1 2 3 4 5 6
g
Fluorescence Intensity (a.u.)
Time (min)
EBV-crRNA (UDG +)
EBV-crRNA (UDG -)
Circular-crRNA-dU (UDG +)
Circular-crRNA-dU (UDG -)
Negative Control
h
UDG +
UDG -
Fluorescence Intensity (a.u.)
EBV-crRNA
Circular-crRNA-dU

Figure S6: Preparation and verification of circular crRNA with dU or THF modifications, related to Figure 4. (a) A schematic illustration of the preparation of circular crRNA with dU or THF modifications, and the subsequent disruption at the modified base of the circular RNA under UDG or APE1 conditions, reverting to its classic configuration. PAGE analysis showing the successful circulation of circular crRNA with dU (b) or THF modifications (c). Verification of the working principle of circular crRNA with dU modification. (d) Schematic of the working principle. dU is going to form AP Sites acting under UDG. Then, the addition reaction occurs between the AP sites with spermidine in the buffer solution, forming a beta elimination product (βE), opening the circular structure, and restoring the circular crRNA to its classic structure. (e) PAGE analysis to verify the principle in (d). Breakdown products are formed under the action of UDG and spermidine, consistent with the research report by Kent S Gate, et al (*Chem. Res. Toxicol.* 2022, 35, 218-232). Inhibition and restoration of *cis*- and *trans*-cleavage activities of the CRISPR-Cas12a system mediated by dU-modified circular crRNA and UDG enzyme. (f) Comparison of *cis*-cleavage activity in the CRISPR-Cas12a system facilitated by dU-modified circular crRNA versus conventional EBV crRNA, alongside the recovery of *cis*-cleavage activity following UDG enzyme treatment of the dU-modified circular crRNA. Reactions were incubated for 45 minutes. (g) Evaluation of *trans*-cleavage activity in the CRISPR-Cas12a system using dU-modified circular crRNA versus EBV crRNA, and the restoration of *trans*-cleavage activity upon UDG enzyme treatment of the dU-modified circular crRNA. (h) Bar graph representation of the fluorescence intensity at 45 minutes for the conditions described in panel (g), providing a quantitative comparison of fluorescence intensity generated by *trans*-cleavage activity.

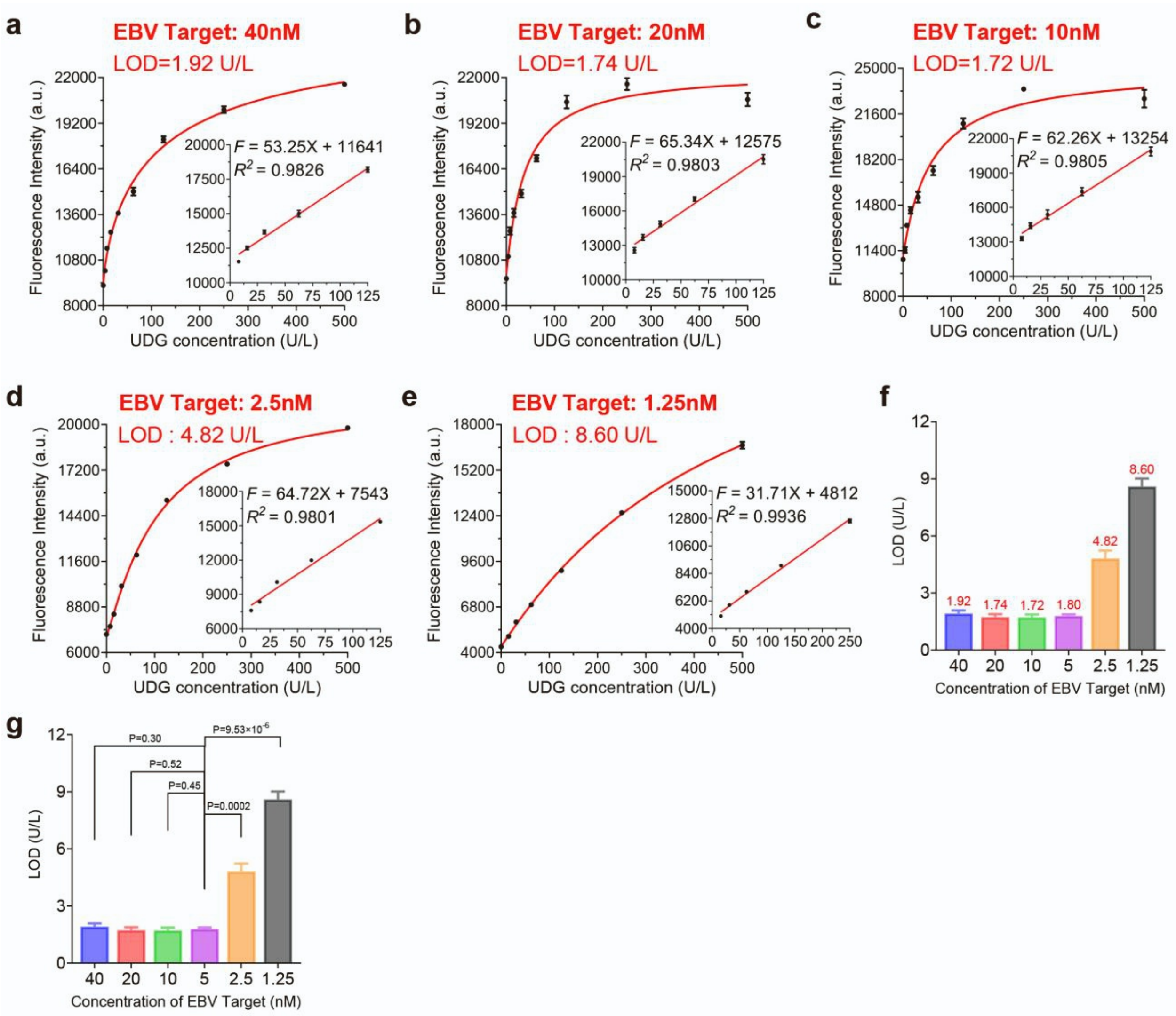

Figure S7: Relationship between initial EBV target concentration and UDG detection limit in the circular crRNA-based detection system, related to Figure 4. (a) UDG detection limit evaluated at 40 nM EBV target concentration. (b) UDG detection limit evaluated at 20 nM EBV target concentration. (c) UDG detection limit evaluated at 10 nM EBV target concentration. (d) UDG detection limit evaluated at 2.5 nM EBV target concentration. (e) UDG detection limit evaluated at 1.25 nM EBV target concentration. (f) Comparative bar graph illustrating the detection limits across EBV target concentrations of 40 nM, 20 nM, 10 nM, 2.5 nM, 1.25 nM, and 5 nM (as shown in Figure 3b of the main text). (g) Corresponding p-values from statistical analysis, where $p > 0.05$ indicates no significant differences. All experiments were performed in triplicate ($n = 3$), with error bars representing mean ± S.D.

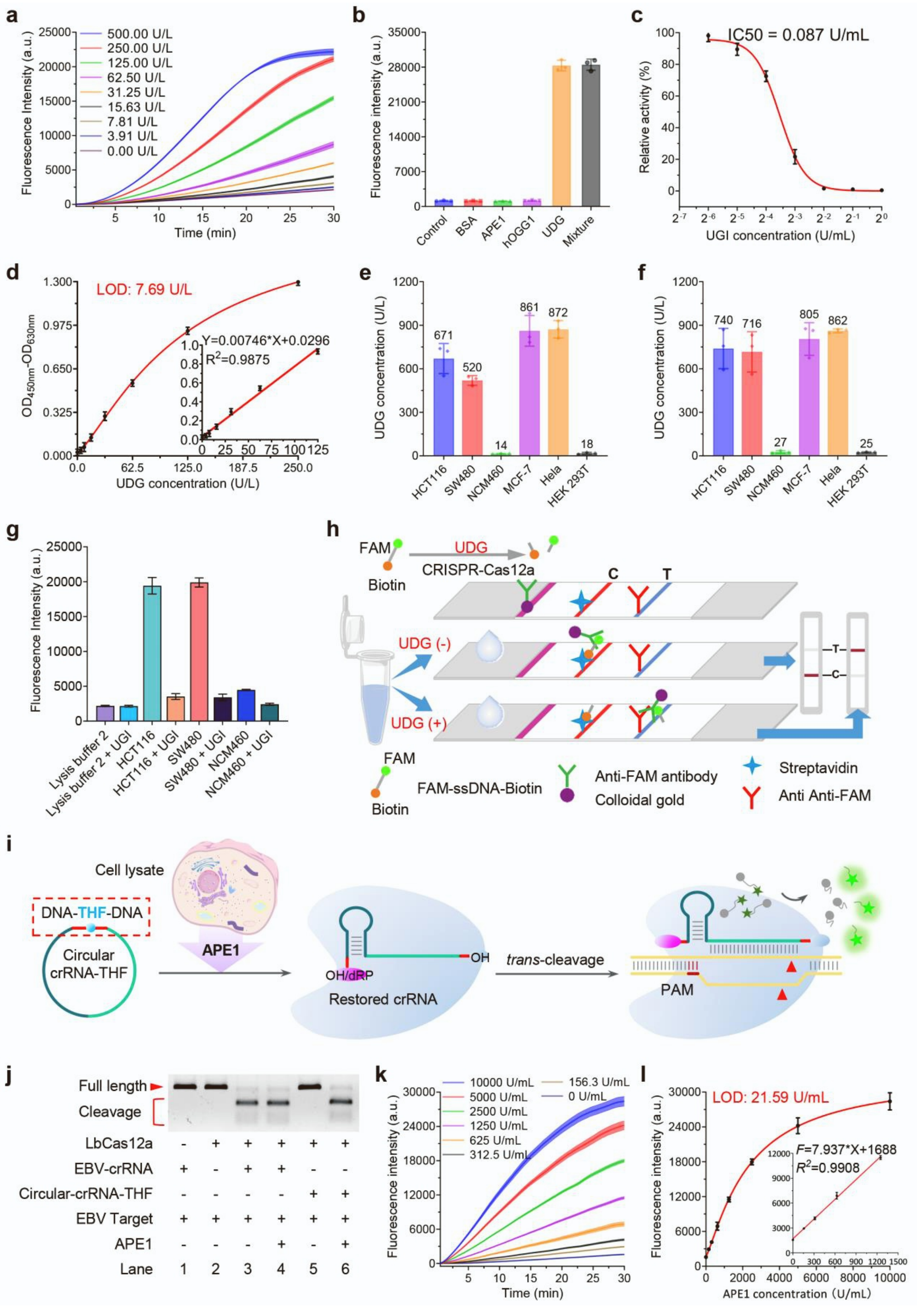
a
Fluorescence Intensity (a.u.)
Time (min)
500.00 U/L
250.00 U/L
125.00 U/L
62.50 U/L
31.25 U/L
15.63 U/L
7.81 U/L
3.91 U/L
0.00 U/L
b
Fluorescence intensity (a.u.)
Control
BSA
APE1
hOGG1
UDG
Mixture
c
IC50 = 0.087 U/mL
Relative activity (%)
UGI concentration (U/mL)
d
LOD: 7.69 U/L
Y=0.00746*X+0.0296
R2=0.9875
UDG concentration (U/L)
e
UDG concentration (U/L)
671
520
14
861
872
18
HCT116
SW480
NCM460
MCF-7
Hela
HEK 293T
f
UDG concentration (U/L)
740
716
27
805
862
25
g
Fluorescence Intensity (a.u.)
Lysis buffer 2
Lysis buffer 2 + UGI
HCT116
HCT116 + UGI
SW480
SW480 + UGI
NCM460
NCM460 + UGI
h
FAM
UDG
CRISPR-Cas12a
Biotin
C
T
UDG (-)
UDG (+)
FAM-ssDNA-Biotin
Anti-FAM antibody
Colloidal gold
Streptavidin
Anti Anti-FAM
i
Cell lysate
DNA-THF-DNA
Circular crRNA-THF
APE1
OH
OH/dRP
Restored crRNA
trans-cleavage
PAM
j
Full length
Cleavage
LbCas12a
EBV-crRNA
Circular-crRNA-THF
EBV Target
APE1
Lane
k
10000 U/mL
5000 U/mL
2500 U/mL
1250 U/mL
625 U/mL
312.5 U/mL
156.3 U/mL
0 U/mL
Fluorescence intensity (a.u.)
Time (min)
l
LOD: 21.59 U/mL
F=7.937*X+1688
R2=0.9908
Fluorescence intensity (a.u.)
APE1 concentration (U/mL)

Figure S8: Verification of the selectivity of the detection of UDG by the CRISPR-Cas12a system, assisted by circular RNA with dU modification, related to Figure 4. (a) Raw fluorescence data graphs for varying concentrations detected by current detection system. (b) The fluorescence graphs of Bovine Serum Albumin (BSA), APE1, Recombinant Human OGG1 (hOGG1), and Uracil-DNA Glycosylase (UDG), as well as their mixtures tested in the current detection system, validating the specificity of the system for UDG detection. (c) Suppression of the Circular-crRNA-dU assisted CRISPR Cas12 detection system by UGI, UDG's inhibitor, demonstrating Circular-crRNA-dU assisted CRISPR Cas12 detection system regulation through various UGI concentrations. The calculated IC50 for UGI is 0.087 U/mL. (d) The linear fit graphs corresponding to the commercial Human Uracil-DNA Glycosylase (UNG) ELISA Kit (Ziker, cat. no. ZK-H2551) accurately detect the known UDG concentration. (e) A bar chart displaying the levels of UDG in various cell lines as detected by the currently developed methods. (f) A bar chart illustrating the levels of UDG in different cell lines as detected by the commercial Human Uracil-DNA Glycosylase (UNG) ELISA Kit. (g) Assessment of UDG detection system compatibility with various cell lysates. Fluorescence intensity profiles of HCT116, SW480, and NCM460 cell lysates, including samples supplemented with UGI, were measured using the current UDG detection system at the 30-minute time point. These results demonstrate the system's applicability beyond nuclear protein extracts, validating its robustness for broader biological sample analysis. (h) A schematic depiction of the paper strip-based visual detection device designed utilizing the current developed CRISPR-Cas12a system, supported by circular RNA with dU modification. CRISPR-Cas12a system assisted by crRNA with THF modification. (i) Schematic of the working principle of the CRISPR-Cas12a system aided by circular crRNA with THF modification. (j) PAGE analysis validating the inhibition of the *cis*-cleavage activity of the CRISPR-Cas12a system by circular crRNA with THF modification, and its reactivation by APE1. Varying concentrations of APE1 detected by the system show the suppression of *trans*-cleavage activity by circular crRNA with THF modification (0.00 U/mL), and its subsequent reactivation by APE1 with fluorescence intensity increasing with concentration. (k-l) The correlation between the fluorescent values (k) and the linear fit (l) of different concentrations of APE1 detected by the CRISPR-Cas12a system, assisted by circular crRNA with THF modification. All experiments were performed in triplicate, and error bars represent mean ± S.D. (n = 3).

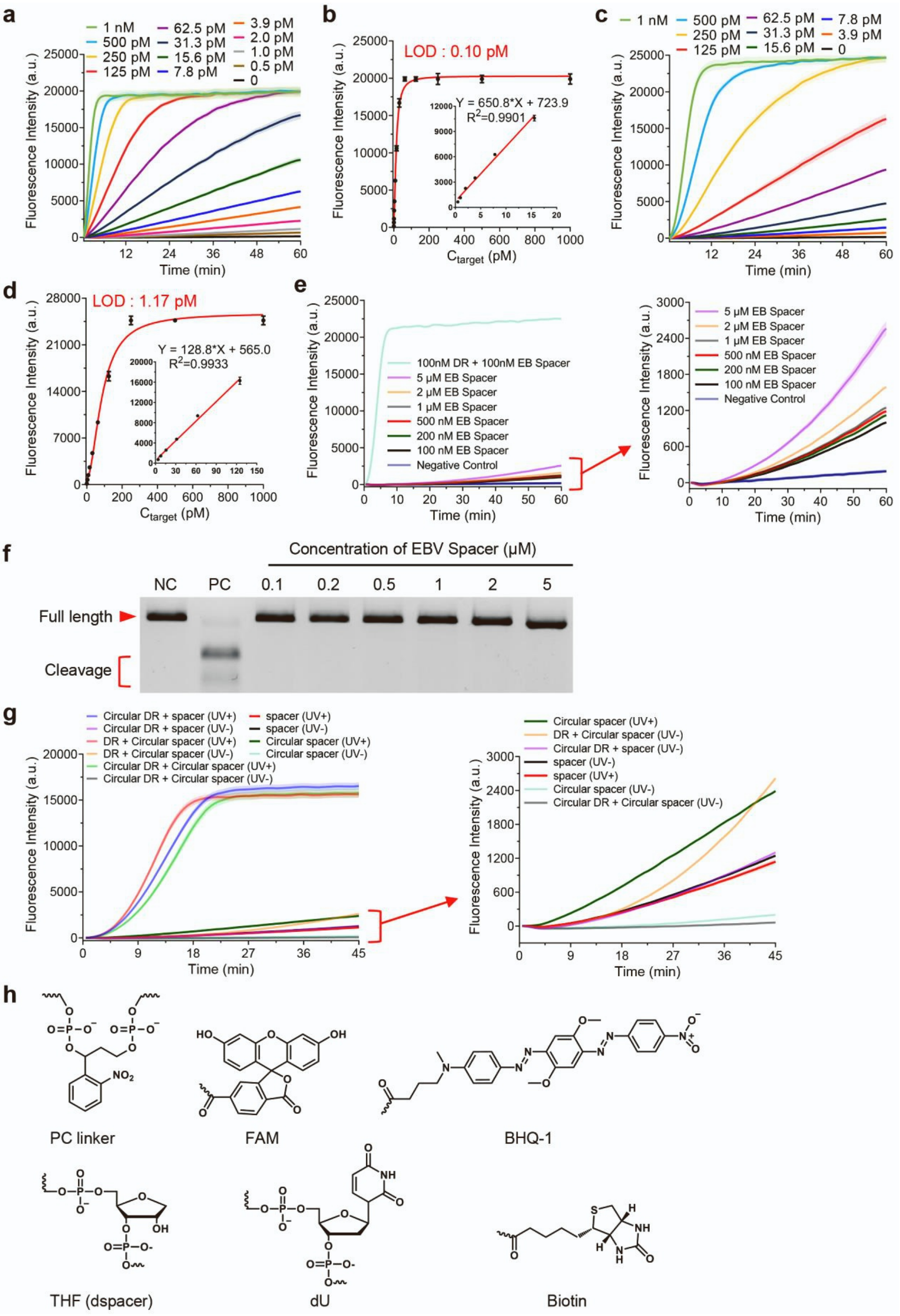

a
b
LOD : 0.10 pM
c
d
LOD : 1.17 pM
e
f
Concentration of EBV Spacer (μM)
NC
PC
Full length
Cleavage
g
h
PC linker
FAM
BHQ-1
THF (dspacer)
dU
Biotin

Figure S9: Evaluation of EBV crRNA and split crRNA systems for target detection, related to Figures 2, 3, 4, 5. (a) Real-time fluorescence intensity of full-length EBV-crRNA detecting EBV Target at varying concentrations under identical experimental conditions. (b) Calibration curve derived from (a), showing the relationship between fluorescence intensity and target concentration, including linear regression analysis and limit of detection (LOD). (c) Real-time fluorescence intensity of split crRNA (DR + EBV Spacer) detecting EBV Target at varying concentrations under identical conditions. (d) Calibration curve derived from (c), displaying the fluorescence-concentration relationship, linear regression, and LOD. All experiments were performed in triplicate, and error bars represent mean ± S.D. (n = 3). (e) Fluorescence comparison of *trans*-cleavage activity of LbCas12a directed by EBV Spacer alone (100 nM-5 μM) versus the combined DR (100 nM) and EBV Spacer (100 nM) system. (f) Agarose gel (2%) analysis of *cis*-cleavage activity of LbCas12a directed by EBV Spacer alone (100 nM-5 μM) versus the combined DR (100 nM) and EBV Spacer (100 nM) system. NC: EBV Target alone, in the absence of both DR and Spacer; PC: positive control (100 nM DR + 100 nM EBV Spacer); 0.1-5: concentrations of EBV Spacer in the absence of both DR (μM). (g) *trans*-cleavage activity comparison of circular DR+Spacer, DR+circular Spacer, and circular DR + circular Spacer under UV exposure and non-exposure conditions. Circular DR+circular Spacer exhibits the lowest background fluorescence. (h) Chemical Structures Corresponding to the Abbreviations of Nucleic Acid Modifications Used in the Manuscript.

Table S1: Oligonucleotide (ODN) sequences and modifications used in this study, related to Figures 1, 2, 3, 4, 5. The abbreviations and modifications include: /ideoxyU/ for deoxyUridine modification, /PC Linker/ for a UV-photocleavable C3 spacer arm linking two nucleotide sequences at any position, /5Phos/ for 5' Phosphorylation modification, /THF/ for dSpacer (1,2'-Dideoxyribose) modification, /56-FAM/ for 5' 6-FAM (Fluorescein) modification, /BHQ1/ for Black Hole Quencher-1 used to quench green and yellow dyes such as FAM, TET, and HEX, and /5mC/ for 5-methyl-2'-deoxycytidine.

Name	Sequence (5'-3')
EBV crRNA	AAU UUC UAC UAA GUG UAG AUC AAA CUC AUA UAU UUG CUG A
EBV-Cir-crRNA precursor	/5Phos/ CAA ACU CAU AUA UUU GCU GA /PC Linker/ A AUU UCU ACU AAG UGU AGA U
DR	AAU UUC UAC UCU UGU AGA U
Circular DR precursor	/5Phos/ACU CUU GUA GAU/PC Linker/ AAU UUC U
EBV spacer	CAA ACU CAU AUA UUU GCU GA
HCV spacer	GGC GUG CCC CCG CAA GAC UG
MPXV spacer	GUA UAU AAG UUG UAC GGC UAA UUC
SEPT spacer	AGC GGG GUU CGA GAG UCG UC
Circular-crRNA-dU precursor	/5Phos/ CAA ACU CAU AUA UUU GCU dGdAdT /ideoxyU/dAdC dAAA UUU CUA CUC UUG UAG AU
Circular-crRNA-THF precursor	/5Phos/ CAA ACU CAU AUA UUU GCU dGdA/THF/dT dAAA UUU CUA CUC UUG UAG AU
F-Q	/56-FAM/-CCC CCC CC-/BHQ1/
AAVS1 crRNA	UAA UUU CUA CUA AGU GUA GAU UCU GUC CCC UCC ACC CCA CAG UG
AAVS1-Cir-crRNA precursor	/5Phos/UCU GUC CCC UCC ACC CCA CAG UG/PC linker/A AUU UCU ACU AAG UGU AGA UC
AAVS1-PCR-F	GTT CTC CTG TGG ATT CGG GTC
AAVS1-PCR-R	TGA CGC ACG GAG GAA CAA TAT
EBV-RPA-F	GAC CCG GCC CAC AAC CTG GCC CAC TAA GGG
EBV-RPA-R	ACT CCA TCG TCA AAG CTG CAC ACA GTC ACC
HCV-RPA-F	GGG TCC TTT CTT GGA TAA ACC CGC TCA ATG C
HCV-RPA-R	CTC GCA AGC ACC CTA TCA GGC AGT ACC ACA A
B6R-RPA-F	CTA ATG CGG AAT GTC AAC CTC TTC AA
B6R-RPA-R	AGA AAA TGT AGA TCC GGA AAT TAA TC
SEPT9-5mC-Target	GGCTGTCCACTCAGTCGGAGGTGAGGAACGACCTCCCTATCCCGTTGC CGGGTCCAAG/5mC/GGGGCC/5mC/GAGAGC/5mC/GC/5mC/GGGGAGAG CCAAAGGGAGGGGACCGATGGATTTCCCAGAGTGAAACTGTGCGTCCT GGAGAGTTCCGAGGCAGCCTCGCGAGCCCTCGAGGAGGTCGCTGTCGC TTGG
SEPT9-C-Target	GGCTGTCCACTCAGTCGGAGGTGAGGAACGACCTCCCTATCCCGTTGC CGGGTCCAAGCGGGGCCCGAGAGCCGCCGGGGAGAGCCAAAGGGAGG GGACCGATGGATTTCCCAGAGTGAAACTGTGCGTCCTGGAGAGTTCCG AGGCAGCCTCGCGAGCCCTCGAGGAGGTCGCTGTCGCTTGG
FAM-Biotin	/56-FAM/-CCC CCC CC-/Biotin/
EBV Target	ATGTCGTATTACACCATTGAGTCGTCTCCCCTTTGGAATGGCCCCTGGA CCCGGCCCACAACCTGGCCCACTAAGGGAGTCCATTGTCTGTTATTTCA TGGTCTTTTTACAAACTCATATATTTGCTGAGGTTTTGAAGGATGCGAT TAAGGACCTTGTTATGACAAAGCCCGCTCCTACCTGCAATATCAAGGT GACTGTGTGCAGCTTTGACGATGGAGTAGATTTGCCTCCCTGGTTTCCA CCTATGGTGGAAGGGGCTGCCGCGGAGGGTGATGACGGAGATGACGG AGATGAAGGAGGTGATGGAGATGAGGGTGAGGAAGGGCAGGAGTGAT GTAACTTGTTAGGAGACGC
MPXV Target	GTAAAACGACGGCCAGTGAATTCGAGCTCGGTACCTCGCGAATGCATC

	TAGAATGAAAACGATTTCCGTTGTTACGTTGTTATGCGTACTACCTGCT<br>GTTGTTTATTCAACATGTACTGTACCCACTATGAATAACGCTAAATTAA<br>CGTCTACCGAAACATCGTTTAATGATAAACAGAAAGTTACGTTTACAT<br>GTGATTCAGGATATCATTCTTTGGATCCAAATGCTGTCTGTGAAACAGA<br>TAAATGGAAATACGAAAATCCATGCAAGAAAATGTGCACAGTTTCTGA<br>TTATGTCTCTGAACTATATGATAAGCCATTATACGAAGTGAATTCCACC<br>ATGACACTAAGTTGCAACGGTGAAACAAAATATTTTCGTTGTGAAGAA<br>AAAAATGGAAATACTTCTTGGAATGATACTGTCACGTGTCCTAATGCG<br>GAATGTCAACCTCTTCAATTAGAACACGGATCGTGTCAACCAGTTAAA<br>GAAAAATACTCATTTGGGGAATATATGACTATCAACTGTGATGTTGGA<br>TATGAGGTTATTGGTGTTTCGTATATAAGTTGTACGGCTAATTCTTGGA<br>ATGTTATTCCATCATGTCAACAAAAATGTGATATACCGTCCCTATCTAA<br>TGGATTAATTTCCGGATCTACATTTTCTATCGGTGGCGTTATACATCTT<br>AGTTGTAAAAGTGGTTTTACACTAACGGGGTCTCCATCATCCACATGTA<br>TCGACGGTAAATGGAATCCCATACTCCCAACATGTGTACGATCTAACG<br>AAGAATTTGATCCAGTGGATGATGGTCCCGACGATGAGACAGATCTGA<br>GCAAACTCTCGAAAGACGTTGTACAATATGAACAAGAAATAGAATCGT<br>TAGAAGCAACTTATCATATAATCATAATGGCGTTGACAATTATGGGTG<br>TCATATTTCTAATCTCCATTATAGTATTAGTTTGTTCCTGTGACAAAAA<br>TAATGACCAATATAAGTTCCATAAATTGCTACCGTGACCCGGGCCCGT<br>CGACTGCAGAGGCCTGCATGCAAGCTTGGCGTAATCATGGTCATAGCT<br>GTTTCCTG
HCV Target	GTAAAACGACGGCCAGTGAATTCGAGCTCGGTACCTCGCGAATGCATC<br>TAGATAATACGACTCACTATAGGGAACTACTGTCTTCACGCAGAAAGC<br>GTCTAGCCATGGCGTTAGTATGAGTGTCGTGCAGCCTCCAGGACCCCC<br>CCTCCCGGGAGAGCCATAGTGGTCTGCGGAACCGGTGAGTACACCGGA<br>ATTGCCAGGACGACCGGGTCCTTTCTTGGATAAACCCGCTCAATGCCT<br>GGAGATTTGGGCGTGCCCCCGCAAGACTGCTAGCCGAGTAGTGTTGGG<br>TCGCGAAAGGCCTTGTGGTACTGCCTGATAGGGTGCTTGCGAGTGCCC<br>CGGGAGGTCTCGTAGACCGTGCACCATGAGCACGAATCCTAAACCTCA<br>AAGACCCGGGCCCGTCGACTGCAGAGGCCTGCATGCAAGCTTGGCGTA<br>ATCATGGTCATAGCTGTTTCCTG
AAVS1 Target	GTTCTCCTGTGGATTCGGGTCACCTCTCACTCCTTTCATTTGGGCAGCT<br>CCCCTACCCCCCTTACCTCTCTAGTCTGTGCTAGCTCTTCCAGCCCCCT<br>GTCATGGCATCTTCCAGGGGTCCGAGAGCTCAGCTAGTCTTCTTCCTCC<br>AACCCGGGCCCTATGTCCACTTCAGGACAGCATGTTTGCTGCCTCCAG<br>GGATCCTGTGTCCCCGAGCTGGGACCACCTTATATTCCCAGGGCCGGTT<br>AATGTGGCTCTGGTTCTGGGTACTTTTATCTGTCCCCTCCACCCCACAG<br>TGGGGCCACTAGGGACAGGATTGGTGACAGAAAAGCCCCCATCCTTAG<br>GCCTCCTCCTTCCTAGTCTCCTGATATTCGTCTAACCCCCACCTCCTGTT<br>AGGCAGATTCCTTATCTGGTGACACACCCCCATTTCCTGGAGCCATCTC<br>TCTCCTTGCCAGAACCTCTAAGGTTTGCTTACGATGGAGCCAGAGAGG<br>ATCCTGGGAGGGAGACTTGGCAGGGGGTGGGAGGGAAGGGGGGGATG<br>CGTGACCTGCCCGGTTCTCAGTGGCCACCCTGCGCTACCCTCTCCCAGA<br>ACCTGAGCTGCTCTGACGCGGCTGTCTGGTGCGTTTCACTGATCCTGGT<br>GCTGCAGCTTCCTTACACTTCCCAAGAGGAGAAGCAGTTTGGAAAAAC<br>AAAATCAGAATAAGTTGGTCCTGAGTTCTAACTTTGGCTCTTCACCTTT<br>CTAGNCCCCAATTTATATTGTTCCTCCGTGCGTCA
